# Construction of *N*-Aryl-Substituted Pyrrolidines by Successive Reductive Amination of Diketones via Transfer Hydrogenation

**DOI:** 10.3390/molecules29112565

**Published:** 2024-05-30

**Authors:** Jianhua Liao, Jinghui Tong, Liang Liu, Lu Ouyang, Renshi Luo

**Affiliations:** 1 School of Pharmaceutical Sciences, Gannan Medical University, Ganzhou 341000, China; liaojianhua715@163.com (J.L.); tongjinghui1756@163.com (J.T.); liuliang19991006@163.com (L.L.); oyl3074@163.com (L.O.); 2 College of Chemistry and Environmental Engineering, Shaoguan University, Shaoguan 512005, China

**Keywords:** transfer hydrogenation, reductive amination, diketones, pyrrolidines

## Abstract

*N*-aryl-substituted pyrrolidines are important moieties widely found in bioactive substances and drugs. Herein, we present a practical reductive amination of diketones with anilines for the synthesis of *N*-aryl-substituted pyrrolidines in good to excellent yields. In this process, the *N*-aryl-substituted pyrrolidines were furnished via successive reductive amination of diketones via iridium-catalyzed transfer hydrogenation. The scale-up performance, water as a solvent, simple operation, as well as derivation of drug molecules showcased the potential application in organic synthesis.

## 1. Introduction

**Scheme 1:**
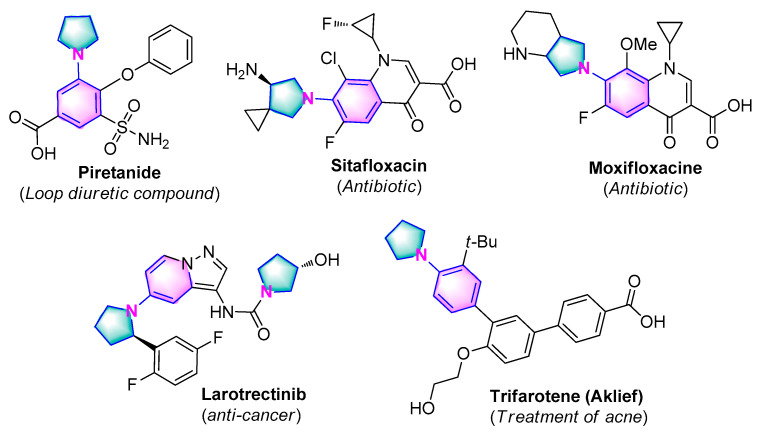
Bioactive molecules with *N*-aryl-substituted pyrrolidine moiety.

*N*-aryl-substituted pyrrolidines are important structural fragments that widely exist in bioactive substances (Scheme 1) [1,2,3,4,5,6,7]. As such, a variety of methods for constructing these compounds have been designed and developed for this purpose [8]. The amination of aromatic amines with dihalogenated compounds [9,10,11,12] or diols [13,14,15] was perceived as a prevalent classical method for pyrrolidine synthesis. Additionally, reductive amination is also one of the general strategies for building *N*-aryl-substituted pyrrolidines [16,17,18,19,20,21,22,23]. Additionally, the hydrogenation of *N*-heteroaromatics was employed as well for the construction of *N*-aryl-substituted pyrrolidines [24,25,26]. It is worth noting that other methods, including cross-coupling of aryl halides [27], reduction in tertiary amides [28] and lactams [29,30], intramolecular C-N coupling [31] or C-N amination [32], reactions of cyclic ether compounds with primary arylamines [33,34,35,36,37] or arylhydrazines [38], Mitsunobu cyclodehydration reaction [39], Prins cyclization [40,41], aminomercuration demercuration [42], as well as intramolecular carbonyl olefination of amides [43], were established to furnish *N*-aryl-substituted pyrrolidines. Furthermore, chiral-substituted pyrrolidines could be delivered via copper-catalyzed asymmetric 1,3-diploar cycloaddition [44].

Despite the great progress achieved in the construction of *N*-aryl-substituted pyrrolidines, challenges still exist in terms of selectivity, substrate versatility, reaction conditions, as well as catalytic efficiency [45,46]. Among the general strategies for pyrrolidine synthesis, reductive amination is undoubtedly the most efficient approach. In this process, the reaction of carbonyl compounds with amines took place first to afford C=N intermediates, which was then followed by the reduction process to form C-N bonds, in which water is the only by-product. Nevertheless, an excess of expensive hydrides or silanes was generally required as a reductant in these traditional processes [16,17,18,19,20,21,22,23]. Therefore, a cheap and efficient catalytic system is still in demand for pyrrolidine synthesis.

**Scheme 2:**
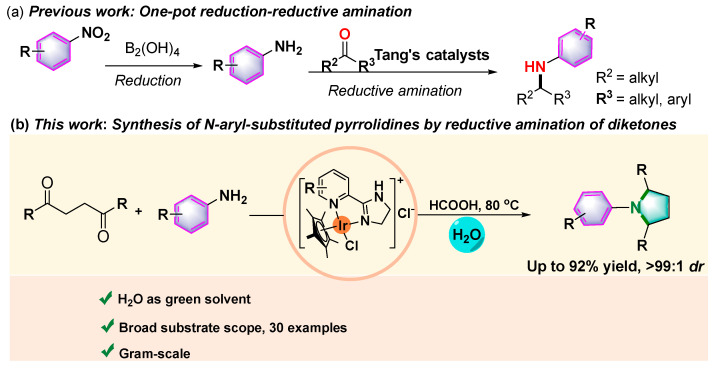
Iridium-catalyzed reductive amination for C-N bond formation.

Metal-catalyzed transfer hydrogenation employed as a supplement to conventional hydrogenation drew much attention and was extensively utilized in organic synthesis [47]. In this context, hydrogen donors such as formic acid were widely utilized in differential transfer hydrogenation reactions owing to their potential hydrogen storage, low toxicity, as well as ease of handling [48]. We have great interest in transfer hydrogenation and conducted a series of relative studies using iridium complexes as catalysts [49], by which reduction in unsaturated compounds [50,51], silane oxidation [52], *N*-allylic alkylation [53], reductive amination [54], reductive amination [55], as well as reductive etherification [56] were reported. Recently, we established a one-pot procedure for synthesizing amines via reduction-reductive amination (Scheme 2a) [57]. Based on previous work, we envisioned that this approach could also be employed for the construction of *N*-aryl-substituted pyrrolidines via successive reductive amination. Herein, we describe an iridium-catalyzed successive reductive amination of diketones with anilines, offering *N*-aryl-substituted pyrrolidines in good to excellent yields under mild conditions (Scheme 2b). This protocol provides a new route for azacycle synthesis.

## 2. Results and Discussion

The development of this iridium-catalyzed reductive amination reaction began with the model reaction of 2,5-hexanedione (**1a**) with aniline (**2a**) (Table 1). Catalyst screening indicated similar yields of mixed products of *N*-phenyl-substituted pyrrolidine **3a1** and *N*-phenyl-substituted pyrrole **3a1′** were formed simultaneously (Table 1, entries 1–6). To afford the appropriate conditions for *N*-phenyl-substituted pyrrolidine synthesis, further reaction conditions were screened. Solvent optimization (Table 1, entries 7–13) showed that water was beneficial to the formation of the desired pyrrolidine **3a1** in 80% yield, in which the pyrrole product **3a1′** was inhibited (Table 1, entry 12). Notably, the increasing loading of HCO_2_H is helpful for the transformation of the desired pyrrolidine product **3a1** (Table 1, entries 14–17). For instance, a 92% isolated yield of *N*-phenyl-substituted pyrrolidine **3a1** was generated in 71:29 *dr* by increasing the dosage of HCO_2_H to 30.0 equivalent (Table 1, entry 17). Similar excellent yields of pyrrolidine **3a1** were afforded by increasing the reaction temperature to 100 °C, even shortening the reaction time (Table 1, entries 18 and 19). In comparison, only 60% yield of **3a1** in 50:50 *dr* was obtained when the reaction was performed at room temperature (Table 1, entry 20). Control experiments showed both the catalyst and formic acid were essential for this transformation (Table 1, entries 21 and 22). 

**Scheme 3:**
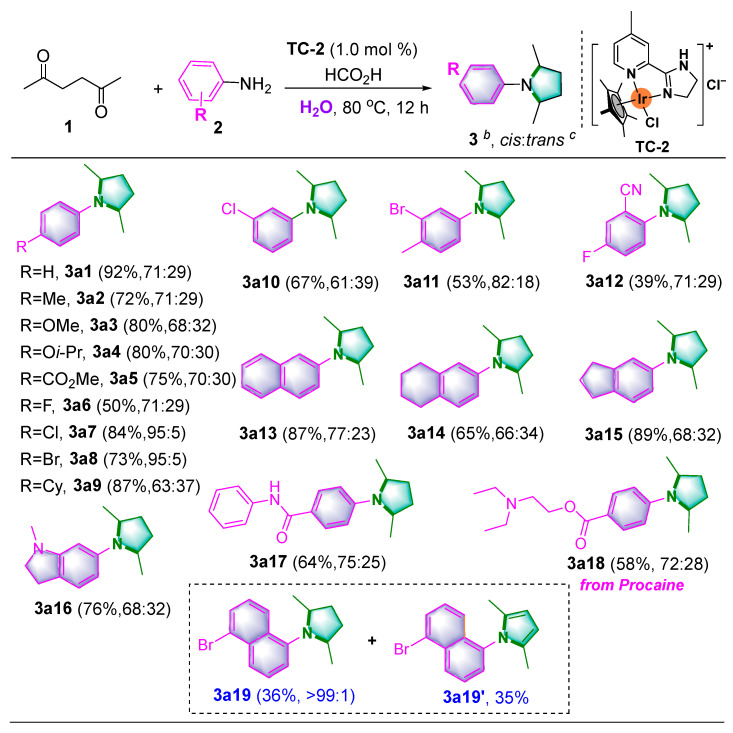
Synthesis of *N*-aryl-substituted pyrrolidines with various amines ^a^. [a] Reaction conditions: **1** (0.5 mmol, 1.0 equiv.), **2** (0.6 mmol, 1.1 equiv.), solvent (2.0 mL), catalyst (1.0 mol%), HCO_2_H (30.0 equiv.), under air, at 80 °C, for 12 h. [b] Isolated yield. [c] Determined by ^1^H NMR analysis.

With the above optimized conditions in hand, differentially substituted aromatic amines were explored for the synthesis of *N*-aryl-substituted pyrrolidines using 2,5-hexanedione (**1a**) as substrate. As showcased in Scheme 3, para-substituted aromatic amines with different electronic effects and positions were well tolerated in this system, affording the desired products of **3a2**–**3a9** in moderate to excellent yields and stereoselectivities. Notably, reductive amination of 2,5-hexanedione (**1a**) with 2-naphthylamine (**2m**), 5,6,7,8-tetrahydro-naphthalen-2-amine (**2n**), 2,3-dihydro-1*H*-inden-5-amine (**2o**), 1-methylindolin-6-amine (**2p**), as well as 4-amino-*N*-phenylbenzamide (**2q**) also produced the corresponding *N*-aryl-substituted pyrrolidines **3a13**–**3a17** in moderate yields and stereoselectivities. Interestingly, similar yield and stereoselectivity of the procaine-derived pyrrolidine **3a18** were afforded as well by using procaine as a nucleophile. However, both pyrrolidine (**3a19**) and pyrrole (**3a19′**) products were formed simultaneously in similar yield with 5-bromonaphthalen-1-amine (**2s**) as substrate.

**Scheme 4:**
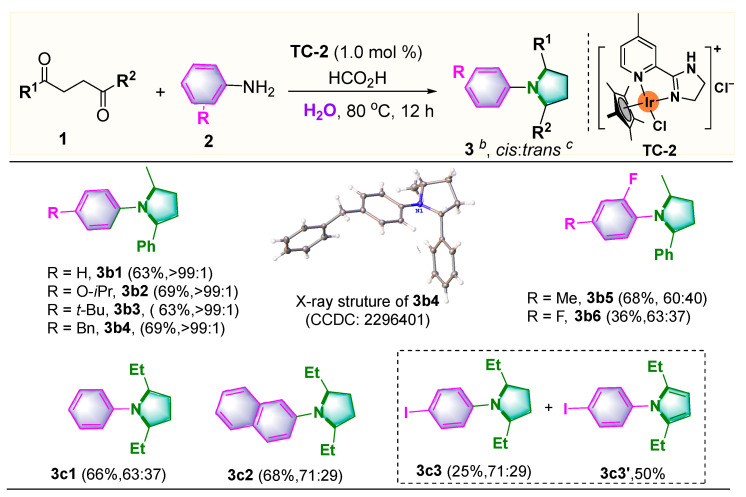
Ir-catalyzed reductive amination of diketones with aromatic amines ^a^. [a] Reaction conditions: **1** (0.5 mmol, 1.0 equiv.), **2** (0.6 mmol, 1.1 equiv.), solvent (2.0 mL), catalyst (1.0 mol%), HCO_2_H (30.0 equiv.), under air, at 80 °C, for 12 h. [b] Isolated yield. [c] Determined by ^1^H NMR analysis.

To further explore the practicability of this catalytic system, the scope of diketones for this reductive amination with different aromatic amines was screened as well. As shown in Scheme 4, similar moderate yields but excellent stereoselectivities (>99:1 *dr*) of *N*-aryl-substituted pyrrolidines (**3b1**, **3b2**~**3b4**) were afforded via reductive amination of 1-phenylhexane-1,4-dione (**1b**) with para-substituted aromatic amines, in which the configuration of **3b4** was determined by X-ray crystallography [58]. However, decreased stereoselectivities of the desired product (**3b5**, **3b6**) were observed using ortho- and para-substituted aromatic amines as nucleophiles and 1-phenylhexane-1,4-dione (**1b**) as substrate. Obviously, employing more steric hinderance of octane-3,6-dione (**1c**) as substrate resulted in similar moderate yields and stereoselectivities (**3c1** and **3c2**). Surprisingly, the formation of pyrrole as a major product (**3c3′**) was observed by using 4-iodoaniline (**2y**) as a nucleophile, delivering a reductive amination product of **3c3** in lower yield.

**Scheme 5:**
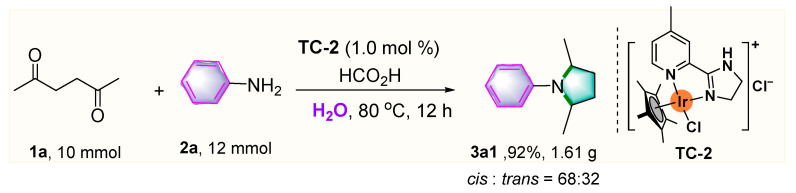
Scale-up experiment.

To further illustrate the utility of this iridium-catalyzed reductive amination, the model reaction was subjected to a gram-scale performance under standard conditions (Scheme 5). As expected, 10.0 mmol of the compound **1a** was successfully converted into 1.61 g of the corresponding product **3a1** in a 92% yield.

**Scheme 6:**
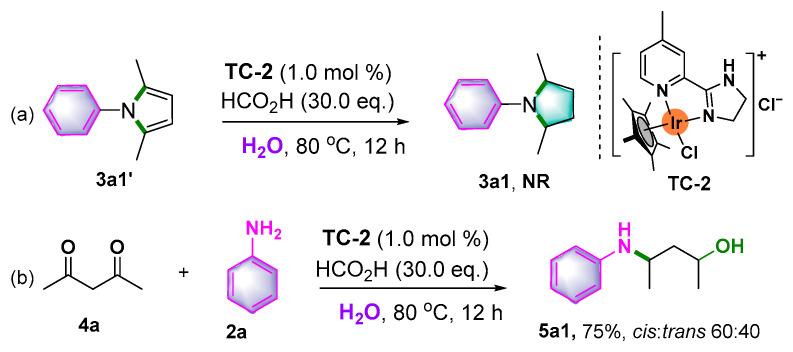
Mechanistic studies on Ir-catalyzed reductive amination. (**a**) The transformation of **3a1′** to **3a1**. (**b**) Ir-catalyzed reductive amination of pentane-2,4-dione with aniline.

To shed light on the process of this Ir-catalyzed reductive amination, control experiments were conducted to explore more details of this transformation. Firstly, to probe whether the pyrrole is involved as an intermediate in this reductive amination process, a control experiment using **3a1′** as substrate was performed under standard conditions (Scheme 6a). However, no expected pyrrolidine product **3a1** was observed, strongly suggesting that the pyrrolidine product was not produced via the transfer hydrogenation of pyrrolidine.

**Scheme 7:**
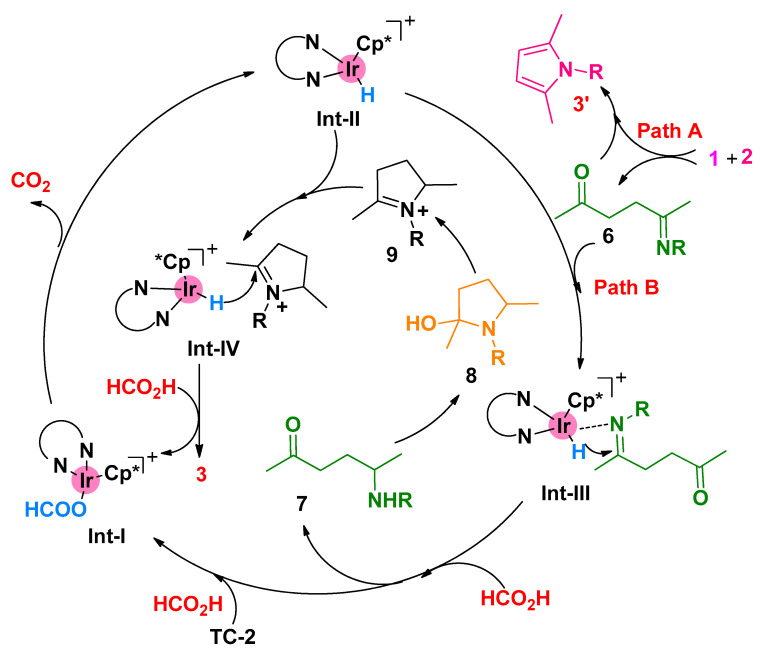
Proposed mechanism. The asterisk “*” represents the excited state.

Based on the experimental results and related studies [16,55,57], a possible process of this Ir-catalyzed successive reductive amination was proposed in Scheme 7. Comparative pathways took place in the presence of diketones and amines under these standard conditions. On the one hand, the Paalknorr condensation took place to deliver the pyrrole product of **3′** (Path A) [59]. On the other hand, the imidone intermediate **6** was formed firstly via condensation, which was reduced to afford the amino ketone **7** via transfer hydrogenation under the action of iridium catalysts (Path B). Using amino ketone **7** as material, a more similar condensation and transfer hydrogenation were undergone to produce the pyrrolidine product **3**. In this process, we envisaged that the stable five-membered ring intermediate **9** was crucial to deliver the corresponding pyrrolidine product, which was supported by the formation of amino alcohol **5a1**, rather than the four-membered ring product using pentane-2,4-dione (**4a**) as substrate (Scheme 6b).

## 3. Experimental Section

All the reactions were carried out in oven-dried Schlenk tubes. All the reagents and anhydrous solvents were purchased from commercial sources and used without further purification. Silica gel (100~200 mesh) bought from commercial sources was used for column chromatography. Purified hexane or a mixture of petroleum ether and ethyl acetate was used as a gradient eluent for column chromatography. ^1^H and ^13^C NMR spectra were recorded using a Bruker DRX-400 spectrometer (Bruker, Ettlingen, Germany) (400 MHz for 1H; 101 MHz for ^13^C). The chemical shifts are referenced to the resonances of the residual protons in the deuterated solvents. The abbreviations [s = singlet, d = doublet, t = triplet, m = multiplet, br = broad coupling constants are given in Hertz (Hz)] were used to designate the chemical shift multiplicities. All NMR quantitative analyses were performed with dimethyl terephthalate as the internal standard. High-resolution mass spectra (HRMS) were recorded by an LCMS-IT-TOF mass spectrometer. Melting points were obtained on the WRR melting point apparatus without correction. Fourier transform infrared spectra (FT-IR) were recorded on a Thermofisher Nicolet iS50 (Thermo, Waltham, MA, USA) spectrometer.

### 3.1. General Procedure for Construction of N-Aryl-Substituted Pyrrolidines

To a 25.0 mL dried Schlenk tube, add catalyst **TC-2** [60] (1.0 mol%), **1** (0.5 mmol, 1.0 equiv.), **2** (0.6 mmol, 1.1 equiv.), solvent (2.0 mL), and hydrogen donor HCO_2_H (30.0 equiv.), which was stirred under air at 80 °C for 12 h. After the completion of the reaction, the mixture was dissolved in ethyl acetate and washed with saturated salt water 2~3 times, and then the organic fraction was dried over anhydrous Na_2_SO_4_. The residue was separated and refined by chromatography on silica gel with hexane or a mixture of petroleum ether and ethyl acetate as the eluent to afford product **3**.

### 3.2. Large-Scale Synthesis of **3a1**

To a 100.0-mL dried round bottom schleck, catalyst **TC-2** (1.0 mol%, 57.92 mg), **1a** (10.0 mmol, 1.17 mL), **2a** (12.0 mmol, 1.09 mL), solvent (30.0 mL), and hydrogen donor HCO_2_H (300.0 mmol, 1.13 mL) were added, which were stirred under air at 80 °C for 12 h. The mixture was diluted with EtOAc (20.0 mL) and then quenched with saturated salt water (30.0 mL). The organic layer was dried over anhydrous Na_2_SO_4_. The residue was purified by chromatography on silica gel with hexane to afford the product **3a1**. 

## 4. Conclusions

Overall, we have developed a practical successive reductive amination of diketones with anilines for the construction of *N*-aryl-substituted pyrrolidines in good to excellent yields and stereoselectivities. This method features scale-up performance, water as a solvent, simple operation, as well as the derivation of drug molecules. Mechanistic studies indicated competitive pathways of Paal-knorr condensation reaction and Ir-catalyzed transfer hydrogenation to produce pyrrole and pyrrolidine products, respectively. Further efforts into the mechanistic details as well as explorations of the medical applications of the *N*-aryl-substituted pyrrolidine products are currently underway in our laboratory.

## Figures and Tables

**Table 1:** Condition optimization of the iridium-catalyzed successive reductive amination for the synthesis of *N*-phenyl-substituted pyrrolidine (**3a1**) ^a^.

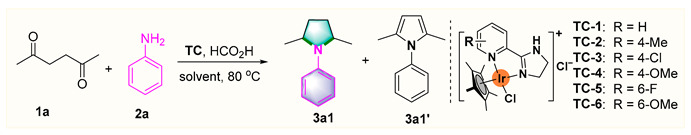						
Entry	Catalyst	Solvent	HCO_2_H (equiv.)	Yield of 3a1 (%) ^b^	Yield of 3a1′ (%) ^b^	*dr* ^c^
1	**TC-1**	toluene	20	41	55	60:40
2	**TC-2**	toluene	20	59	51	59:41
3	**TC-3**	toluene	20	42	52	63:37
4	**TC-4**	toluene	20	48	48	64:36
5	**TC-5**	toluene	20	44	54	50:50
6	**TC-6**	toluene	20	48	48	58:42
7	**TC-2**	DMF	20	<5	65	--
8	**TC-2**	dioxane	20	n.d.	38	--
9	**TC-2**	THF	20	n.d.	76	--
10	**TC-2**	MeOH	20	8	78	50:50
11	**TC-2**	acetone	20	n.d.	23	--
12	**TC-2**	H_2_O	20	80	<5	71:29
13	**TC-2**	DMSO	20	<5	31	--
14	**TC-2**	H_2_O	10	71	<5	66:34
15	**TC-2**	H_2_O	15	84	<5	71:29
16	**TC-2**	H_2_O	25	95	<5	71:29
17	**TC-2**	H_2_O	30	96 (92) ^d^	<5	71:29
18 ^e^	**TC-2**	H_2_O	30	96	<5	71:29
19 ^f^	**TC-2**	H_2_O	30	94	<5	71:29
20 ^g^	**TC-2**	H_2_O	30	60	<5	50:50
21	--	H_2_O	30	n.d.	n.d.	--
22	**TC-2**	H_2_O	--	n.d.	n.d.	--

[a] Reaction conditions: **1a** (0.5 mmol, 1.0 equiv.), **2a** (0.6 mmol, 1.1 equiv.), solvent (2.0 mL), catalyst (1.0 mol%), HCO_2_H, under air, at 80 °C, for 12 h. [b] Determined by ^1^H NMR analysis using dimethyl terephthalate as the internal standard. The yield of **3a1** was determined with a mixture of *cis-trans*. [c] Determined by ^1^H NMR analysis. [d] Isolated yield. [e] At 100 °C. [f] 6 h. [g] At room temperature.

## Data Availability

Data are contained within the Supplementary Materials.

## Supplementary Materials

The following supporting information can be downloaded at: https://www.mdpi.com/article/10.3390/molecules29112565/s1, Figure S1: X-ray crystal structures of compound **3b4**; Table S1: Crystal data and structure refinement for **3b4**.
