# Supplementary material for: Construction of N-Aryl-Substituted Pyrrolidines by Successive Reductive Amination of Diketones via Transfer Hydrogenation
Source: Molecules. 2024 May 30;29(11):2565. doi: 10.3390/molecules29112565 (PMC11173526; doi:10.3390/molecules29112565)
Supplement: Supplementary file 1 [file molecules-29-02565-s001.zip › molecules-3011407-supplementary.pdf]

Construction of *N*-Aryl-Substituted Pyrrolidines by Successive Reductive Amination of Diketones via Transfer Hydrogenation

Jianhua Liao, [a] Jinghui Tong, [a] Liang Liu, [a] Lu Ouyang, [a] Renshi Luo, *[a, b]
[a] *School of Pharmaceutical Sciences, Gannan Medical University, Ganzhou 341000, Jiangxi Province, P. R. China.*
[b] *College of Chemistry and Environmental Engineering, Shaoguan University, Shaoguan 512005, P. R. China. Corresponding author: luorenshi2010@163.com*

Supporting Information

Table of Contents

A. General Information S2

B. General Procedure for Synthesis of compounds 3 ....S2

C. Large scale synthesis of 3a1 ....S3

D. Analysis Data for the Products S3

E. References S14

F. NMR Spectra S15

G. X-ray crystal structure of compound of 3b4 ....S78

H. Crystal structure determination S79

I. IR Spectra S80

A. General information

All the reactions were carried out in oven-~~-~~dried Schlenk tubes. All the reagents and anhydrous solvents were purchased from commercial sources and used without further purification. Silica gel (100~200 mesh) bought from commercial sources was used for column c~~C~~hromatography. Purified hexane or a~~the~~ mixture of petroleum ether and ethyl acetate was~~ere~~ used as a gradient eluent for column chromatography. ^{1}H and ^{13}C NMR spectra were recorded using a Bruker DRX-400 spectrometer (400 MHz for ^{1}H; 101 MHz for ^{13}C)~~,~~. T~~t~~he chemical shifts are referenced to the resonances of the residual protons in the deuterated solvents. The abbreviations [s=singlet, d=doublet, t=triplet, m=multiplet, br=broad coupling constants are given in Hertz (Hz)] were used to designate the chemical shift multiplicities. All NMR quantitative analyse~~i~~s were performed with dimethyl terephthalate as the internal standard. High-resolution mass spectra (HRMS) were recorded by a LCMS-IT-TOF mass spectrometer. Melting points were obtained on the WRR melting point apparatus~~ and~~ without correction. Fourier transform infrared spectra (FT-IR) were recorded on a Thermofisher Nicolet iS50 spectrometer.

B. General Procedure for synthesis of 3

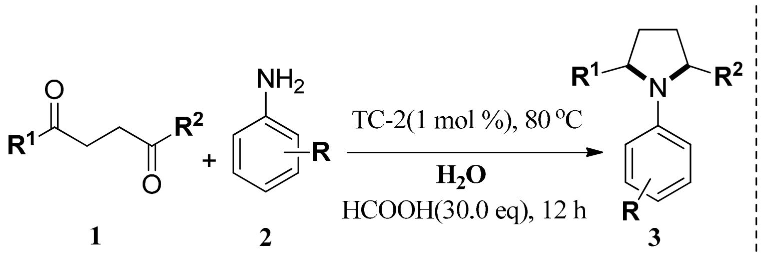

1 **2** **3**

1a: $R^1= R^2=$ Me
1b: $R^1=$ Me, $R^2=$ Ph
1c: $R^1= R^2 =$ Et

To a 25.0-~~-~~mL dried Schlenk tube, ~~-add~~ catalyst **TC-2** (1.0 mol%), **1** (0.5 mmol, 1.0 equiv.), **2** (0.6 mmol, 1.1 equiv.), solvent (2.0 mL), and HCO_2H (30.0 equiv.) were added, which were~~as~~ stirred under air at 80 °C for 12 h. After the completion of the reaction, the mixture was dissolved in ethyl acetate and washed with saturated salt water for 2~3 times, and then the organic fraction was dried over anhydrous Na_2SO_4. The residue was separated and refined by chromatography on silica gel with hexane or a~~the~~ mixture of petroleum ether and ethyl acetate as the eluent to afford product **3**.

C. Large scale synthesis of 3a1

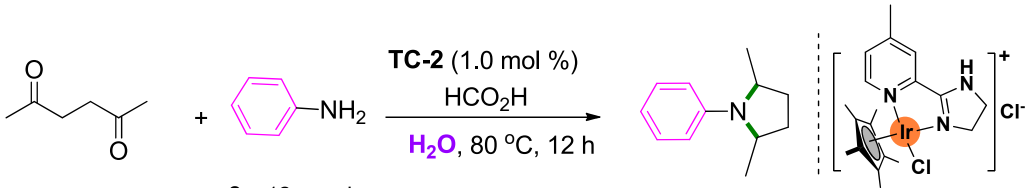

1a, 10 mmol **2a**, 12 mmol **3a1**, 92%, 1.61 g **TC-2**

To a 100.0-~~-~~mL dried round bottom schleck, ~~was added~~ catalyst **TC-2** (1.0 mol%, 57.92 mg), **1a** (10.0 mmol, 1.17 mL), **2a** (12.0 mmol, 1.09 mL), solvent (30.0 mL), and hydrogen donor HCO_2H (300.0 mmol, 1.13 mL) were added, which were~~as~~ stirred under air at 80 °C for 12 h. The mixture was diluted with EtOAc (20.0 mL) and~~,~~ then quenched with saturated salt water (30.0 mL). The organic layer was dried over anhydrous Na_2SO_4. The residue was purified by chromatography on silica gel with hexane to afford the product **3a1**.

D. Analysis Data for the Products

2, 5-Dimethyl-1-phenylpyrrolidine (3a1) [1]**:**

80.5 mg, 92% yield, ***cis:trans***:(71:29). colorless oil. TLC (hexane): R_f = 0.43. ***cis***: ^{1}H NMR (400 MHz, $CDCl_3$) δ 7.23-7.15 (m, 2H), 6.65-6.61 (m, 2H), 6.57 (d, J = 7.2, 1H), 3.79-3.72 (m,2H), 2.07-1.97 (m, 2H), 1.73-1.66 (m, 2H), 1.26 (dd, J = 6.2, 2.2 Hz, 6H).; ^{13}C NMR (101 MHz, $CDCl_3$) δ 147.4, 129.1, 115.3, 111.9, 55.9, 32.4, 21.8. ***trans***: ^{1}H NMR (400 MHz, $CDCl_3$) δ 7.23-7.15 (m, 2H), 6.65-6.61 (m, 2H), 6.57 (d, J = 7.2, 1H), 4.02-3.96 (m, 2H), 2.25-2.19 (m, 2H), 1.61 (dd, J = 5.3, 2.3 Hz, 2H), 1.09 (dd, J = 6.2, 2.2 Hz, 6H); ^{13}C NMR (101 MHz, $CDCl_3$) δ 145.3, 129.1, 114.6, 113.3, 52.7, 30.4, 18.2.

2, 5-Dimethyl-1-(p-tolyl)pyrrolidine (3a2) [1]**:**

67.7 mg, 72% yield, ***cis:trans***: (71:29). Yellow oil. TLC (hexane): R_f = 0.20. ***cis***: ^{1}H NMR (400 MHz, $CDCl_3$) δ 7.01 (t, J = 7.3 Hz, 2H), 6.53 (dd, J = 20.9, 8.6 Hz, 2H), 3.74 - 3.61 (m, 2H), 2.24 (s, 3H), 2.05 - 1.97 (m, 2H), 1.71 - 1.64 (m, 2H), 1.25 (d, J = 6.3 Hz, 6H); ^{13}C NMR (101 MHz, $CDCl_3$) δ 145.5, 129.7, 124.5, 112.2, 56.3, 32.4, 22.1, 20.3. ***trans***: ^{1}H NMR (400 MHz, $CDCl_3$) δ 7.01 (t, J = 7.3 Hz, 2H), 6.53 (dd, J = 20.9, 8.6 Hz, 2H), 3.99 - 3.93 (m, 2H), 2.23 (s, 3H), 2.21 - 2.18 (m, 2H), 1.60 (d, J = 5.2 Hz,

2H), 1.07 (d, $J = 6.1$ Hz, 6H); ^{13}C NMR (101 MHz, $CDCl_3$) δ 143.2, 129.8, 123.6, 113.5, 52.8, 30.5, 20.3, 18.2.

1-(4-Methoxyphenyl)-2, 5-dimethylpyrrolidine (3a3) [1]**:**

83.2 mg, 80% yield, ***cis:trans***: (68:32). Yellow oil. TLC (hexane): $R_f = 0.21$. ***cis***: ^{1}H NMR (400 MHz, $CDCl_3$) δ 6.83 (dtd, $J = 22.1, 5.8, 2.3$ Hz, 2H), 6.63-6.53 (m, 2H), 3.75 (s, 3H), 3.67-3.59 (m, 2H), 2.05 - 1.97 (m, 2H), 1.69-1.64 (m, 2H), 1.24 (d, $J = 6.1$ Hz,6H); ^{13}C NMR (101 MHz, $CDCl_3$) δ 151.0, 142.5, 114.9, 113.7, 57.0, 56.0, 32.4, 22.1. ***trans***: ^{1}H NMR (400 MHz, $CDCl_3$) δ 6.83 (dtd, $J = 22.1, 5.8, 2.3$ Hz, 2H), 6.63-6.53 (m, 2H), 3.96-3.90 (m, 2H), 3.74 (s, 3H), 2.23 - 2.19 (m, 2H), 1.62 - 1.58 (m, 2H), 1.06 (d, $J = 6.3$ Hz, 6H); ^{13}C NMR (101 MHz, $CDCl_3$) δ 150.2, 140.1, 115.0, 114.5, 55.9, 53.1, 30.6, 18.2.

1-(4-Isopropoxyphenyl)-2, 5-dimethylpyrrolidine (3a4):

116.5 mg, 80% yield, ***cis:trans***: (70:30). Pale yellow oil. TLC (hexane): $R_f = 0.29$. ***cis***: ^{1}H NMR (400 MHz, $CDCl_3$) δ 6.85 - 6.79 (m, 2H), 6.60-6.49 (m, 2H), 4.40-4.30 (m, 1H), 3.67-3.60 (m, 2H), 2.05-1.97 (m, 2H), 1.62-1.56 (m, 2H), 1.29 (dd, $J = 6.1, 3.0$ Hz, 6H), 1.24 (d, $J = 6.1$ Hz, 6H); ^{13}C NMR (101 MHz, $CDCl_3$) δ 148.9, 142.7, 118.0, 113.4, 71.3, 56.9, 32.4, 22.3, 22.1. ***trans***: ^{1}H NMR (400 MHz, $CDCl_3$) δ 6.85-6.79 (m, 2H), 6.60-6.49 (m, 2H), 4.40-4.30 (m, 1H), 3.96 - 3.90 (m, 2H), 2.24-2.15 (m, 2H), 1.70-1.64 (m, 2H), 1.29 (dd, $J = 6.1, 3.0$ Hz, 6H), 1.06 (d, $J = 6.1$ Hz, 6H); ^{13}C NMR (101 MHz, $CDCl_3$) δ 148.1, 140.2, 118.1, 114.4, 71.3, 53.0, 30.5, 22.4, 18.3. IR (neat): 2961.76, 1507.10, 1350.58, 1233.64, 1112.43, 955.11, 812.71. HRMS (ESI, m/z): $[M+H]^+$ Calcd. for $C_{15}H_{23}NO$, 234.1858; found, 234.1876.

Methyl-4-(2, 5-dimethylpyrrolidin-1-yl)benzoate (3a5):

115.5 mg, 75% yield, ***cis:trans***: (70:30). Pale yellow oil. TLC (hexane): $R_f = 0.15$. ***cis***: ^{1}H NMR (400 MHz, $CDCl_3$) δ 7.87 (dd, $J = 9.0, 6.0$ Hz, 2H), 6.53 (dd, $J = 14.1, 9.1$ Hz, 2H), 3.88-3.86 (m, 2H), 3.84 (s, 3H), 2.10-2.03 (m, 2H), 1.76-1.70 (m, 2H), 1.26 (d, $J = 6.3$ Hz, 6H); ^{13}C NMR (101 MHz, $CDCl_3$) δ 167.6, 150.2, 131.3, 116.0, 110.9, 55.5, 51.4, 32.1, 21.2. ***trans***: ^{1}H NMR (400 MHz, $CDCl_3$) δ 7.87 (dd, $J = 9.0, 6.0$ Hz, 2H), 6.53 (dd, $J = 14.1, 9.1$ Hz, 2H), 4.06-4.00 (m, 2H), 3.84 (s, 3H), 2.27-2.19 (m, 2H), 1.64 (d, $J = 5.5$ Hz, 2H), 1.10 (d, $J = 6.3$ Hz, 6H); ^{13}C NMR (101 MHz, $CDCl_3$) δ 167.6,

148.8, 131.3, 115.5, 112.2, 53.2, 51.4, 30.1, 18.0. IR (neat): 2963.58, 1700.58, 1598.47, 1519.05, 1433.60, 1367.43, 1272.73, 1177.50, 1105.33, 769.02. HRMS (ESI, m/z): $[M+H]^+$ Calcd. for $C_{14}H_{19}NO_2$, 234.1494; found, 234.1501.

1-(4-Fluorophenyl)-2, 5-dimethylpyrrolidine (3a6):

48.3 mg, 50% yield, ***cis:trans***: (71:29). Yellow oil. TLC (hexane): R_f = 0.56. ***cis*:** ^{1}H NMR (400 MHz, $CDCl_3$) δ 6.96-6.88 (m, 2H), 6.51 (ddd, J = 22.5, 8.3, 4.6 Hz, 2H), 3.71-3.63 (m, 2H), 2.07-1.99 (m, 2H), 1.72-1.66 (m, 2H), 1.25 (d, J = 6.1 Hz, 6H); ^{13}C NMR (101 MHz, $CDCl_3$) δ 154.8 (d, J = 233.6 Hz), 144.2, 115.4 (d, J = 21.8 Hz), 112.7 (d, J = 7.1 Hz), 56.7, 32.4, 21.9. ***trans*:** ^{1}H NMR (400 MHz, $CDCl_3$) δ 6.96 - 6.88 (m, 2H), 6.51 (ddd, J = 22.5, 8.3, 4.6 Hz, 2H), 3.97-3.90 (m, 2H), 2.26-2.18 (m, 2H), 1.62 (d, J = 5.7 Hz, 2H), 1.06 (d, J = 5.0 Hz, 6H); ^{13}C NMR (101 MHz, $CDCl_3$) δ 154.2 (d, J = 233.1 Hz), 141.9, 115.5 (d, J = 21.8 Hz), 113.9 (d, J = 7.1 Hz), 53.1, 30.5, 18.0. IR (neat): 2922.64, 2356.75, 1507.61, 1354.80, 1226.97, 966.11, 807.78. HRMS (ESI, m/z): $[M+H]^+$ Calcd. for $C_{12}H_{16}FN$, 194.1345; found, 194.1361.

1-(4-Chlorophenyl)-2, 5-dimethylpyrrolidine (3a7) [1]:

106.0 mg, 84% yield, ***cis:trans***: (95:5). Colorless oil. TLC (hexane): R_f = 0.60. ***cis*:** ^{1}H NMR (400 MHz, $CDCl_3$) δ 7.14 (dd, J = 9.0, 2.0 Hz, 2H), 6.52 (dd, J = 9.0, 2.1 Hz, 2H), 3.75-3.68 (m, 2H), 2.08-2.00 (m, 2H), 1.73-1.68 (m, 2H), 1.25 (dd, J = 6.2, 1.8 Hz, 6H); ^{13}C NMR (101 MHz, $CDCl_3$) δ 145.9, 128.8, 119.9, 113.0, 56.1, 32.3, 21.6. ***trans*:** ^{1}H NMR (400 MHz, $CDCl_3$) δ 7.14 (dd, J = 9.0, 2.0 Hz, 2H), 6.52 (dd, J = 9.0, 2.1 Hz, 2H), 3.98-3.91 (m, 2H), 2.24-2.18 (m, 2H), 1.64 - 1.62 (m, 2H), 1.07 (dd, J = 6.2, 1.8 Hz, 6H); ^{13}C NMR (101 MHz, $CDCl_3$) δ 145.9, 128.9, 119.9, 114.3, 52.9, 30.4, 18.0.

1-(4-Bromophenyl)-2, 5-dimethylpyrrolidine (3a8) [1]:

93.01 mg, 73% yield, ***cis:trans***: (95:5). Pale yellow oil. TLC (hexane): R_f = 0.71. ***cis*:** ^{1}H NMR (400 MHz, $CDCl_3$) δ 7.26 (dd, J = 8.8, 1.7 Hz, 2H), 6.53-6.45 (m, 2H), 3.75-3.68 (m, 2H), 2.08-2.01 (m, 2H), 1.74-1.68 (m, 2H), 1.25 (dd, J = 6.1, 1.6 Hz, 6H); ^{13}C NMR (101 MHz, $CDCl_3$) δ 146.2, 131.7, 113.5, 106.9, 56.0, 32.3, 21.5. ***trans*:** ^{1}H NMR (400 MHz, $CDCl_3$) δ 7.26 (dd, J = 8.8, 1.7 Hz, 2H), 6.53-6.45 (m, 2H), 3.97-3.91 (m,

2H), 2.24-2.20 (m, 2H), 1.63 (d, *J* = 5.4 Hz, 2H), 1.07 (dd, *J* = 6.2, 1.6 Hz, 6H); ^{13}C NMR (101 MHz, $CDCl_3$) δ 146.2, 131.7, 113.5, 106.9, 52.9, 30.3, 17.9.

1-(4-Cyclohexylphenyl)-2, 5-dimethylpyrrolidine (3a9)

110.8 mg, 87% yield, ***cis:trans***: (63:37). Pale yellow oil. TLC (hexane): R_f = 0.63. ***cis*:** ^{1}H NMR (400 MHz, $CDCl_3$) δ 7.06 (t, *J* = 9.5 Hz, 2H), 6.58 (d, *J* = 8.8 Hz, 1H), 3.77-3.70 (m, 2H), 2.42-2.34 (m, 1H), 2.01 - 1.97 (m, 2H), 1.94-1.79 (m, 5H), 1.69- 1.64 (m, 2H), 1.44-1.33 (m, 5H), 1.26 (d, *J* = 6.3 Hz, 6H); ^{13}C NMR (101 MHz, $CDCl_3$) δ 145.7, 135.1, 127.4, 111.8, 56.2, 43.5, 34.8, 32.4, 27.1, 26.3 (d, *J* = 2.9 Hz), 22.0. ***trans*:** ^{1}H NMR (400 MHz, $CDCl_3$) δ 7.06 (t, *J* = 9.5 Hz, 2H), 6.58 (d, *J* = 8.8 Hz, 2H), 4.00-3.93 (m, 2H), 2.42-2.34 (m, 1H), 2.24-2.16 (m, 2H), 1.94-1.79 (m, 5H), 1.60 (d, *J* = 5.3 Hz, 2H), 1.44-1.33 (m, 5H), 1.08 (d, *J* = 6.3 Hz, 6H); ^{13}C NMR (101 MHz, $CDCl_3$) δ 143.3, 134.2, 127.4, 113.1, 52.8, 43.4, 34.8, 30.4, 29.7, 26.3 (d, *J* = 2.9 Hz), 18.3. IR (neat): 2921.15, 2849.51, 1613.71, 1514.86, 1350.73, 1264.57, 1164.26, 810.89, 735.34, 710.87. HRMS (ESI, m/z): $[M+H]^+$ Calcd. for $C_{18}H_{27}N$, 258.2222; found, 258.2224.

1-(2-Chlorophenyl)-2, 5-dimethylpyrrolidine (3a10):

70.9 mg, 67% yield, ***cis:trans***: (61:39). Colorless oil. TLC (hexane): R_f = 0.60. ***cis*:** ^{1}H NMR (400 MHz, $CDCl_3$) δ 7.09 (q, *J* = 8.3, 7.5 Hz, 1H), 6.61-6.52 (m, 2H), 6.49-6.42 (m, 1H), 3.78 - 3.71 (m, 2H), 2.09-2.01 (m, 2H), 1.75-1.69 (m, 2H), 1.26 (dd, *J* = 6.2, 1.6 Hz, 6H); ^{13}C NMR (101 MHz, $CDCl_3$) δ 148.3, 134.9, 130.0, 115.0, 111.7, 110.1, 55.9, 32.2, 21.5. ***trans*:** ^{1}H NMR (400 MHz, $CDCl_3$) δ 7.09 (q, *J* = 8.3, 7.5 Hz, 1H), 6.61-6.52 (m, 2H), 6.49-6.42 (m, 1H), 3.99-3.93 (m, 2H), 2.26-2.18 (m, 2H), 1.64 (d, *J* = 6.9 Hz, 2H), 1.09 (dd, *J* = 6.1, 1.6 Hz, 6H); ^{13}C NMR (101 MHz, $CDCl_3$) δ 146.4, 134.9, 130.0, 114.4, 112.9, 111.5, 52.9, 30.2, 18.0. IR (neat): 2962.11, 1590.70, 1486.16, 1356.33, 1161.72, 1035.67, 830.22, 753.75, 707.36, 684.17. HRMS (ESI, m/z): $[M+H]^+$ Calcd. for $C_{12}H_{16}ClN$, 210.1050; found, 210.1064.

1-(3-Bromo-4-methylphenyl)-2, 5-dimethylpyrrolidine (3a11):

71.6 mg, 53% yield, ***cis:trans***: (82:18). Pale yellow oil. TLC (hexane): R_f = 0.66. ***cis*:** ^{1}H NMR (400 MHz, $CDCl_3$) δ 7.02 (dd, *J* = 8.4, 2.6 Hz, 1H), 6.77 (dt, *J* = 17.6, 2.8 Hz, 1H), 6.48-6.41 (m, 1H), 3.72-3.65 (m, 2H), 2.27 (s, 3H), 2.05-1.98 (m, 2H), 1.71-1.65 (m, 2H), 1.24 (dd, *J* = 6.3, 2.6 Hz, 6H); ^{13}C NMR (101 MHz, $CDCl_3$) δ 146.7, 130.9,

125.7, 123.8, 115.4, 111.3, 56.1, 32.3, 21.7. ***trans*****:** ^{1}H NMR (400 MHz, $CDCl_3$) δ 7.02 (dd, J = 8.4, 2.6 Hz, 1H), 6.77 (dt, J = 17.6, 2.8 Hz, 1H), 6.48 - 6.41 (m, 1H), 3.95 - 3.89 (m, 2H), 2.28 (s, 3H), 2.21-2.17 (m, 2H), 1.60 (d, J = 7.9 Hz, 2H), 1.06 (dd, J = 6.2, 2.6 Hz, 6H); ^{13}C NMR (101 MHz, $CDCl_3$) δ 144.6, 131.0, 125.7, 123.0, 116.6, 112.7, 52.9, 30.4, 18.1. IR (neat): 2960.94, 1606.29, 1497.77, 1353.47, 1168.74, 1021.05, 831.24, 795.54, 737.89, 680.93. HRMS (ESI, m/z): $[M+H]^+$ Calcd. for $C_{13}H_{18}BrN$, 268.0701; found, 268.0699.

2-(2, 5-Dimethylpyrrolidin-1-yl)-5-fluorobenzonitrile (3a12):

42.7 mg, 39% yield, ***cis:trans***: (71:29). Pale yellow oil. TLC (hexane): R_f = 0.43. ***cis*****:** ^{1}H NMR (400 MHz, $CDCl_3$) δ 7.02 (td, J = 8.8, 3.1 Hz, 1H), 6.77-6.66 (m, 1H), 6.65-6.61 (m, 1H), 3.73-3.65 (m, 2H), 2.13 - 2.05 (m, 2H), 1.78-1.72 (m, 2H), 1.25 (d, J = 6.3 Hz, 6H); ^{13}C NMR (101 MHz, $CDCl_3$) δ 154.6 (d, J = 246.5 Hz), 143.9, 117.6 (d, J = 6.6 Hz), 116.7 (d, J = 20.3 Hz), 115.3, 114.1, 100.9 (d, J = 15.8 Hz), 56.4, 32.2, 21.3; ^{19}F NMR (377 MHz, $CDCl_3$) δ -126.0 (d, J = 3.5 Hz, 1F). ***trans*****:** ^{1}H NMR (400 MHz, $CDCl_3$) δ 7.02 (td, J = 8.8, 3.1 Hz, 1H), 6.77-6.66 (m, 1H), 6.65-6.61 (m, 1H), 3.96 - 3.89 (m, 2H), 2.28-2.19 (m, 2H), 1.68 (d, J = 5.6 Hz, 2H), 1.07 (d, J = 6.1 Hz, 6H); ^{13}C NMR (101 MHz, $CDCl_3$) δ 154.1 (d, J = 246.0 Hz), 142.0, 118.9 (d, J = 6.6 Hz), 116.8 (d, J = 20.3 Hz), 115.2, 114.1, 100.9 (d, J = 15.8 Hz), 53.3, 30.3, 17.7; ^{19}F NMR (377 MHz, $CDCl_3$) δ -126.7 (d, J = 3.1 Hz, 1F). IR (neat): 2964.75, 2232.09, 1608.06, 1504.30, 1367.05, 1240.10, 1163.11, 1037.46. 806.95, 751.40. HRMS (ESI, m/z): $[M+H]^+$ Calcd. for $C_{13}H_{15}FN_2$, 219.1298; found, 219.1314.

2, 5-Dimethyl-1-(naphthalen-2-yl)pyrrolidine (3a13):

97.88 mg, 87% yield, ***cis:trans***: (77:23). Colorless oil. TLC (hexane): R_f = 0.25. ***cis*****:** ^{1}H NMR (400 MHz, $CDCl_3$) δ 7.66-7.57 (m, 3H), 7.31 (t, J = 7.5 Hz, 1H), 7.14-7.10 (m, 1H), 6.99 (td, J = 8.9, 2.6 Hz, 1H), 6.76 (dd, J = 16.3, 2.7 Hz, 1H), 3.91-3.83 (m, 2H), 2.05-1.98 (m, 2H), 1.75-1.67 (m, 2H), 1.31 (d, J = 6.3 Hz, 6H); ^{13}C NMR (101 MHz, $CDCl_3$) δ 145.3, 135.3, 128.8, 127.6, 126.3, 126.2, 125.9, 121.3, 116.2, 105.0, 56.1, 32.4, 22.0. ***trans*****:** ^{1}H NMR (400 MHz, $CDCl_3$) δ 7.66-7.57 (m, 3H), 7.31 (t, J = 7.5 Hz, 1H), 7.14-7.10 (m, 1H), 6.99 (td, J = 8.9, 2.6 Hz, 1H), 6.76 (dd, J = 16.3, 2.7 Hz, 1H), 4.13-4.06 (m, 2H), 2.25-2.19 (m, 2H), 1.62 (d, J = 5.3 Hz, 2H), 1.12 (d, J =

6.3 Hz, 6H); ^{13}C NMR (101 MHz, $CDCl_3$) δ 143.4, 135.5, 128.7, 127.6, 126.1, 125.9, 125.7, 121.2, 117.5, 106.7, 53.1, 30.4, 18.5. IR (neat): 3053.45, 2959.53, 1624.58, 1598.24, 1506.68, 1472.60, 1388.69, 1363.23, 1344.41, 822.86, 800.80, 740.09. HRMS (ESI, m/z): $[M+H]^+$ Calcd. for $C_{16}H_{19}N$, 226.1596; found, 226.1600.

2, 5-Dimethyl-1-(5, 6, 7, 8-tetrahydronaphthalen-2-yl)pyrrolidine (3a14):

114.5 mg, 65% yield, ***cis:trans***: (66:34). Pale yellow oil. TLC (hexane): R_f = 0.49. ***cis***: ^1H NMR (400 MHz, $CDCl_3$) δ 6.91 (t, J = 8.6 Hz, 1H), 6.45 (dd, J = 8.4, 2.8 Hz, 1H), 6.34 (d, J = 2.9 Hz, 1H), 3.75-3.68 (m, 2H), 2.70 (d, J = 27.9 Hz, 4H), 2.02-1.96 (m, 2H), 1.78-1.73 (m, 4H), 1.70-1.63 (m, 2H), 1.25 (d, J = 6.3 Hz, 6H); ^{13}C NMR (101 MHz, $CDCl_3$) δ 145.5, 137.6, 129.7, 124.2, 112.1, 110.3, 56.2, 52.7, 32.4, 30.4, 30.1, 28.4, 23.8, 23.6, 22.1, 18.3. ***trans***: ^1H NMR (400 MHz, $CDCl_3$) δ 6.91 (t, J = 8.6 Hz, 1H), 6.39 (dd, J = 8.4, 2.8 Hz, 1H), 6.28 (d, J = 2.9 Hz, 1H), 4.00-3.93 (m, 2H), 2.70 (d, J = 27.9 Hz, 4H), 2.24-2.16 (m, 2H), 1.78-1.73 (m, 4H), 1.60 (d, J = 5.0 Hz, 2H), 1.08 (d, J = 6.1 Hz, 6H); ^{13}C NMR (101 MHz, $CDCl_3$) δ 143.2, 137.6, 129.7, 123.4, 113.4, 111.6, 56.2, 52.7, 32.4, 30.4, 30.1, 28.4, 23.8, 23.6, 22.1, 18.3. IR (neat): 2921.77, 1611.95, 1505.51, 1354.39, 1162.09, 1037.05, 844.38, 820.33, 792.93, 735.01. HRMS (ESI, m/z): $[M+H]^+$ Calcd. for $C_{16}H_{23}N$, 230.1909; found, 236.1920.

1-(2, 3-Dihydro-1H-inden-5-yl)-2, 5-dimethylpyrrolidine (3a15):

107.5 mg, 89% yield, ***cis:trans***: (68:32). Pale yellow oil. TLC (hexane): R_f = 0.43. ***cis***: ^1H NMR (400 MHz, $CDCl_3$) δ 7.07 (t, J = 7.8 Hz, 1H), 6.52 (d, J = 21.6 Hz, 1H), 6.43 (dd, J = 21.2, 8.2 Hz, 1H), 3.76 - 3.69 (m, 2H), 2.83 (dt, J = 23.0, 7.6 Hz, 4H), 2.07 - 2.03 (m, 2H), 2.01-1.98 (m, 2H), 1.71-1.65 (m, 2H), 1.26 (dd, J = 6.4, 2.0 Hz, 6H); ^{13}C NMR (101 MHz, $CDCl_3$) δ 146.5, 145.3, 131.0, 124.7, 110.3, 108.2, 56.4, 32.4, 31.9, 25.9, 22.0. ***trans***: ^1H NMR (400 MHz, $CDCl_3$) δ 7.07 (t, J = 7.8 Hz, 1H), 6.52 (d, J = 21.6 Hz, 1H), 6.43 (dd, J = 21.2, 8.2 Hz, 1H), 4.01-3.95 (m, 2H), 2.83 (dt, J = 23.0, 7.6 Hz, 4H), 2.23-2.19 (m, 2H), 2.07-2.03 (m, 2H), 1.61 (d, J = 10.0 Hz, 2H), 1.08 (dd, J = 6.4, 2.0 Hz, 6H); ^{13}C NMR (101 MHz, $CDCl_3$) δ 145.3, 144.1, 130.1, 124.7, 111.7, 109.4, 52.9, 33.5, 30.4, 25.7, 18.2. IR (neat): 2956.39, 1615.88, 1495.98, 1352.31, 1166.67, 1037.47, 963.48, 835.89, 797.18. HRMS (ESI, m/z): $[M+H]^+$ Calcd. for $C_{15}H_{21}N$, 216.1752; found, 216.1771.

6-(2, 5-Dimethylpyrrolidin-1-yl)-1-methylindoline (3a16):

81.6 mg, 76% yield, ***cis:trans***: (68:32). Pale yellow oil. TLC (hexane): R_f = 0.33. ***cis*****:** ^{1}H NMR (400 MHz, $CDCl_3$) δ 6.92 (t, J = 7.1 Hz, 1H), 5.97 (dd, J = 8.1, 2.2 Hz, 1H), 5.85 (d, J = 2.2 Hz, 1H), 3.79-3.72 (m, 2H), 3.32-3.18 (m, 2H), 2.83 (t, J = 7.9 Hz, 2H), 2.75 (s, 3H), 2.06-1.98 (m, 2H), 1.73-1.63 (m, 2H), 1.27 (d, J = 6.2 Hz, 6H); ^{13}C NMR (101 MHz, $CDCl_3$) δ 154.4, 147.7, 124.5, 117.6, 101.7, 92.8, 56.9, 56.3, 53.0, 36.4, 32.4, 30.4, 27.9, 22.1, 18.4. ***trans*****:** ^{1}H NMR (400 MHz, $CDCl_3$) δ 6.92 (t, J = 7.1 Hz, 1H), 5.93 (dd, J = 8.0, 2.2 Hz, 1H), 5.81 (d, J = 2.2 Hz, 1H), 4.01-3.95 (m, 2H), 3.33 - 3.17 (m, 2H), 2.83 (t, J = 7.9 Hz, 2H), 2.74 (s, 3H), 2.23-2.17 (m, 2H), 1.59 (d, J = 5.0 Hz, 2H), 1.10 (d, J = 6.1 Hz, 6H); ^{13}C NMR (101 MHz, $CDCl_3$) δ 154.4, 145.5, 124.5, 116.8, 103.2, 94.2, 56.8, 56.3, 53.0, 36.5, 32.4, 30.4, 27.9, 22.1, 18.4. IR (neat): 2958.32, 1623.52, 1496.60, 1346.64, 1262.78, 1240.95, 1076.50, 792.21, 733.19, 699.58. HRMS (ESI, m/z): $[M+H]^+$ Calcd. for $C_{15}H_{22}N_2$, 231.1861; found, 231.1861.

4-(2, 5-Dimethylpyrrolidin-1-yl)-*N*-phenylbenzamide (3a17):

85.2 mg, 64% yield, ***cis:trans***: (70:30). Gray solid. mp. 172-174 °C. TLC (hexane): R_f = 0.28. ***cis*****:** ^{1}H NMR (400 MHz, $CDCl_3$) δ 7.85-7.76 (m, 3H), 7.52-7.48 (m, 1H), 7.45-7.42 (m, 4H), 6.60-6.54 (m, 2H), 3.78-3.71 (m, 2H), 2.08-2.04 (m, 2H), 1.74-1.62 (m, 2H), 1.27 (dd, J = 6.3, 2.1 Hz, 6H); ^{13}C NMR (101 MHz, $CDCl_3$) δ 165.6, 145.0, 135.4, 131.4, 128.7, 127.0, 126.3, 122.5, 112.1, 56.2, 32.4, 21.8. ***trans*****:** ^{1}H NMR (400 MHz, $CDCl_3$) δ 7.85- 7.76 (m, 3H), 7.52-7.48 (m, 1H), 7.45-7.42 (m, 4H), 6.60-6.54 (m, 2H), 4.02-3.95 (m, 2H), 2.25-2.20 (m, 2H), 1.63 (d, J = 5.1 Hz, 2H), 1.09 (dd, J = 6.1, 2.1 Hz, 6H). ^{13}C NMR (101 MHz, $CDCl_3$) δ 165.6, 142.9, 135.4, 131.4, 128.7, 127.0, 125.7, 122.5, 113.4, 52.9, 30.4, 18.2. IR (neat): 2960.64, 1636.90, 1512.71, 1322.47, 1263.84, 1026.93, 895.12, 811.09, 732.40, 703.05. HRMS (ESI, m/z): $[M+H]^+$ Calcd. for $C_{19}H_{22}N_2O$, 295.1810; found, 295.1827.

2-(Diethylamino)ethyl 4-(2, 5-dimethylpyrrolidin-1-yl)benzoate (3a18):

92.4 mg, 58% yield, ***cis:trans***: (72:28). Pale yellow oil. TLC (petroleum ether/ethyl acetate = 2/1, v/v): R_f = 0.45. ***cis*****:** ^{1}H NMR (400 MHz, $CDCl_3$) δ 7.94-7.81 (m, 2H), 6.61-6.48 (m, 2H), 4.34 (td, J = 6.3, 2.2 Hz, 2H), 3.91-3.84 (m, 2H), 2.85 (t, J = 6.3 Hz, 2H), 2.64 (q, J = 7.1 Hz, 4H), 2.13-2.05 (m, 2H), 1.80-1.72 (m, 2H), 1.28 (d, J = 6.2

Hz, 6H), 1.08 (t, J = 7.1 Hz, 6H); ^{13}C NMR (101 MHz, $CDCl_3$) δ 167.0, 150.2, 131.4 , 116.1, 110.9, 62.4, 55.5, 51.0, 47.7, 32.1, 21.2, 12.0. ***trans*:** ^{1}H NMR (400 MHz, $CDCl_3$) δ 7.94-7.81 (m, 2H), 6.61-6.48 (m, 2H), 4.34 (td, J = 6.3, 2.2 Hz, 2H), 4.08-4.02 (m, 2H), 2.85 (t, J = 6.3 Hz, 2H), 2.64 (q, J = 7.1 Hz, 4H), 2.28-2.22 (m, 2H), 1.67 (d, J = 5.6 Hz, 2H), 1.12 (d, J = 6.3 Hz, 6H), 1.08 (t, J = 7.1 Hz, 6H); ^{13}C NMR (101 MHz, $CDCl_3$) δ 148.8, 131.4, 115.6, 112.2, 53.1, 51.0, 47.7, 30.1, 18.1, 12.0. IR (neat): 2962.43, 2357.91, 1701.70, 1604.25, 1520.43, 1373.44, 1273.49, 1181.56, 1107.44, 1082.16, 769.05. HRMS (ESI, m/z): $[M+H]^+$ Calcd. for $C_{19}H_{30}N_2O_2$, 319.2386; found, 319.2376.

1-(5-Bromonaphthalen-1-yl)-2, 5-dimethylpyrrolidine (3a19):

54.5 mg, 36% yield, ***cis:trans*** (> 99:1). Pale yellow oil. TLC (hexane): R_f = 0.80. ***cis*:** ^{1}H NMR (400 MHz, $CDCl_3$) δ 8.67 (d, J = 8.5 Hz, 1H), 8.11 (d, J = 8.4 Hz, 1H), 7.77 (d, J = 7.4 Hz, 1H), 7.61-7.57 (m, 1H), 7.50 (d, J = 7.5 Hz, 1H), 7.29-7.23 (m, 1H), 3.27 (q, J = 6.1 Hz, 2H), 2.14-2.03 (m, 2H), 1.68 - 1.58 (m, 2H), 0.84 (dd, J = 6.1, 1.8 Hz, 6H); ^{13}C NMR (101 MHz, $CDCl_3$) δ 148.1, 136.9, 132.7, 130.1, 127.4, 125.3, 124.9, 124.4, 122.4, 121.4, 63.5, 32.1, 20.2. IR (neat): 2959.60, 1586.59, 1495.73, 1389.09, 1259.43, 1142.00, 923.54, 784.04, 735.21. HRMS (ESI, m/z): $[M+H]^+$ Calcd. for $C_{16}H_{18}BrN$, 304.0701; found, 304.6693.

1-(5-Bromonaphthalen-1-yl)-2, 5-dimethyl-1H-pyrrole (3a19'):

52.33 mg, 35% yield, white oil. TLC (hexane): R_f = 0.45. ^{1}H NMR (400 MHz, $CDCl_3$) δ 8.35 (d, J = 8.6 Hz, 1H), 7.81 (d, J = 7.4 Hz, 1H), 7.67 (t, J = 7.9 Hz, 1H), 7.48 (d, J = 7.3 Hz, 1H), 7.29-7.24 (m, 1H), 7.07 (d, J = 8.5 Hz, 1H), 6.00 (s, 2H), 1.87 (s, 6H); ^{13}C NMR (101 MHz, $CDCl_3$) δ 136.1, 133.3, 132.8, 130.7, 130.0, 127.9, 127.6, 127.1, 126.8, 123.3, 123.1, 105.7, 12.6. IR (neat): 2915.92, 1667.15, 1594.73, 1497.89, 1411.64, 1020.04, 964.45, 785.37, 725.03. HRMS (ESI, m/z): $[M+H]^+$ Calcd. for $C_{16}H_{14}BrN$, 300.0388; found, 300.0366.

2-Methyl-1, 5-diphenylpyrrolidine (3b1): [2]

74.66 mg, 63% yield, ***cis:trans*** (> 99:1). Colorless oil. TLC (hexane): R_f = 0.61. ^{1}H NMR (400 MHz, $CDCl_3$) δ 7.33-7.27 (m, 4H), 7.22-7.18 (m, 1H), 7.14-7.09 (m, 2H), 6.63 (t, J = 7.3 Hz, 1H), 6.52 (d, J = 8.8 Hz, 2H), 4.62 (t, J = 7.0 Hz, 1H), 4.00 - 3.95

(m, 1H), 2.38-2.29 (m, 1H), 2.11-2.03 (m, 1H), 1.97-1.89 (m, 1H), 1.70-1.63 (m, 1H), 1.44 (dd, J = 6.3, 1.6 Hz, 3H); ^{13}C NMR (101 MHz, $CDCl_3$) δ 147.5, 145.1, 129.0, 128.7, 126.7, 125.8, 116.2, 113.0, 66.2, 56.4, 35.4, 32.5, 21.1.

1-(4-Isopropoxyphenyl)-2-methyl-5-phenylpyrrolidine (3b2):

101.78 mg, 69% yield, ***cis:trans*** (> 99:1). Pale yellow oil. TLC (hexane): R_f = 0.49. ^{1}H NMR (400 MHz, $CDCl_3$) δ 7.35-7.28 (m, 4H), 7.23-7.19 (m, 1H), 6.73-6.71 (m, 2H), 6.46-6.43 (m, 2H), 4.55 (t, J = 6.8 Hz, 1H), 4.35-4.25 (m, 1H), 3.94-3.87 (m, 1H), 2.36-2.28 (m, 1H), 2.12-2.02 (m, 1H), 1.94-1.86 (m, 1H), 1.69-1.62 (m, 1H), 1.43 (d, J = 6.3 Hz, 3H), 1.25 (d, J = 6.1 Hz, 6H); ^{13}C NMR (101 MHz, $CDCl_3$) δ 149.1, 145.5, 142.5, 128.6, 126.6, 125.9, 117.6, 113.6, 71.1, 66.8, 56.8, 35.4, 32.5, 22.3, 21.4. IR (neat): 2965.01, 1506.75, 1234.20, 1109.63, 955.73, 813.57, 742.93, 699.46. HRMS (ESI, m/z): $[M+H]^+$ Calcd. for $C_{20}H_{25}NO$, 296.2014; found, 296.2027.

1-(4-(*tert*-Butyl)phenyl)-2-methyl-5-phenylpyrrolidine (3b3):

92.3 mg, 63% yield, ***cis:trans*** (> 99:1). Pale yellow oil. TLC (hexane): R_f = 0.70. ^{1}H NMR (400 MHz, $CDCl_3$) δ 7.36-7.28 (m, 4H), 7.23-7.15 (m, 3H), 6.49 (dd, J = 8.9, 2.4 Hz, 2H), 4.60 (t, J = 5.9 Hz, 1H), 4.01-3.93 (m, 1H), 2.35-2.27 (m, 1H), 2.10-2.01 (m, 1H), 1.96-1.87 (m, 1H), 1.70-1.62 (m, 1H), 1.45 (dd, J = 6.2, 2.4 Hz, 3H), 1.24 (d, J = 2.6 Hz, 9H); ^{13}C NMR (101 MHz, $CDCl_3$) δ 145.5, 145.4, 138.7, 128.7, 126.7, 125.9, 125.8, 112.6, 66.5, 56.6, 35.5, 33.8, 32.5, 31.7, 21.3. IR (neat): 2959.87, 1611.77, 1516.28, 1348.68, 1264.91, 1203.50, 812.94, 736.08, 698.99. HRMS (ESI, m/z): $[M+H]^+$ Calcd. for $C_{21}H_{27}N$, 294.2222; found, 294.2224.

1-(4-Benzylphenyl)-2-methyl-5-phenylpyrrolidine (3b4):

112.1 mg, 69% yield, ***cis:trans*** (> 99:1). Pale yellow oil. TLC (hexane): R_f = 0.74. ^{1}H NMR (400 MHz, $CDCl_3$) δ 7.30 (d, J = 6.7 Hz, 4H), 7.27-7.16 (m, 3H), 7.18-7.09 (m, 3H), 6.97-6.90 (m, 2H), 6.45 (dd, J = 8.5, 1.8 Hz, 2H), 4.58 (t, J = 7.0 Hz, 1H), 3.99-3.91 (m, 1H), 3.82 (s, 2H), 2.35-2.27 (m, 1H), 2.10-2.01 (m, 1H), 1.95-1.86 (m, 1H), 1.68 - 1.61 (m, 1H), 1.42 (dd, J = 6.2, 1.7 Hz, 3H); ^{13}C NMR (101 MHz, $CDCl_3$) δ 146.0, 145.2, 142.1, 129.4, 128.9, 128.7, 128.4, 126.7, 125.8 (d, J = 3.2 Hz), 113.0, 66.3, 56.6, 41.0, 35.4, 32.4, 21.2. IR (neat): 2964.16, 2357.56, 1614.05, 1514.99,

1264.05, 731.74, 699.16. HRMS (ESI, m/z): $[M+H]^+$ Calcd. for $C_{24}H_{25}N$, 328.2065; found, 328.2060.

1-(2-Fluoro-4-methylphenyl)-2-methyl-5-phenylpyrrolidine (3b5):

91.46 mg, 68% yield, ***cis:trans*** (60:40). Colorless oil. TLC (hexane): R_f = 0.64. ***cis***: ^{1}H NMR (400 MHz, $CDCl_3$) δ 7.36 (d, J = 7.3 Hz, 1H), 7.28 (t, J = 7.6 Hz, 1H), 7.23-7.10 (m, 3H), 6.78-6.69 (m, 1H), 6.66 (d, J = 8.3 Hz, 1H), 6.55 (t, J = 8.8 Hz, 1H), 4.64 (t, J = 7.0 Hz, 1H), 4.02-3.93 (m, 1H), 2.34-2.21 (m, 1H), 2.18 (s, 3H), 2.06 -1.97 (m, 1H), 1.92-1.84 (m, 1H), 1.68-1.57 (m, 1H), 1.39 (d, J = 6.4 Hz, 3H); ^{13}C NMR (101 MHz, $CDCl_3$) δ 154.6 (d, J = 21.3 Hz), 145.3, 134.1 (d, J = 8.9 Hz), 131.7 (d, J = 9.8 Hz), 128.9 (d, J = 7.1 Hz), 128.3, 126.5, 126.0, 124.6 (d, J = 2.7 Hz), 119.4 (d, J = 5.4 Hz), 117.2, 117.0, 67.2 (d, J = 1.9 Hz), 58.7 (d, J = 4.3 Hz), 34.9, 32.4, 21.9 (d, J = 4.6 Hz), 19.5; ^{19}F NMR (377 MHz, $CDCl_3$) δ -124.3 (s, 1F). ***trans***: ^{1}H NMR (400 MHz, $CDCl_3$) δ 7.36 (d, J = 7.3 Hz, 1H), 7.28 (t, J = 7.6 Hz, 1H), 7.23-7.10 (m, 3H), 6.78-6.69 (m, 1H), 6.61 (d, J = 8.3 Hz, 1H), 6.49 (t, J = 8.8 Hz, 1H), 4.88 (t, J = 6.6 Hz, 1H), 4.49-4.44 (m, 1H), 2.49-2.40 (m, 1H), 2.34-2.21 (m, 1H), 2.15 (s, 3H), 1.82 - 1.74 (m, 1H), 1.68-1.57 (m, 1H), 1.06 (d, J = 6.2 Hz, 3H); ^{13}C NMR (101 MHz, $CDCl_3$) δ 152.2 (d, J = 21.2 Hz), 144.6, 134.1 (d, J = 8.9 Hz), 131.7 (d, J = 9.8 Hz), 128.9 (d, J = 7.1 Hz), 128.3, 126.5, 126.4, 124.4 (d, J = 2.7 Hz), 118.5 (d, J = 5.0 Hz), 116.7, 116.5, 63.0 (d, J = 2.1 Hz), 56.0 (d, J = 6.6 Hz), 34.6, 31.7, 20.2 (d, J = 7.5 Hz), 19.5; ^{19}F NMR (377 MHz, $CDCl_3$) δ -124.3 (s, 1F). IR (neat): 2962.78, 1626.32, 1514.29, 1449.39,1235.88, 1139.84, 796.61, 738.48, 698.83. HRMS (ESI, m/z): $[M+H]^+$ Calcd. for $C_{18}H_{20}FN$, 270.1658; found, 270.1671.

1-(2, 4-Difluorophenyl)-2-methyl-5-phenylpyrrolidine (3b6):

49.2 mg, 39% yield, ***cis:trans*** (63:37). Colorless oil. TLC (hexane): R_f = 0.69. ***cis***: ^{1}H NMR (400 MHz, $CDCl_3$) δ 7.35 (d, J = 7.8 Hz, 1H), 7.30 (d, J = 7.4 Hz, 1H), 7.27-7.21 (m, 1H), 7.20 (s, 1H), 7.18-7.12 (m, 1H), 6.75-6.67 (m, 1H), 6.66-6.60 (m, 1H), 6.58-6.50 (m, 1H), 4.58 (t, J = 7.0 Hz, 1H), 4.45-4.37 (m, 1H), 2.48-2.40 (m, 1H), 2.35-2.24 (m, 1H), 1.82-1.75 (m, 1H), 1.71-1.60 (m, 1H), 1.05 (d, J = 6.1 Hz, 3H); ^{13}C NMR (101 MHz, $CDCl_3$) δ 157.3 (d, J = 11.2 Hz), 154.9 (d, J = 11.3 Hz), 144.8, 133.1 (dd, J = 9.2, 3.3 Hz), 128.3, 126.7, 126.2, 120.2 (dd, J = 9.0, 5.8 Hz), 110.6 (d, J = 3.5 Hz),

104.8 (d, J = 25.8 Hz), 67.6 (d, J = 1.9 Hz), 59.0 (d, J = 4.0 Hz), 34.9, 32.4, 21.6 (d, J = 4.2 Hz); ^{19}F NMR (377 MHz, $CDCl_3$) δ -119.3 (d, J = 2.7 Hz, 1F), -122.9 (d, J = 2.5 Hz, 1F). ***trans*****:** ^{1}H NMR (400 MHz, $CDCl_3$) δ 7.35 (d, J = 7.8 Hz, 1H), 7.30 (d, J = 7.4 Hz, 1H), 7.27-7.21 (m, 1H), 7.20 (s, 1H), 7.18-7.12 (m, 1H), 6.75-6.67 (m, 1H), 6.66-6.60 (m, 1H), 6.58-6.50 (m, 1H), 4.83 (t, J = 6.9 Hz, 1H), 3.91-3.85 (m, 1H), 2.35-2.24 (m, 1H), 2.08-2.00 (m, 1H), 1.92-1.84 (m, 1H), 1.71-1.60 (m, 1H), 1.35 (d, J = 6.1 Hz, 3H); ^{13}C NMR (101 MHz, $CDCl_3$) δ 156.7 (d, J = 11.5 Hz), 155.4 (d, J = 11.3 Hz), 144.0, 130.8 (d, J = 7.0 Hz), 128.3, 126.7, 126.4, 119.8 (dd, J = 8.9, 6.2 Hz), 110.2 (d, J = 3.4 Hz), 104.1 (d, J = 25.8 Hz), 63.3 (d, J = 2.2 Hz), 56.0 (d, J = 6.1 Hz), 34.9, 32.1, 19.4; ^{19}F NMR (377 MHz, $CDCl_3$) δ -119.5 (d, J = 2.8 Hz, 1F), -124.0 (d, J = 2.8 Hz, 1F). IR (neat): 2963.51, 2358.07, 1684.30, 1507.16, 1262.84, 1132.77, 966.77, 845.14, 756.28, 699.06. HRMS (ESI, m/z): $[M+H]^+$ Calcd. for $C_{17}H_{17}F_2N$, 274.1407; found, 274.1420.

2,5-Diethyl-1-phenylpyrrolidine(3c1):

67.0 mg, 66% yield, ***cis:trans*** (63:37). Pale yellow oil. TLC (hexane): R_f = 0.76. ***cis*****:** ^{1}H NMR (400 MHz, $CDCl_3$) δ 7.20 (q, J = 6.9 Hz, 2H), 6.65-6.60 (m, 1H), 6.57 (d, J = 8.4 Hz, 2H), 3.54-3.49 (m, 2H), 2.05-1.93 (m, 2H), 1.90-1.70 (m, 4H), 1.36-1.27 (m, 2H), 0.93 (t, J = 7.4 Hz, 6H); ^{13}C NMR (101 MHz, $CDCl_3$) δ 147.7, 129.1, 115.3, 111.9, 62.1, 29.2, 27.9, 10.7. HRMS (ESI, m/z): $[M+H]^+$ Calcd. for $C_{14}H_{21}N$, 204.1752; found, 204.1747. ***trans*****:** ^{1}H NMR (400 MHz, $CDCl_3$) δ 7.20 (q, J = 6.9 Hz, 2H), 6.65-6.60 (m, 1H), 6.49 (d, J = 8.5 Hz, 2H), 3.66-3.62 (m, 2H), 2.05-1.93 (m, 2H), 1.90-1.70 (m, 4H), 1.36-1.27 (m, 2H), 0.88 (t, J = 7.4 Hz, 6H); ^{13}C NMR (101 MHz, $CDCl_3$) δ 145.2, 129.2, 114.6, 113.5, 59.4, 27.2, 23.8, 11.1. IR (neat): 2958.69, 1594.44, 1500.13, 1360.84, 744.42, 693.09. HRMS (ESI, m/z): $[M+H]^+$ Calcd. for $C_{14}H_{21}N$, 204.1752; found, 204.1747.

2,5-Diethyl-1-(naphthalen-2-yl)pyrrolidine (3c2):

86.0 mg, 68% yield, ***cis:trans*** (71:29). Pale yellow oil. TLC (hexane): R_f = 0.69. ***cis*****:** ^{1}H NMR (400 MHz, $CDCl_3$) δ 7.67-7.63 (m, 2H), 7.62-7.57 (m, 1H), 7.31 (t, J = 7.7 Hz, 1H), 7.12 (t, J = 7.5 Hz, 1H), 7.00-6.91 (m, 1H), 6.73 (s, 1H), 3.68-3.62 (m, 2H), 2.10-1.74 (m, 6H), 1.43-1.31 (m, 2H), 0.97 (td, J = 7.5, 2.3 Hz, 6H); ^{13}C NMR (101

MHz, $CDCl_3$) δ 145.5, 135.2, 128.7, 127.5, 126.1, 125.9, 125.7, 121.3, 116.2, 104.9, 62.1, 29.2, 27.9, 10.7. ***trans***: ^{1}H NMR (400 MHz, $CDCl_3$) δ 7.67-7.63 (m, 2H), 7.62-7.57 (m, 1H), 7.31 (t, J = 7.7 Hz, 1H), 7.12 (t, J = 7.5 Hz, 1H), 7.00-6.91 (m, 1H), 6.66 (s, 1H), 3.81-3.76 (m, 2H), 2.10-1.74 (m, 6H), 1.43 - 1.31 (m, 2H), 0.91 (td, J = 7.5, 2.2 Hz, 6H); ^{13}C NMR (101 MHz, $CDCl_3$) δ 143.3, 135.4, 128.7, 127.6, 126.2, 126.1, 125.7, 121.2, 117.6, 106.8, 59.6, 27.3, 24.2, 11.0. IR (neat): 2957.74, 1625.05, 1598.14, 1507.24, 1472.18, 1366.38, 821.02, 802.58, 739.14. HRMS (ESI, m/z): $[M+H]^+$ Calcd. for $C_{18}H_{23}N$, 254.1909; found, 254.1922.

2, 5-Diethyl-1-(4-iodophenyl)pyrrolidine (3c3):

38.0 mg, 23% yield, ***cis:trans*** (71:29). Colorless oil. TLC (hexane): R_f = 0.74.***cis***: ^{1}H NMR (400 MHz, $CDCl_3$) δ 7.43-7.39 (m, 2H), 6.30 (dd, J = 31.5, 9.0 Hz, 2H), 3.49 - 3.44 (m, 2H), 2.06-1.93 (m, 2H), 1.86-1.65 (m, 4H), 1.36-1.27 (m, 2H), 0.92 (t, J = 7.5 Hz, 6H); ^{13}C NMR (101 MHz, $CDCl_3$) δ 147.0, 137.5, 114.3, 75.6, 61.9, 29.2, 27.5, 10.6. ***trans***: ^{1}H NMR (400 MHz, $CDCl_3$) δ 7.43-7.39 (m, 2H), 6.30 (dd, J = 31.5, 9.0 Hz, 2H), 3.61 - 3.57 (m, 2H), 2.06-1.93 (m, 2H), 1.86-1.65 (m, 4H), 1.36-1.27 (m, 2H), 0.87 (t, J = 7.4 Hz, 6H); ^{13}C NMR (101 MHz, $CDCl_3$) δ 144.7, 137.6, 115.9, 75.0, 59.4, 27.2, 23.6, 11.0. IR (neat): 2958.44, 2357.72, 1624.93, 1598.07, 1507.23, 1472.39, 1389.16, 1366.53, 821.45, 802.52, 738.23, 705.12. HRMS (ESI, m/z): $[M+H]^+$ Calcd. for $C_{14}H_{20}IN$, 330.0719; found, 330.0725.

2, 5-Diethyl-1-(4-iodophenyl)-1H-pyrrole (3c3'):

72.9 mg, 50% yield, white oil. TLC (hexane): R_f = 0.69. ^{1}H NMR (400 MHz, $CDCl_3$) δ 7.78 (d, J = 6.6 Hz, 2H), 6.97 (d, J = 6.8 Hz, 2H), 5.95 (s, 2H), 2.33 (q, J = 7.5 Hz, 4H), 1.09 (t, J = 7.5 Hz, 6H); ^{13}C NMR (101 MHz, $CDCl_3$) δ 138.8, 138.4, 135.4, 130.5, 104.2, 93.2, 20.3, 13.2. IR (neat): 2966.71, 2360.75, 1652.60, 1585.19, 1488.68, 1003.89, 830.64, 733.70. HRMS (ESI, m/z): $[M+H]^+$ Calcd. for $C_{14}H_{16}IN$, 326.0406; found, 326.0418.

E. References

[1] a) Carla, B; Francesco, M; Diego S. *Tetrahedron Lett.* **1994,** 50, 4709.; b) Barluenga, J.; Najera, C.; Yus, M. *J. Heterocyclic Chem.* **1981**, 18, 1297.; c) Menuel, S.;

Bertaut, E.; Monflier, E.; Hapiot, F. *Dalton Trans* **2015**, 44, 13504.

[2] Takeda, T.; Saito, J.; Tsubouchi, A. *Tetrahedron Lett.* **2003**, 44, 5571.

F. NMR Spectra

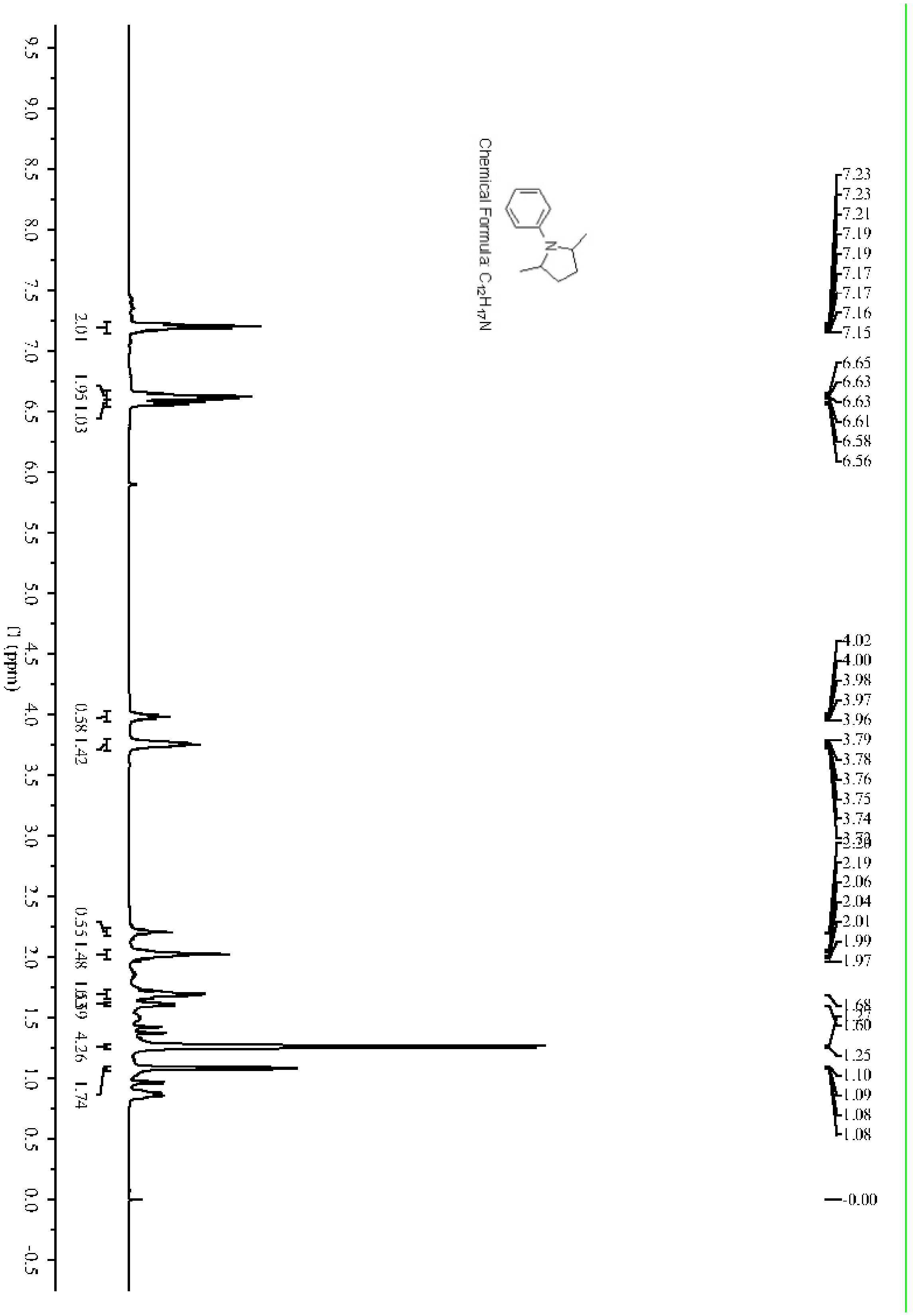

^{1}H NMR (400 MHz, $CDCl_3$) spectrum of **3a1**

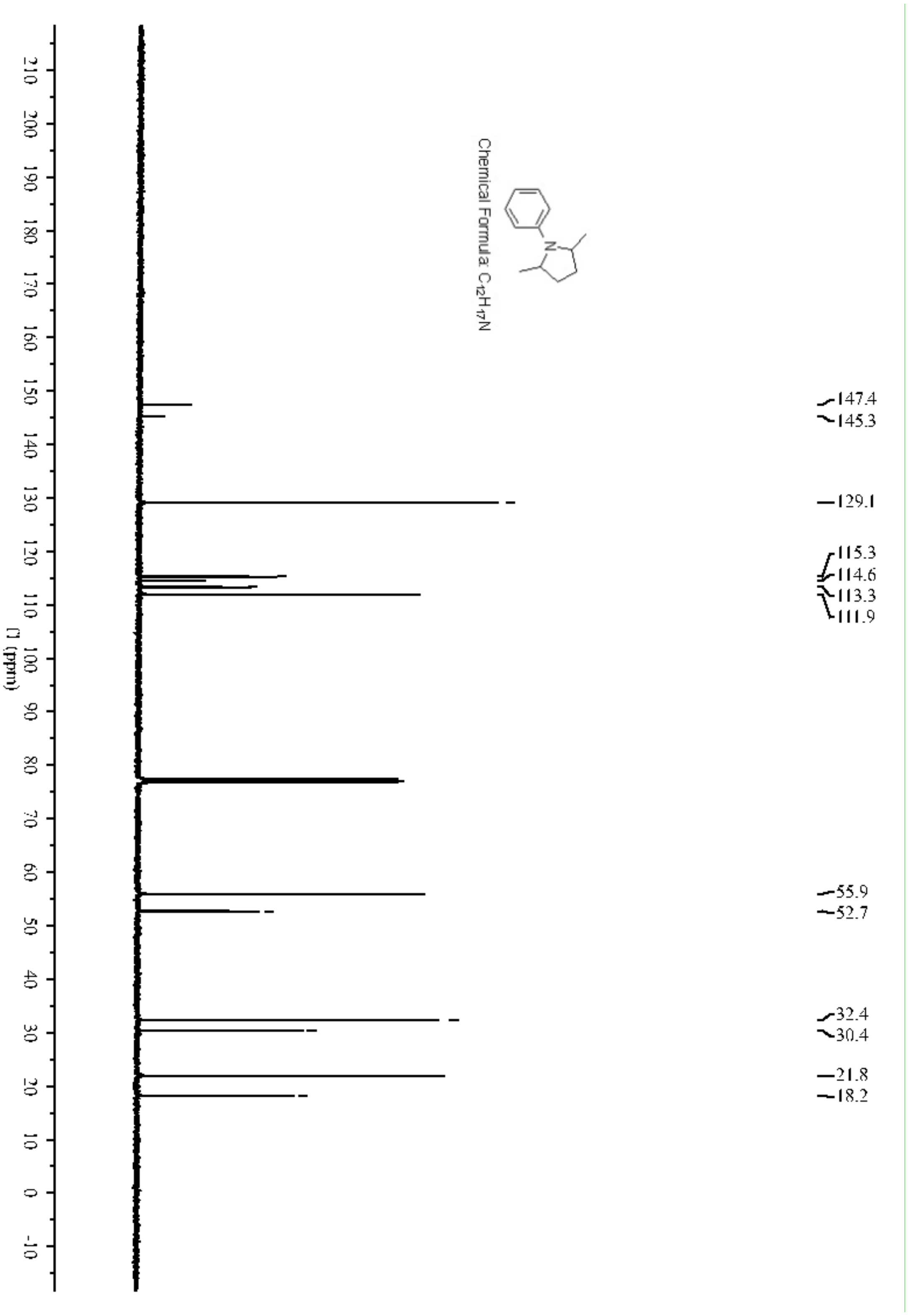

^{13}C NMR (101 MHz, $CDCl_3$) spectrum of **3a1**

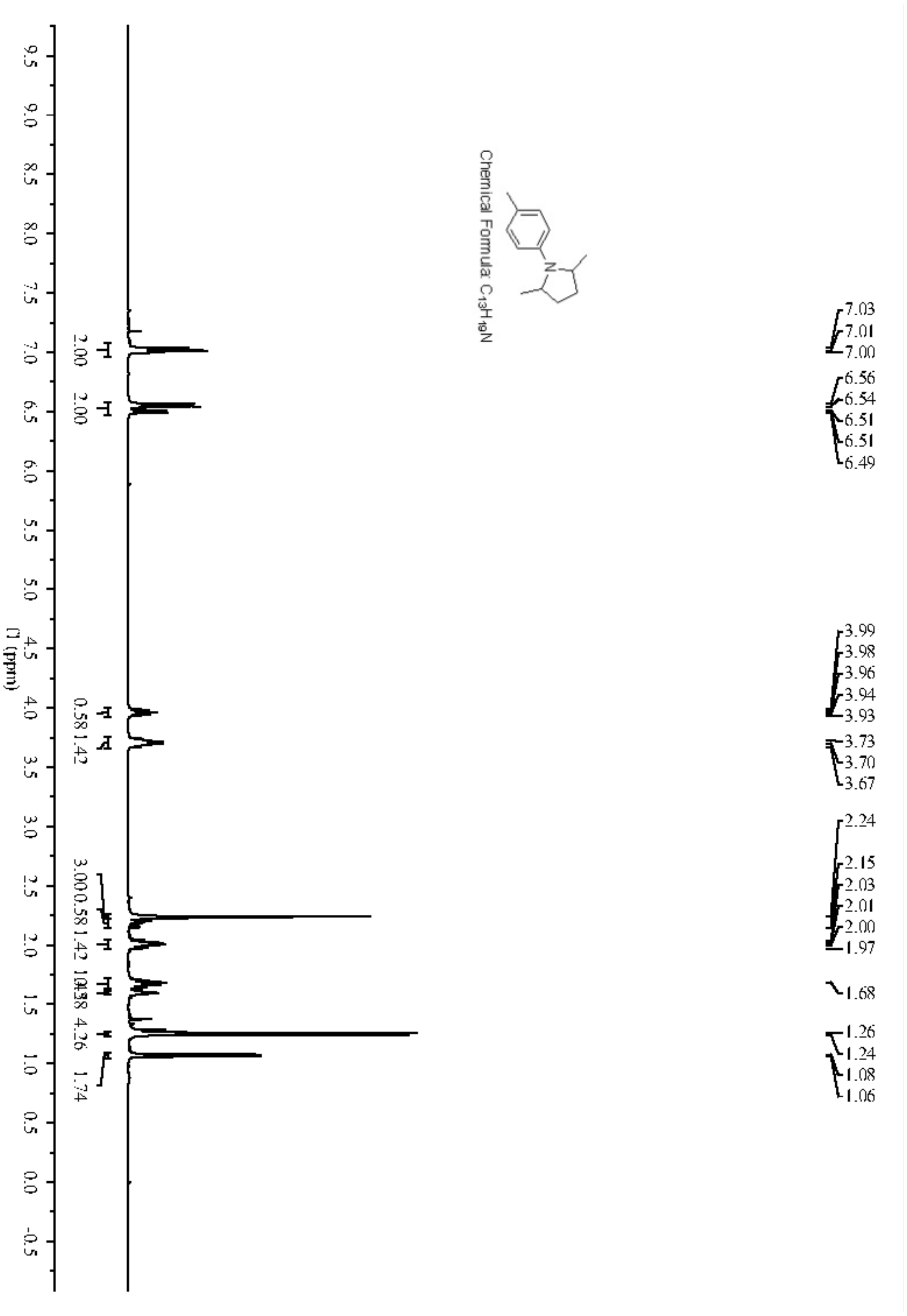

^{1}H NMR (400 MHz, CDCl₃) spectrum of **3a2**

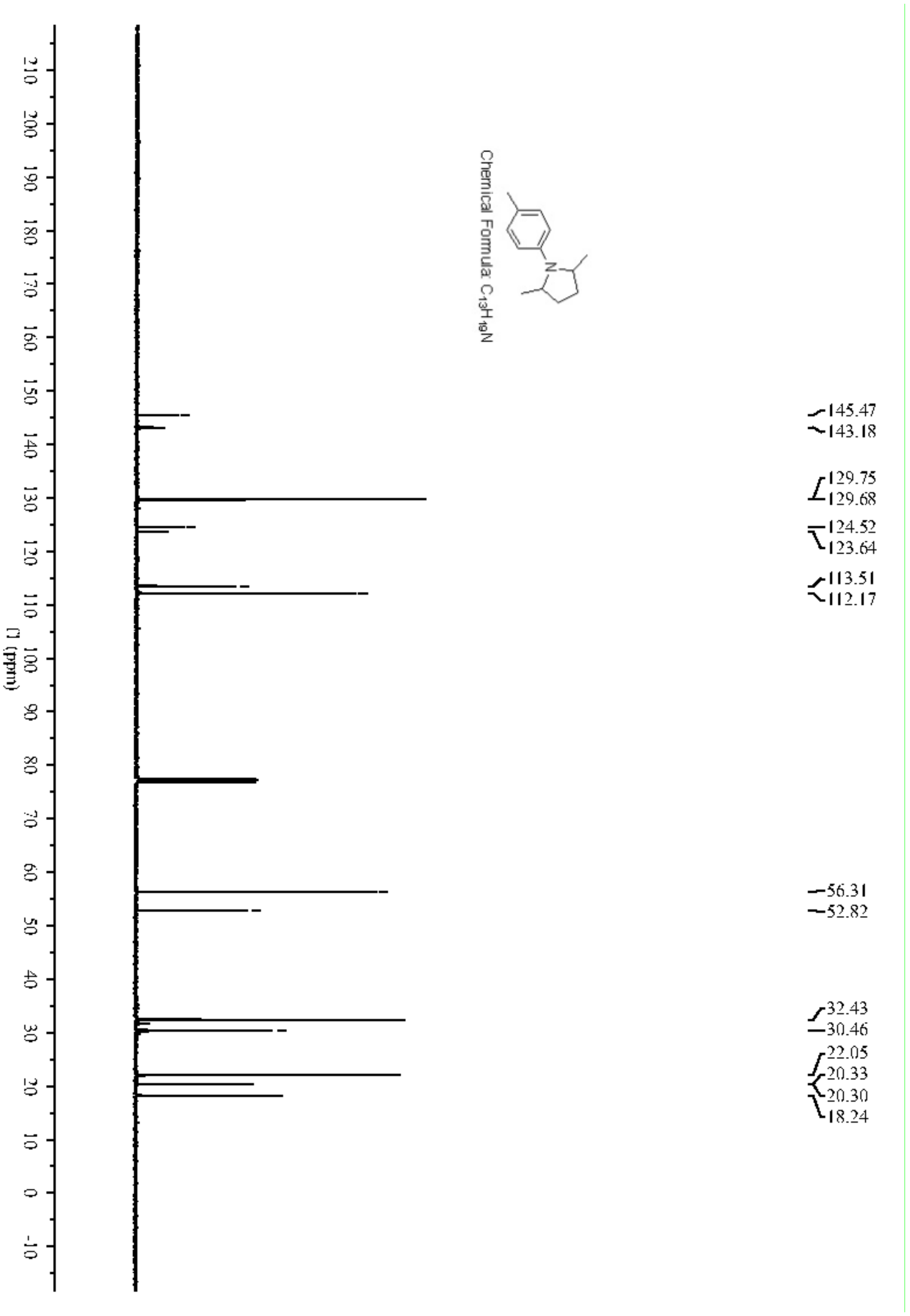

^{13}C NMR (101 MHz, $CDCl_3$) spectrum of **3a2**

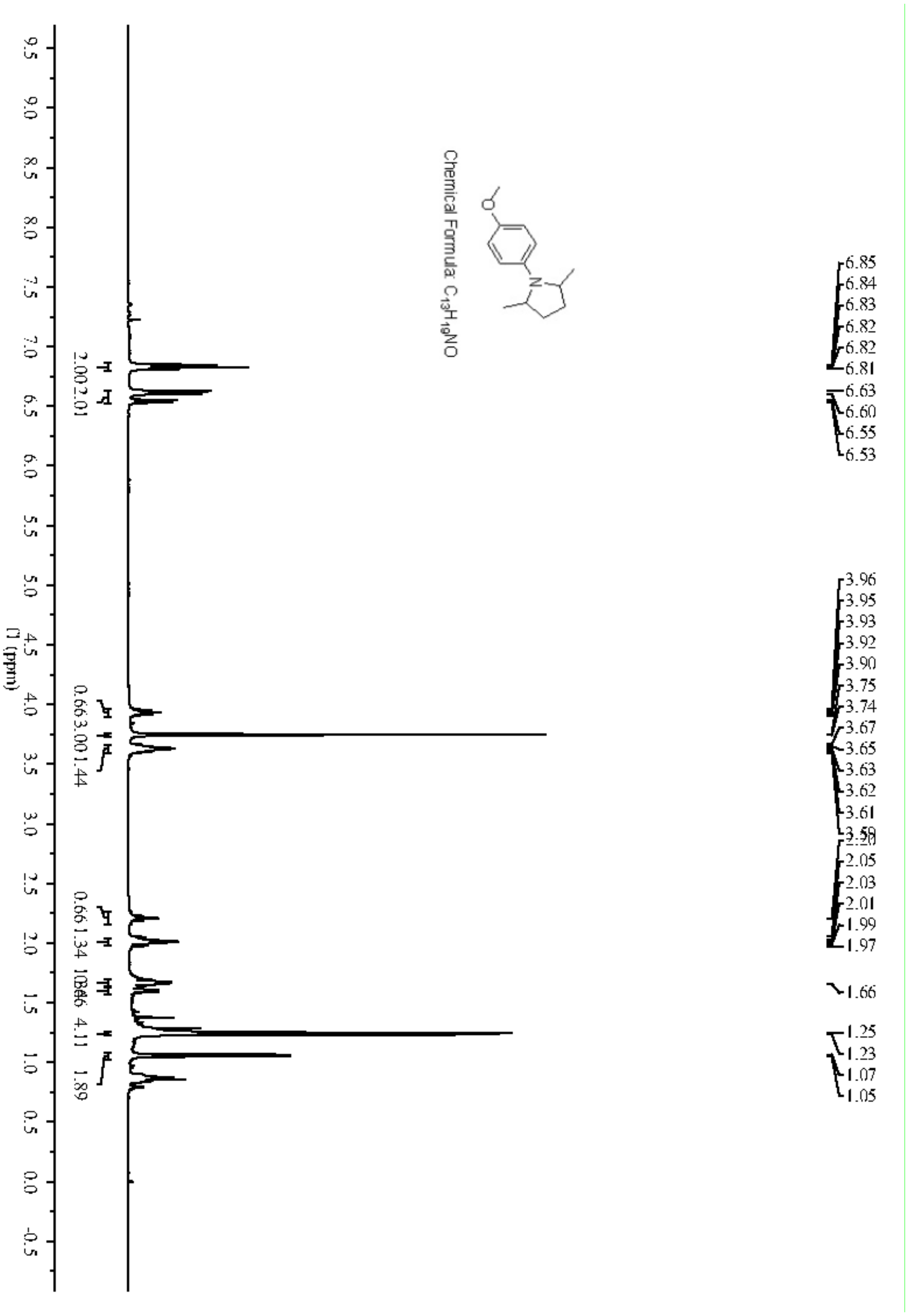

^{1}H NMR (400 MHz, $CDCl_3$) spectrum of **3a3**

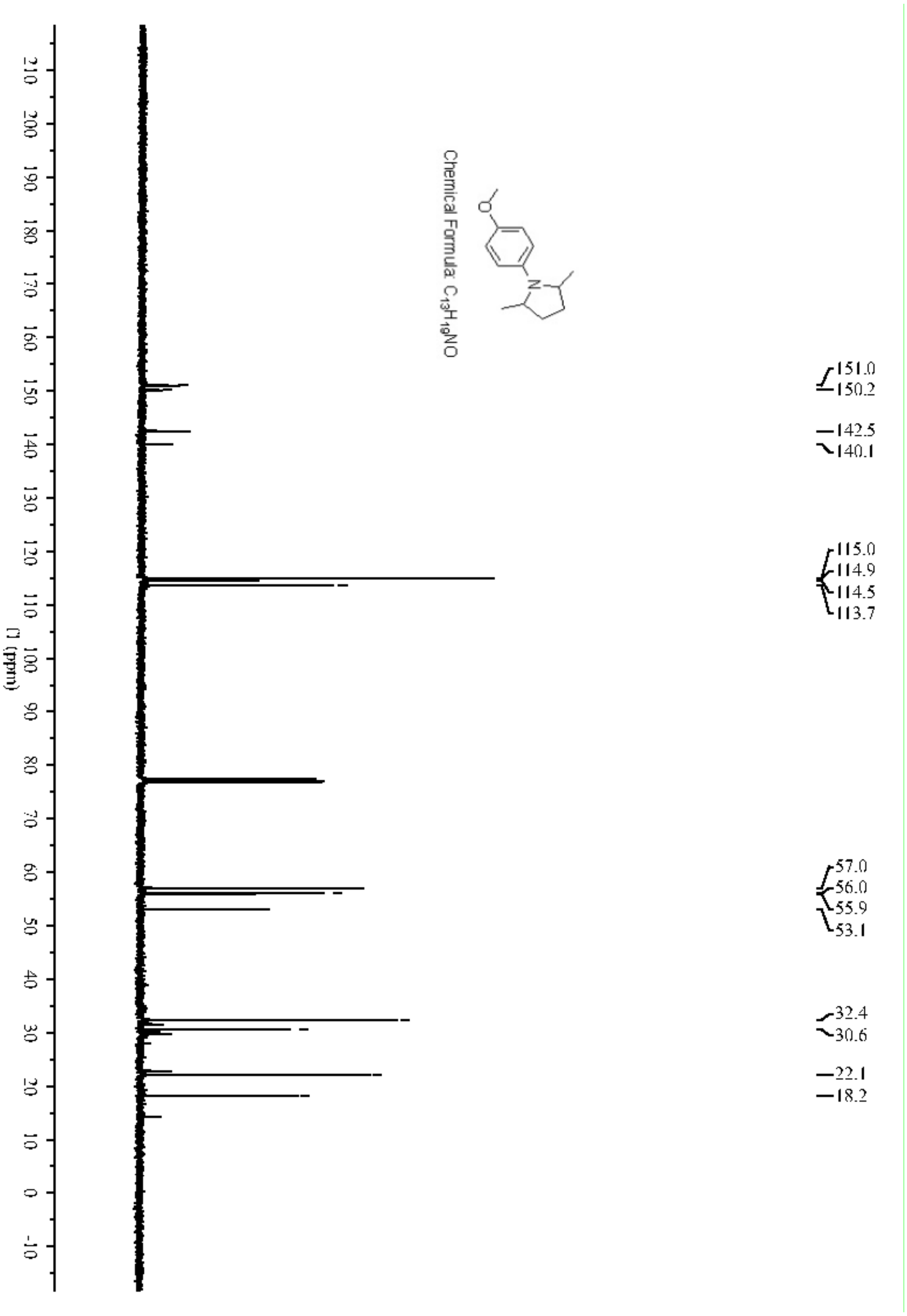

^{13}C NMR (101 MHz, $CDCl_3$) spectrum of **3a3**

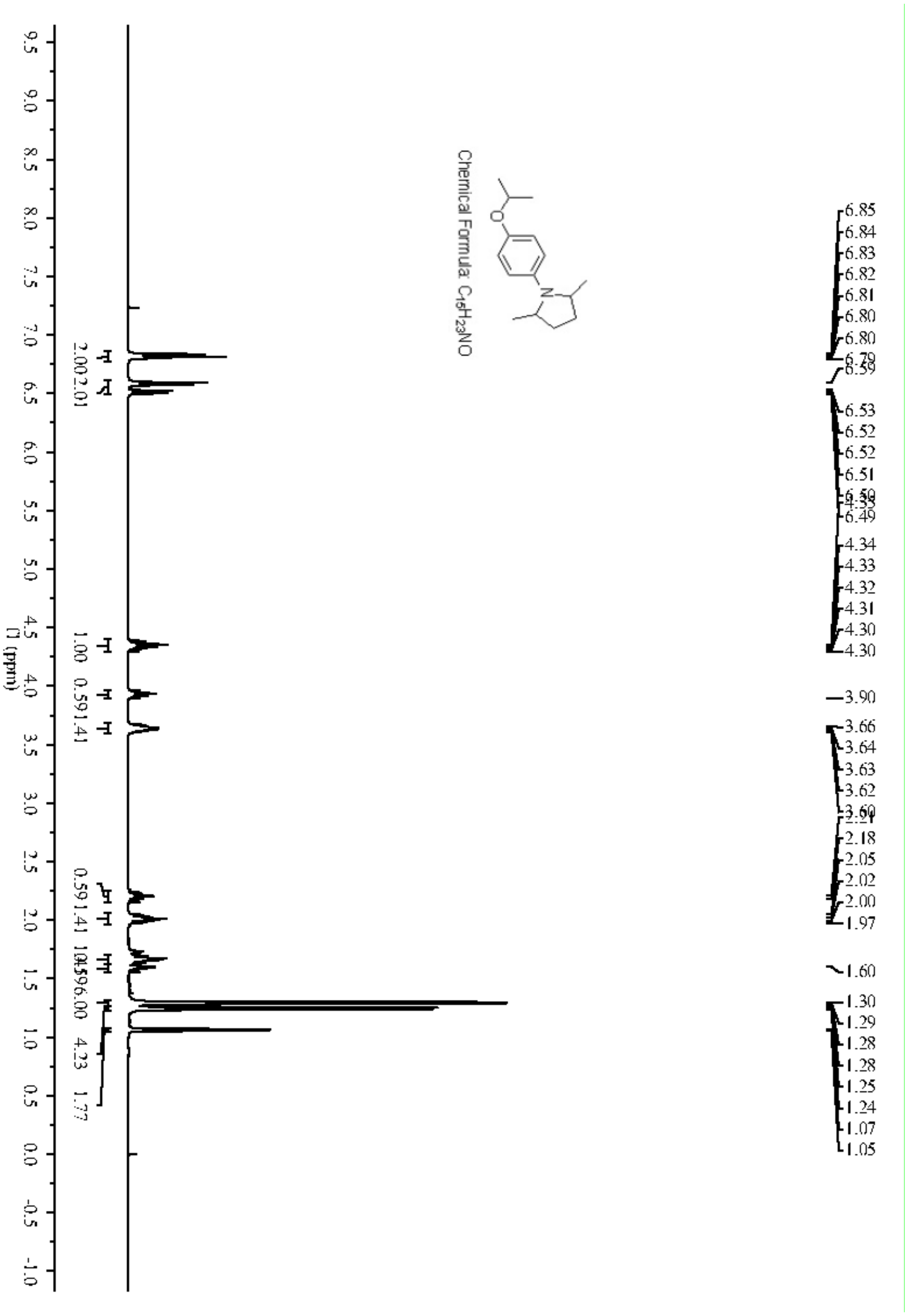

^{1}H NMR (400 MHz, $CDCl_3$) spectrum of **3a4**

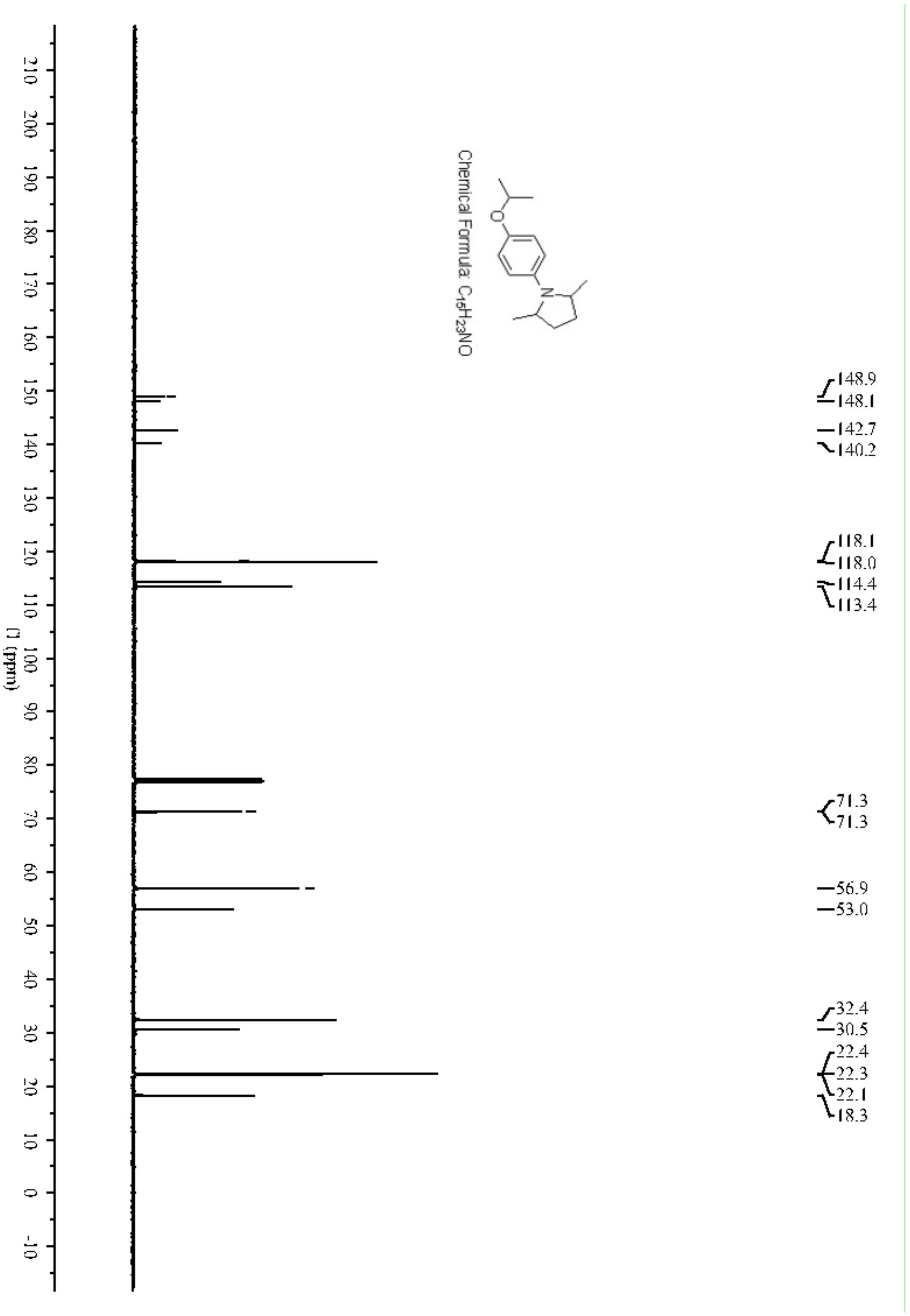

^{13}C NMR (101 MHz, $CDCl_3$) spectrum of **3a4**

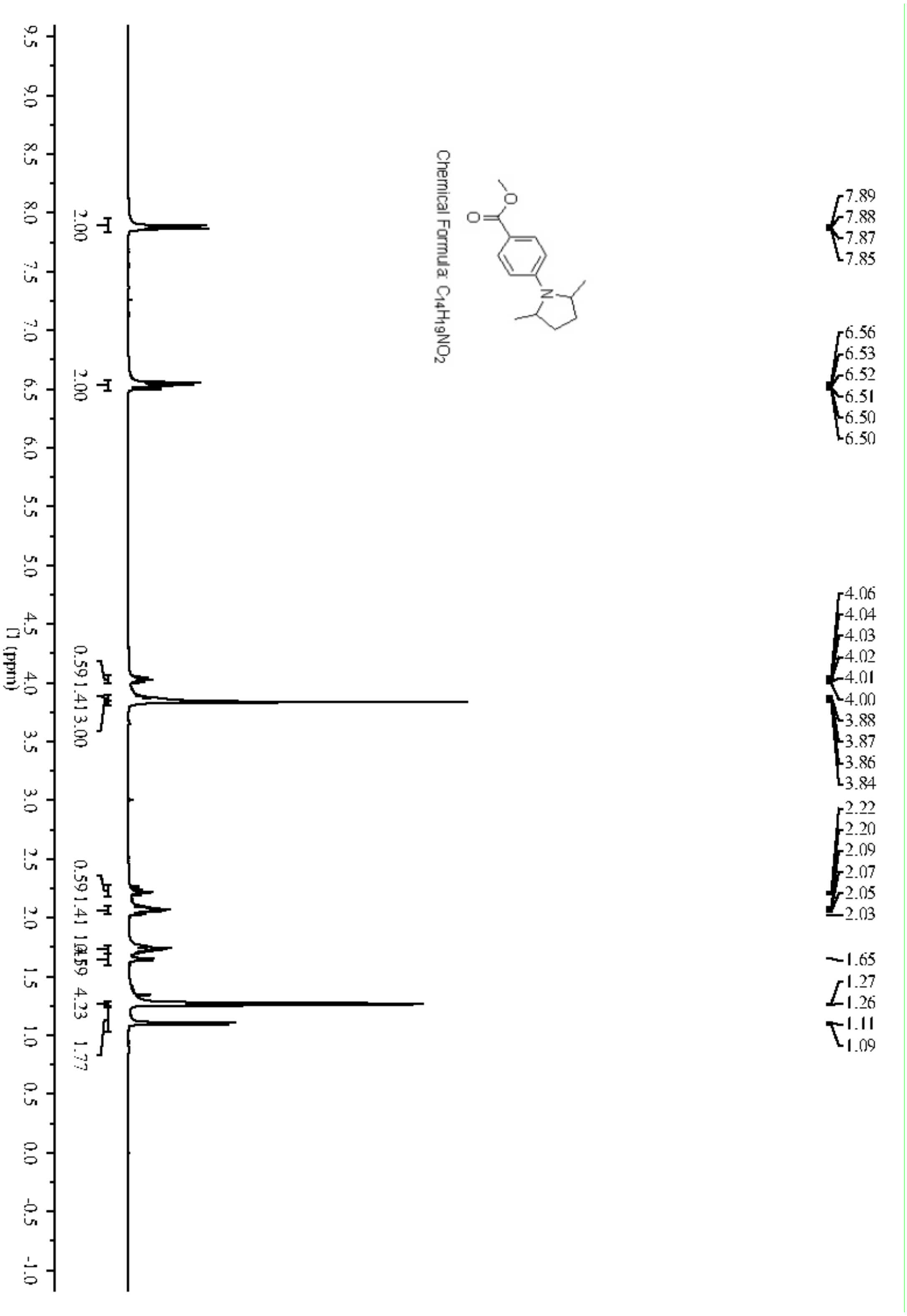

^{1}H NMR (400 MHz, $CDCl_3$) spectrum of **3a5**

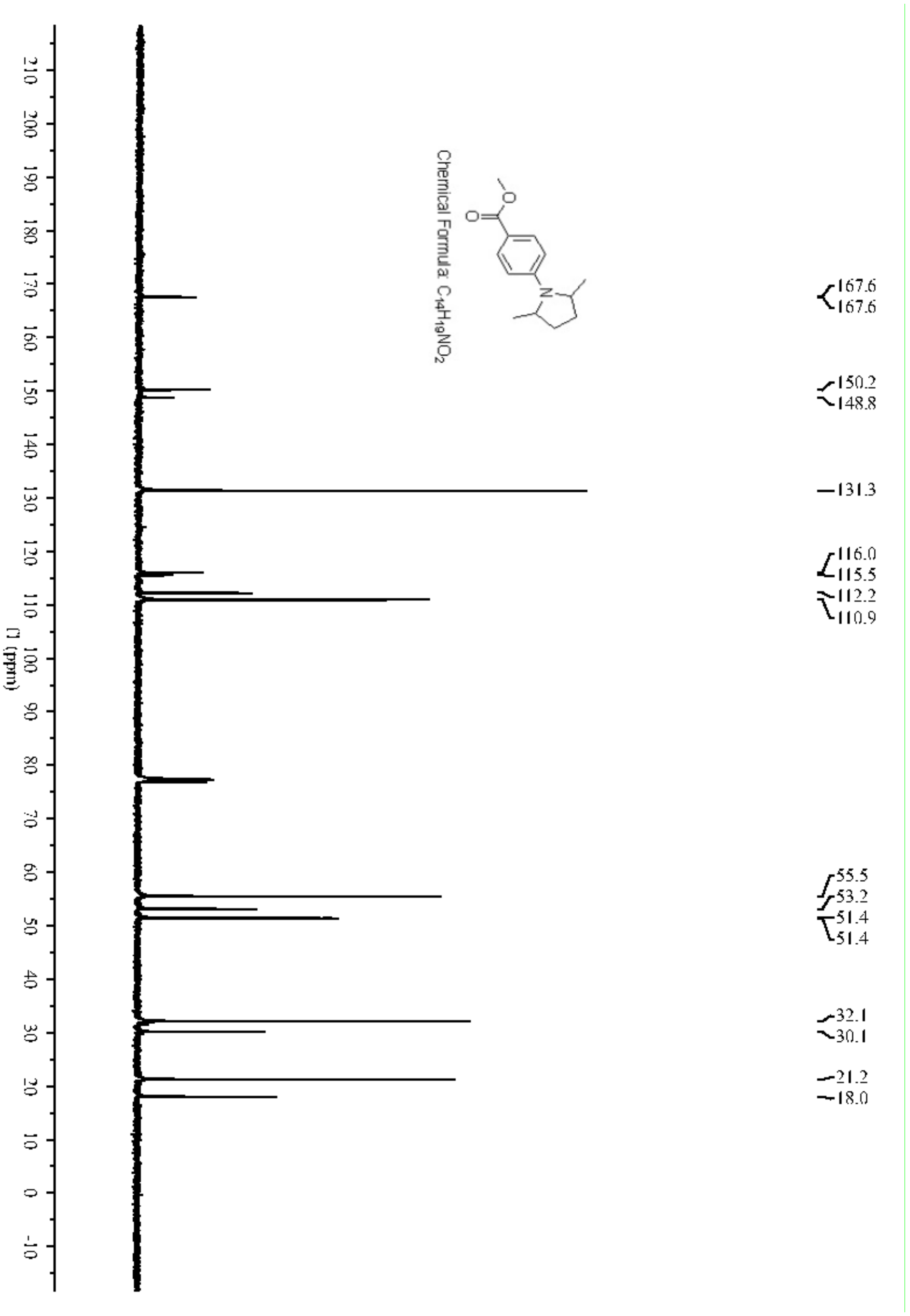

^{13}C NMR (101 MHz, $CDCl_3$) spectrum of **3a5**

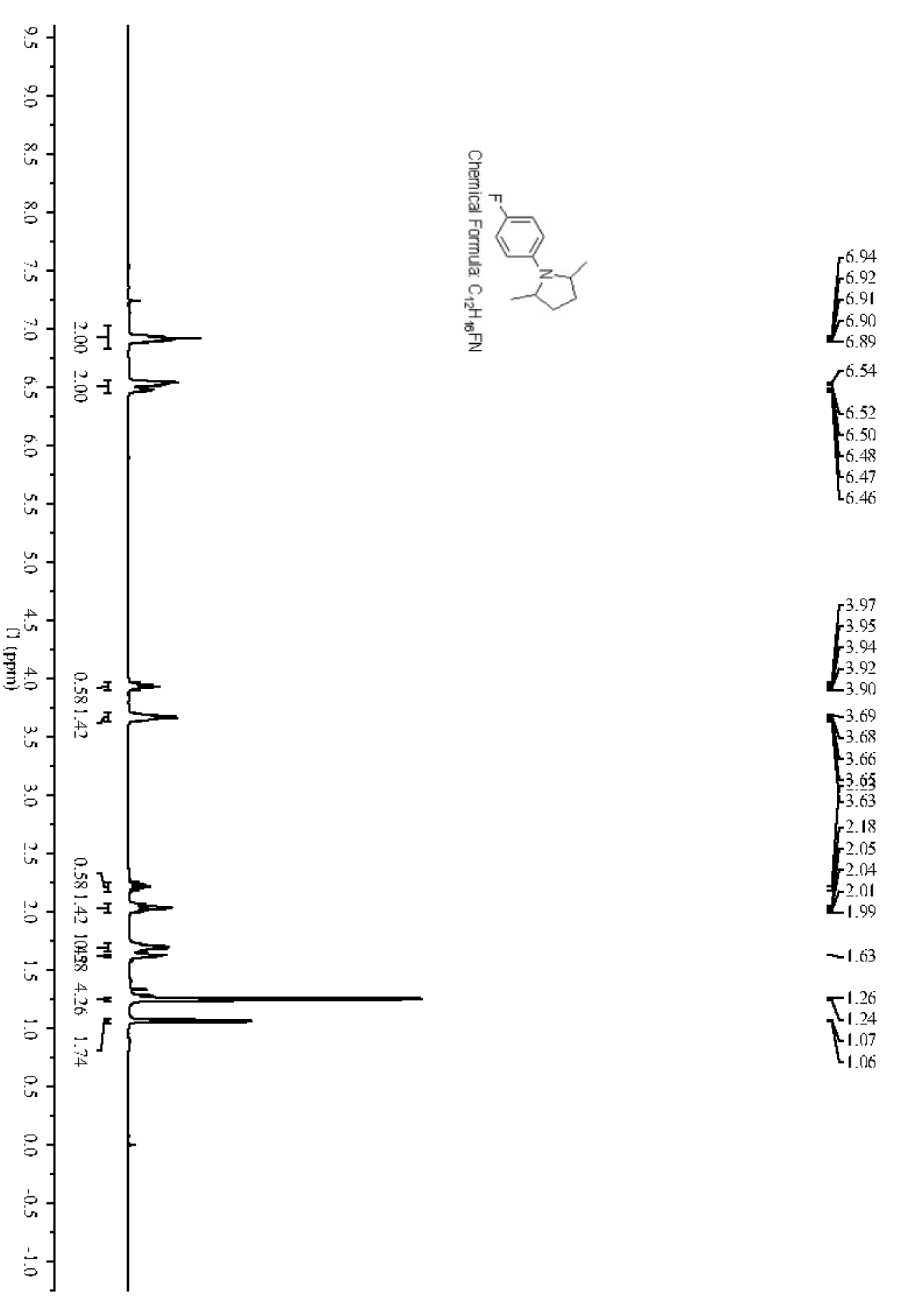

^{1}H NMR (400 MHz, $CDCl_3$) spectrum of **3a6**

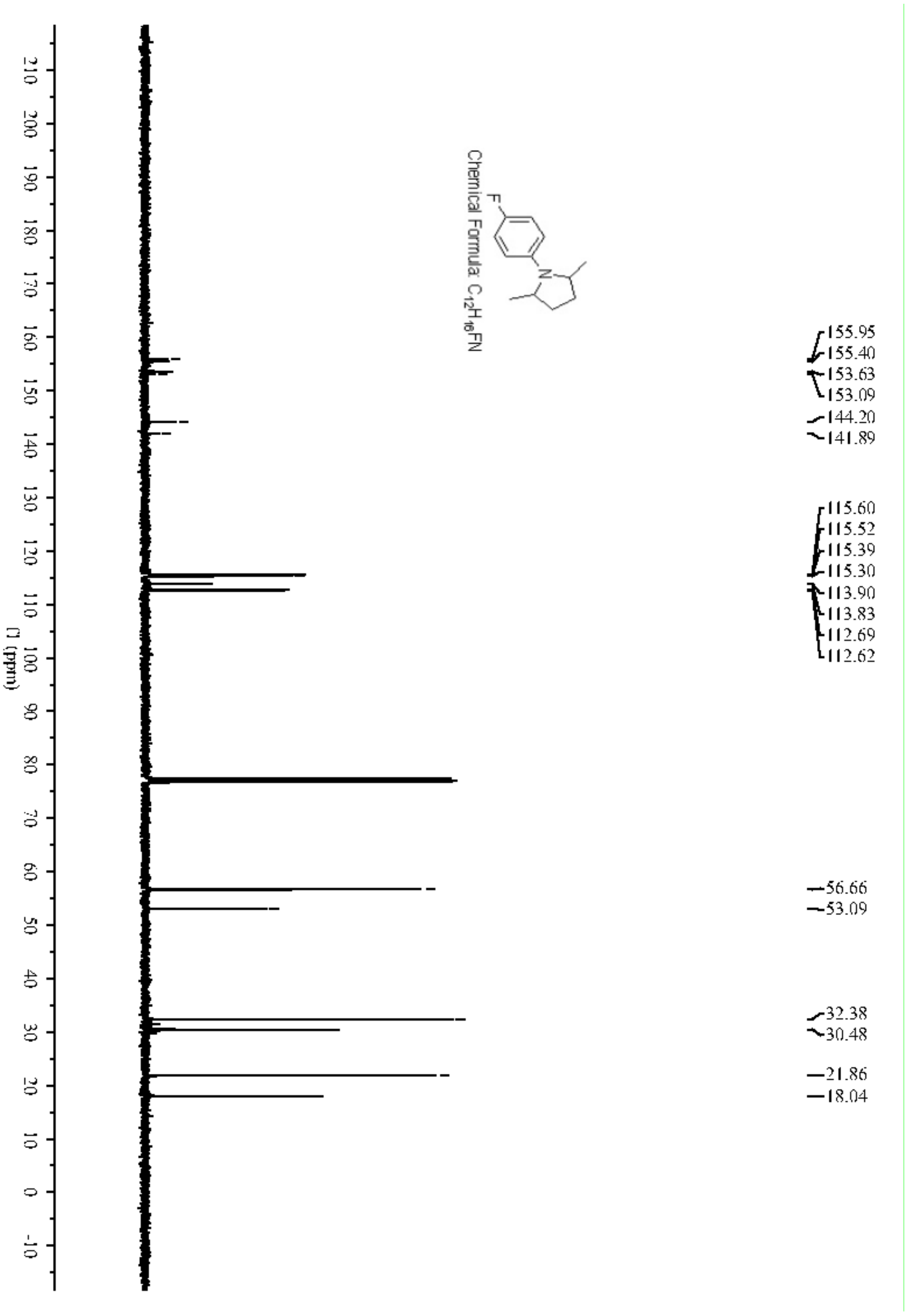

^{13}C NMR (101 MHz, CDCl$_3$) spectrum of **3a6**

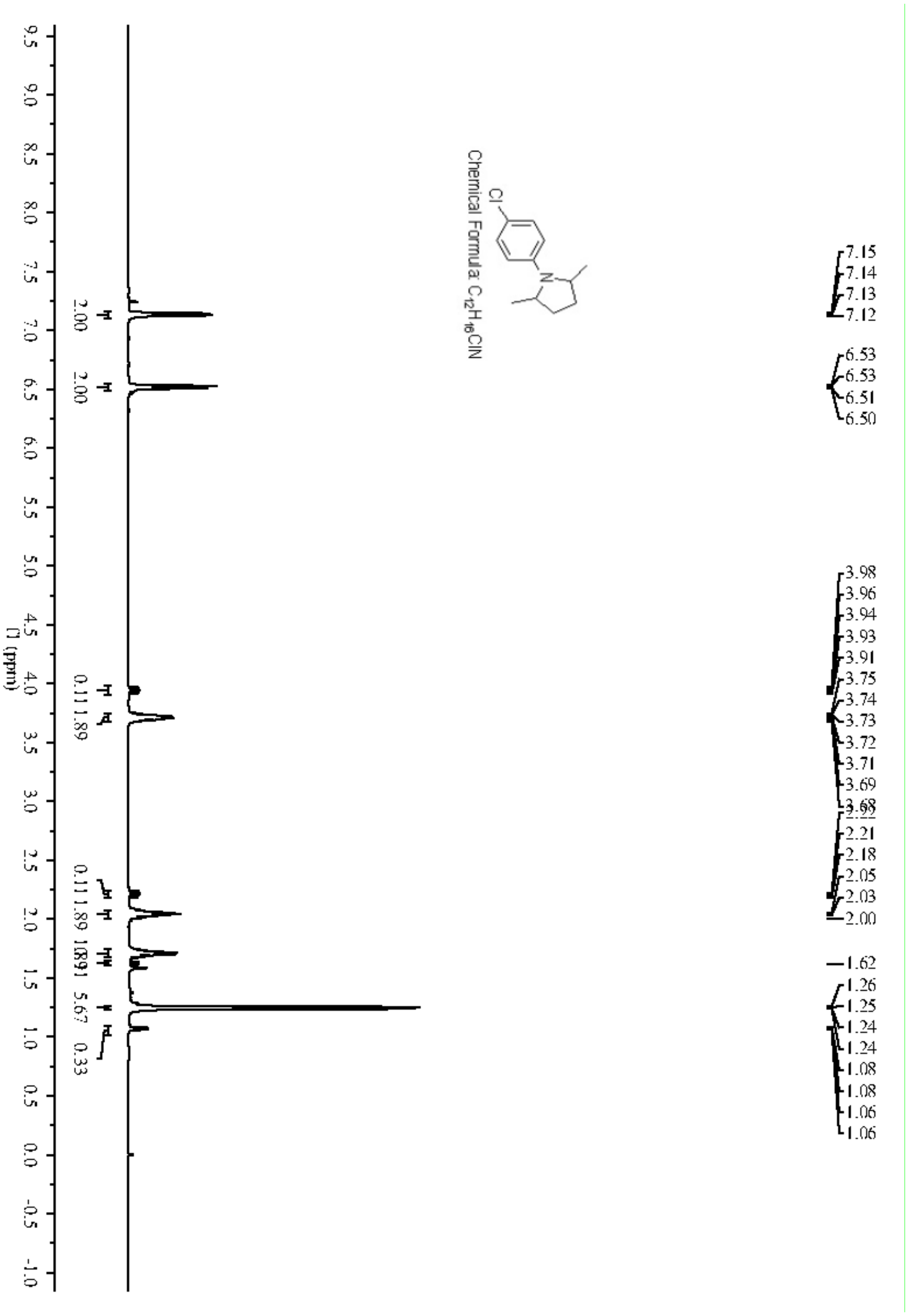

^{1}H NMR (400 MHz, CDCl₃) spectrum of **3a7**

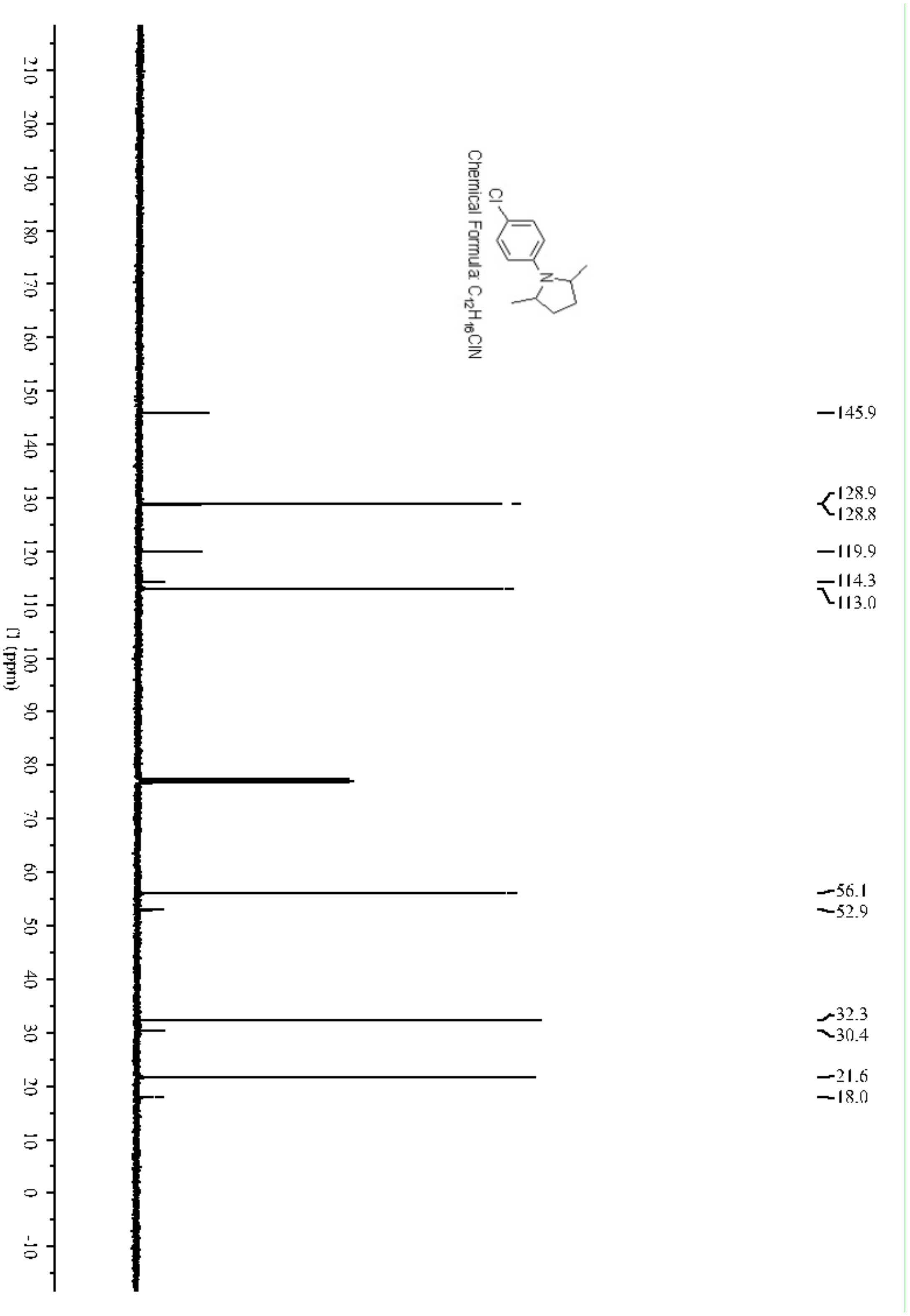

^{13}C NMR (101 MHz, $CDCl_3$) spectrum of **3a7**

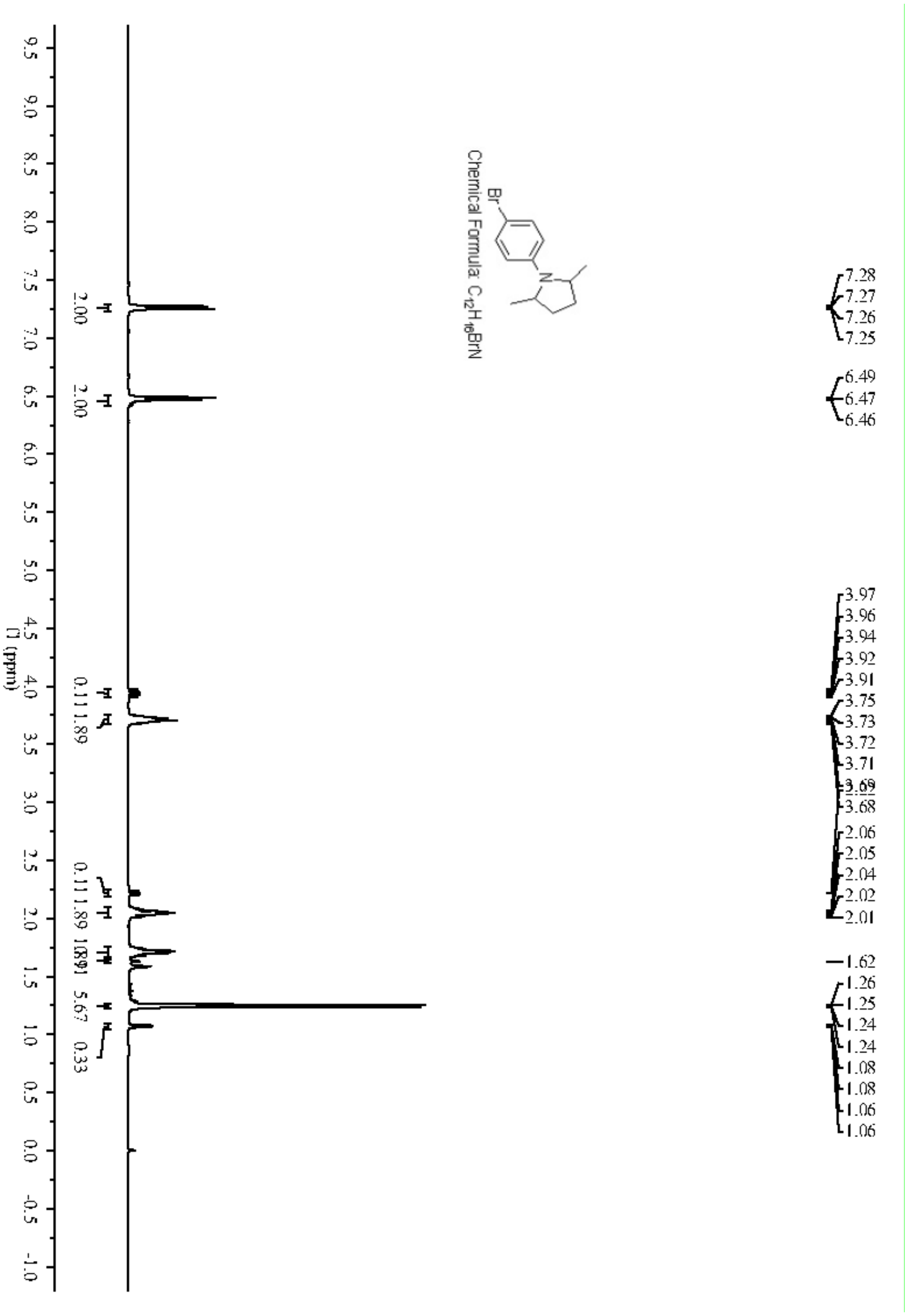

^{1}H NMR (400 MHz, $CDCl_3$) spectrum of **3a8**

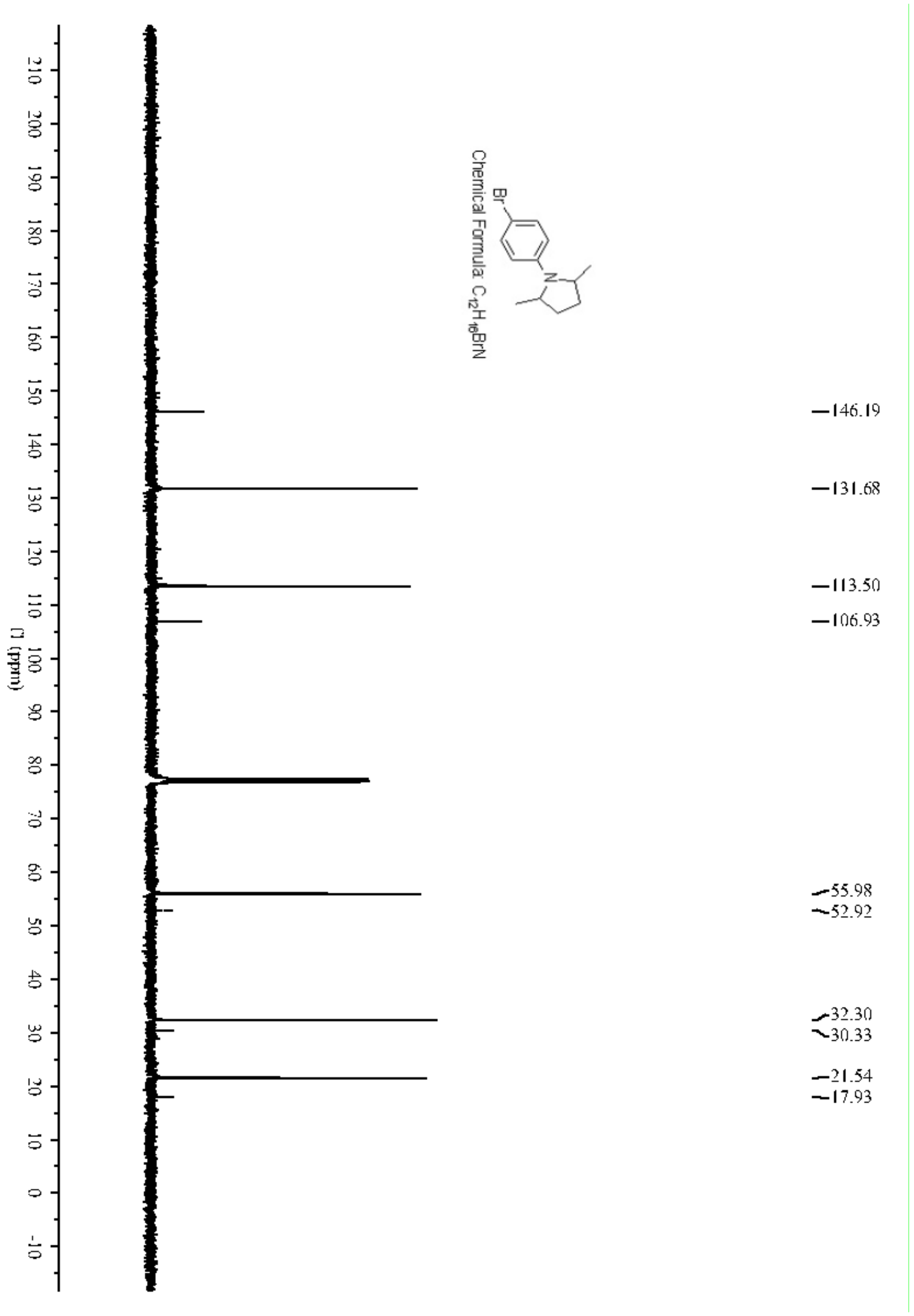

^{13}C NMR (101 MHz, CDCl₃) spectrum of **3a8**

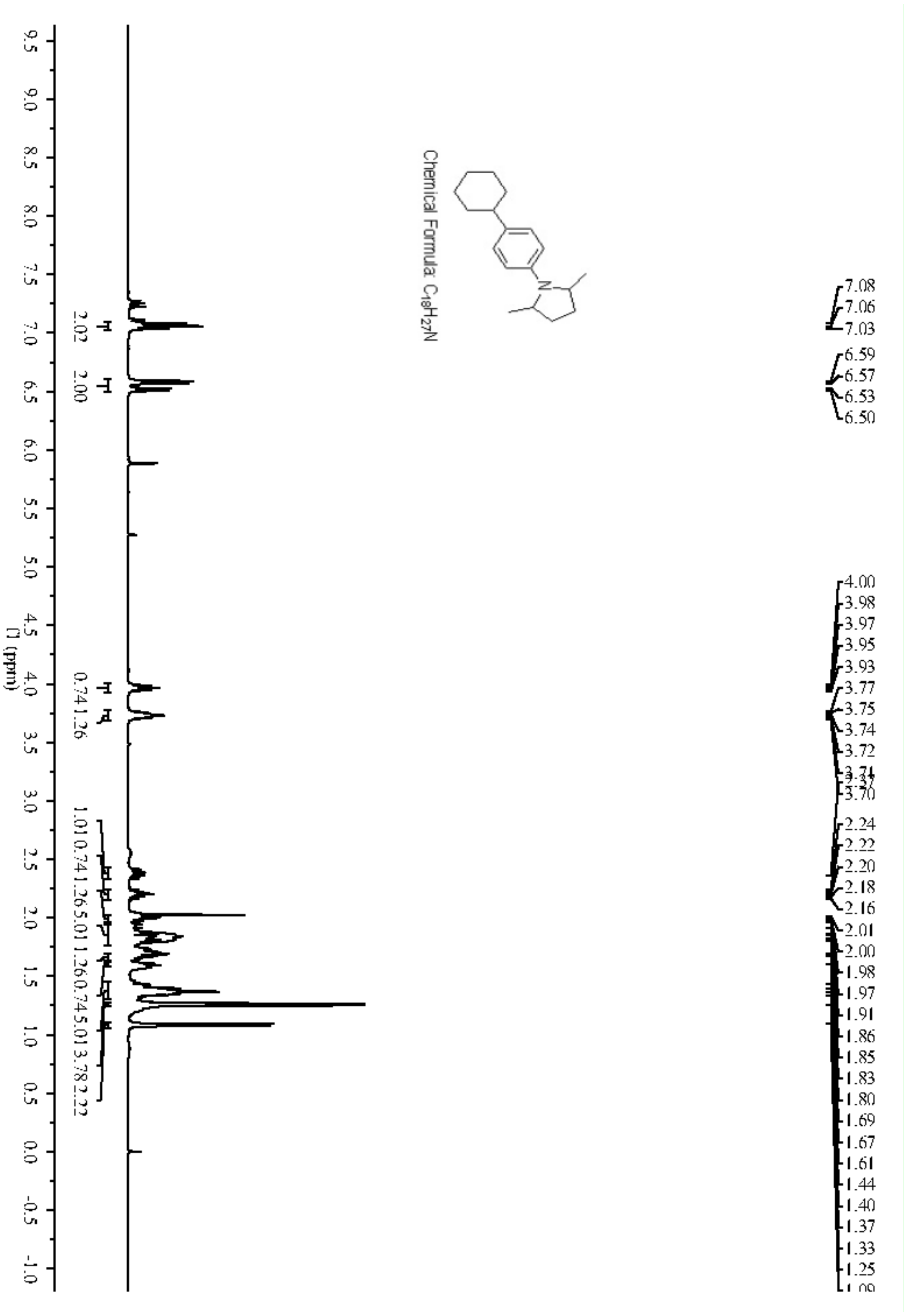

^{1}H NMR (400 MHz, $CDCl_3$) spectrum of **3a9**

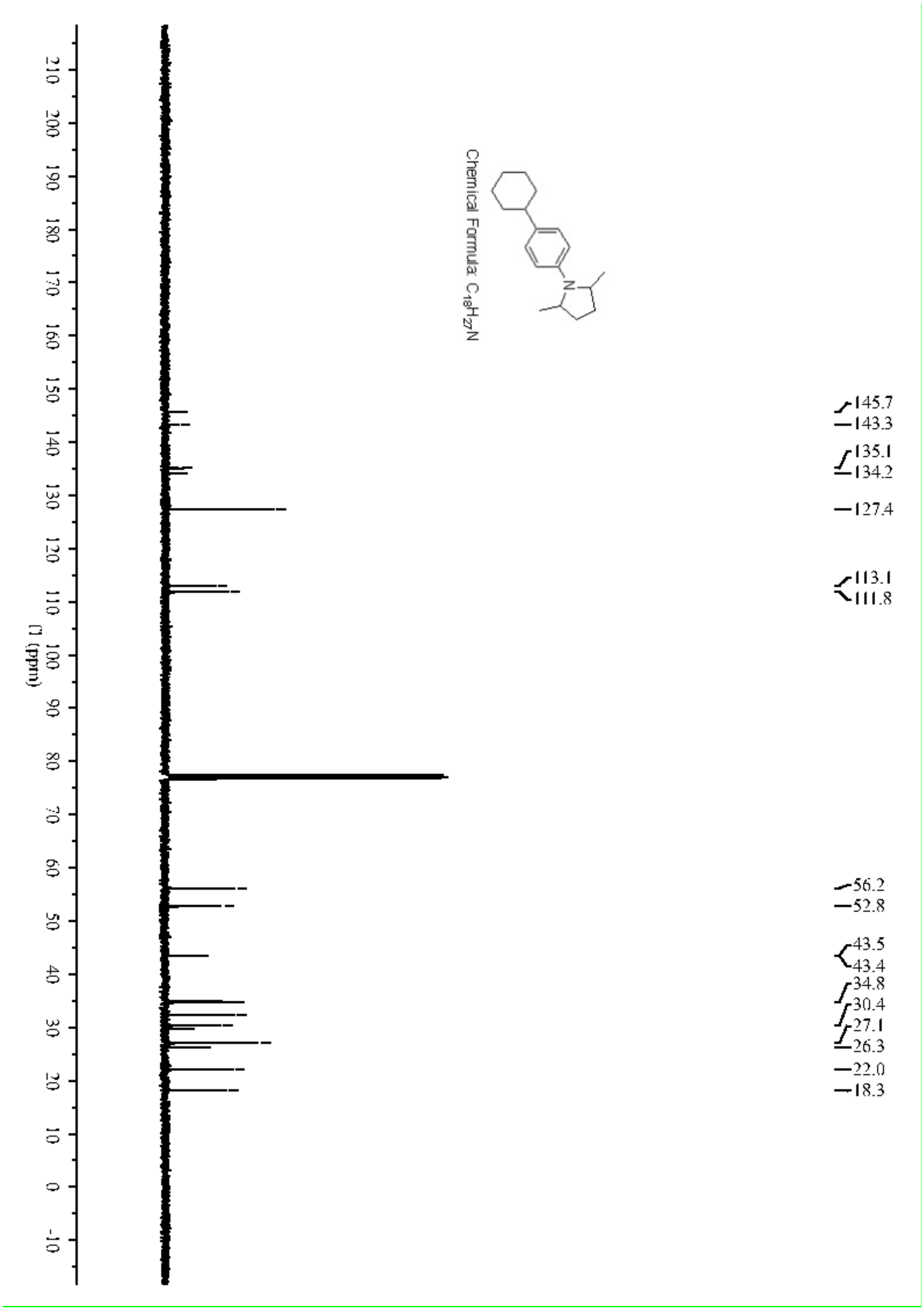

^{13}C NMR (101 MHz, $CDCl_3$) spectrum of **3a9**

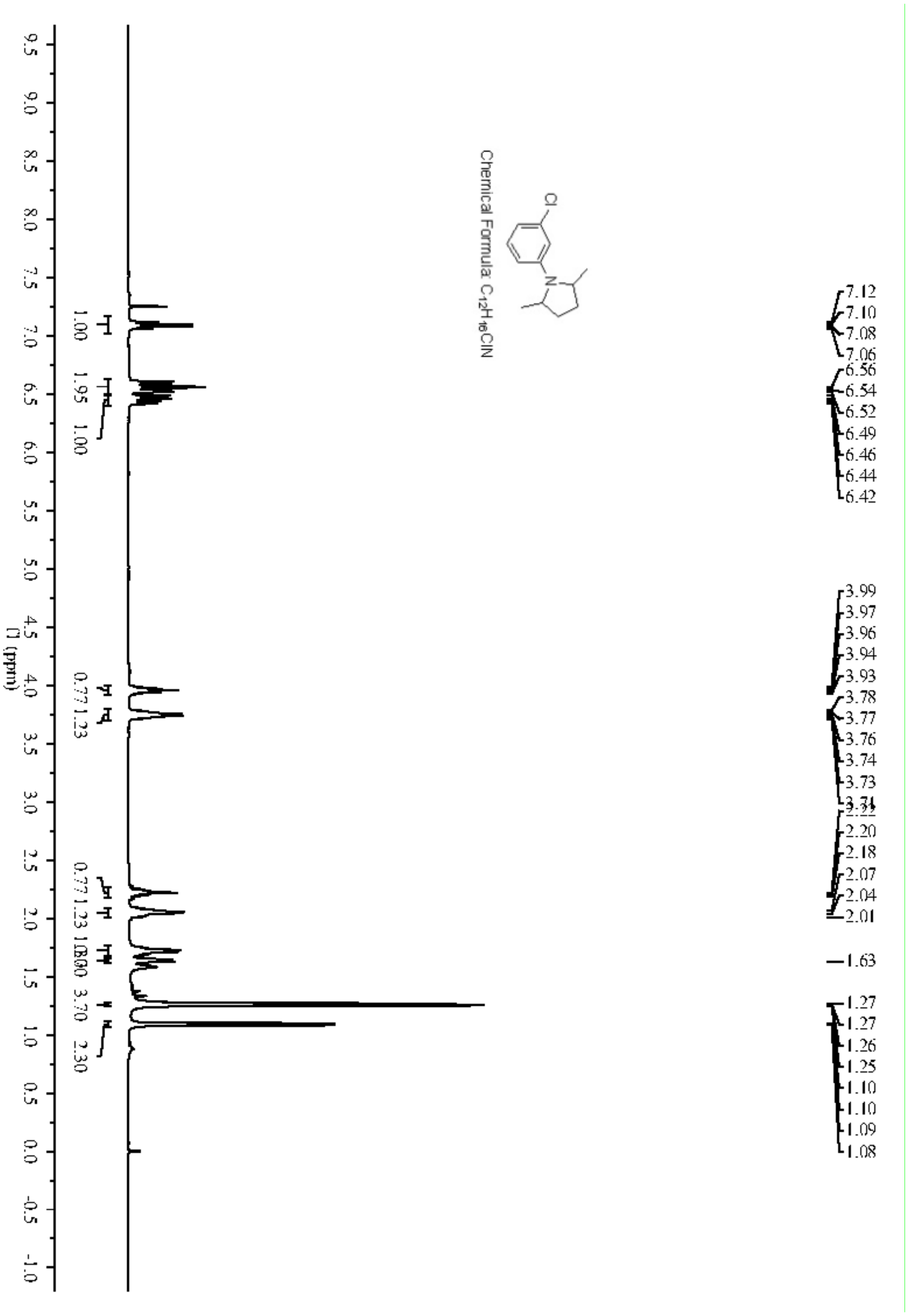

^{1}H NMR (400 MHz, $CDCl_3$) spectrum of **3a10**

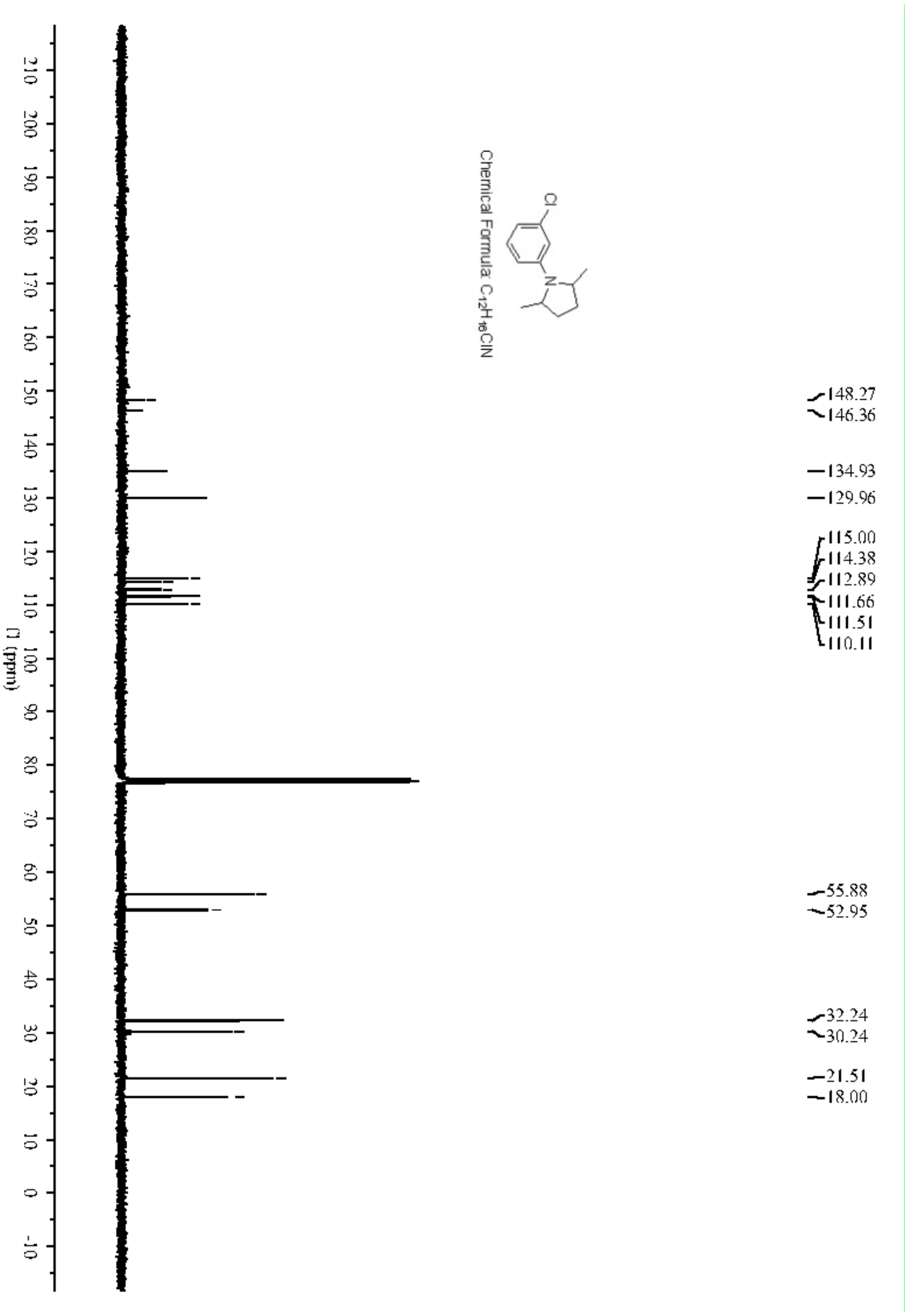

^{13}C NMR (101 MHz, $CDCl_3$) spectrum of **3a10**

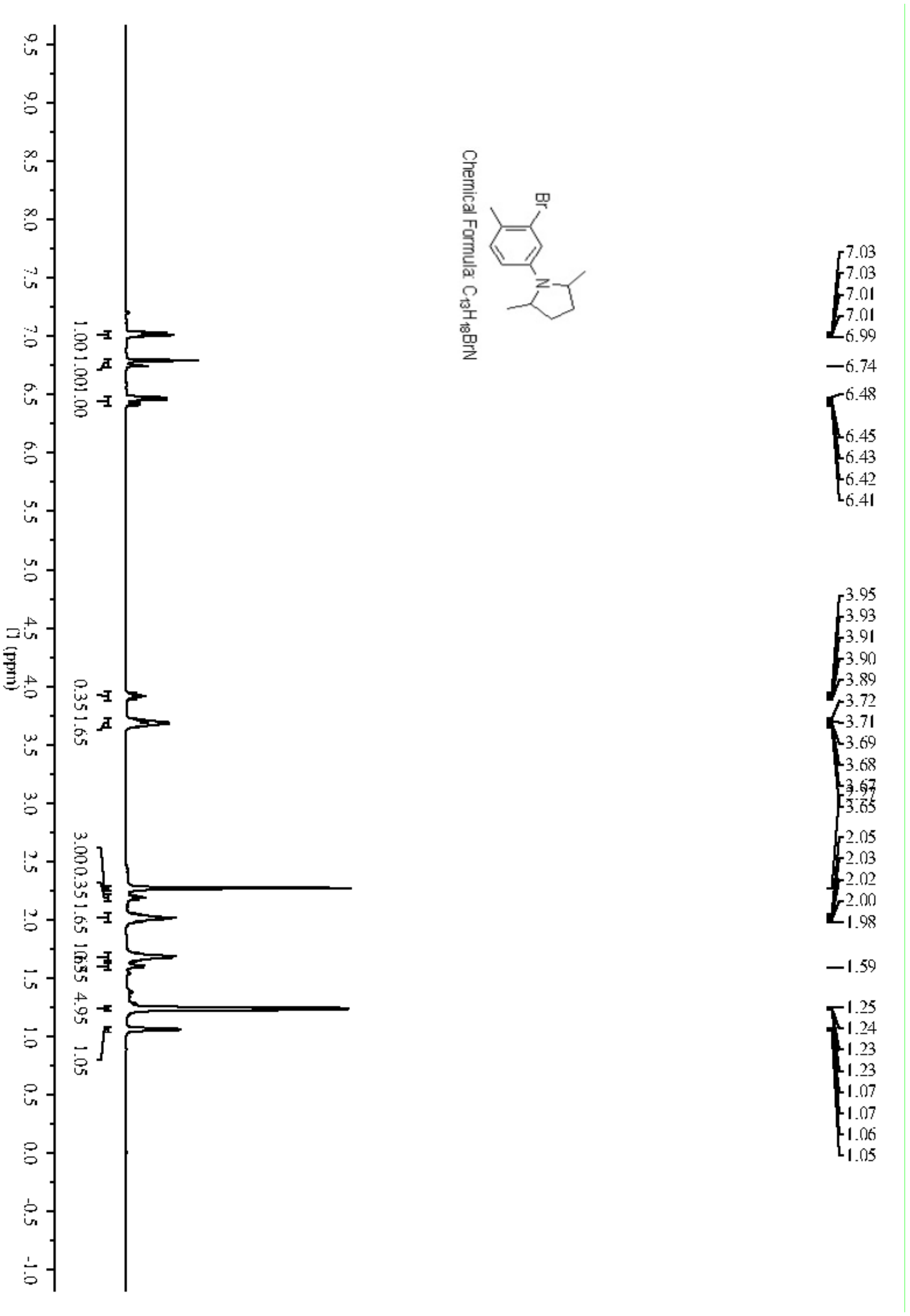

^{1}H NMR (400 MHz, $CDCl_3$) spectrum of **3a11**

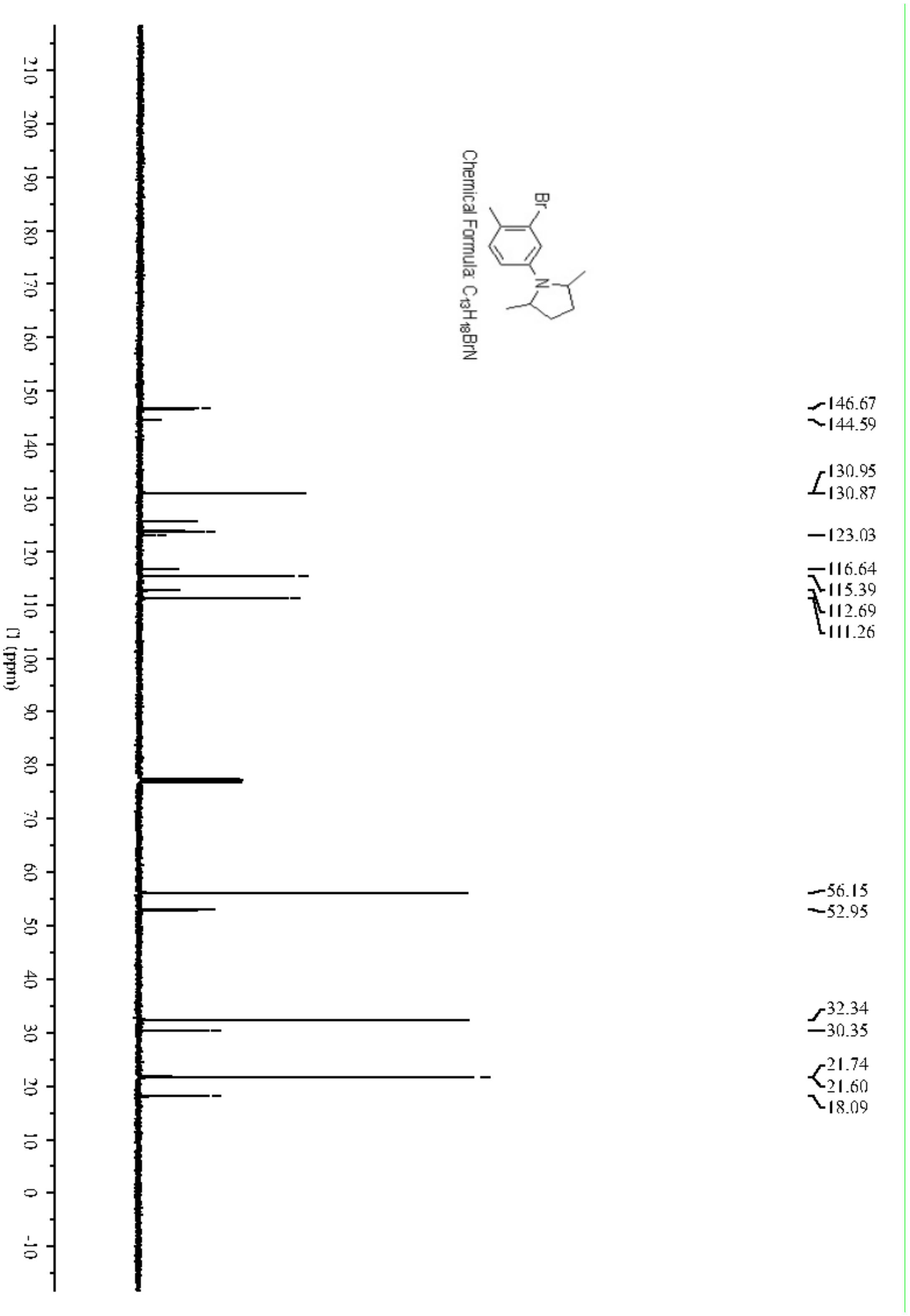

^{13}C NMR (101 MHz, $CDCl_3$) spectrum of **3a11**

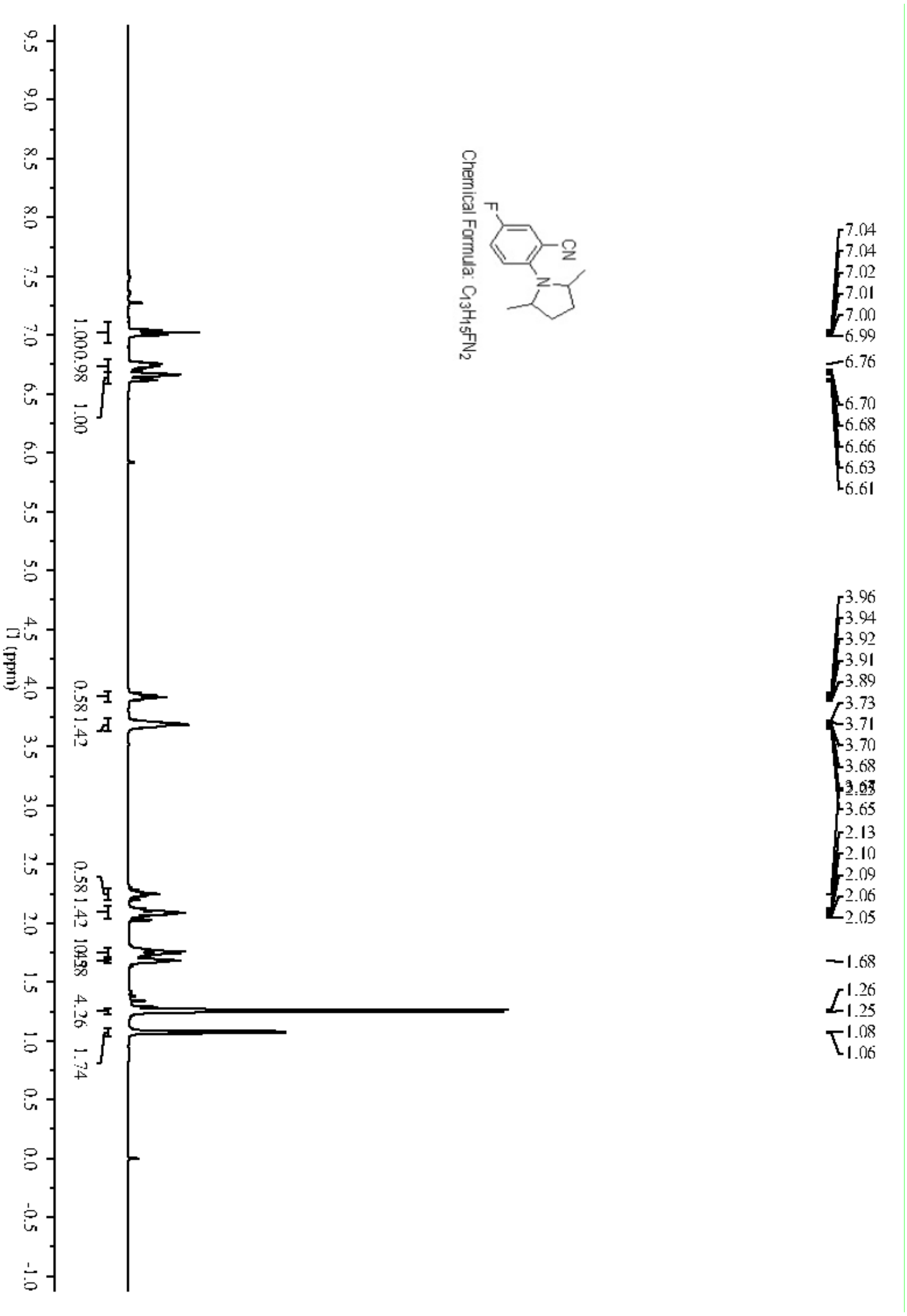

^{1}H NMR (400 MHz, $CDCl_3$) spectrum of **3a12**

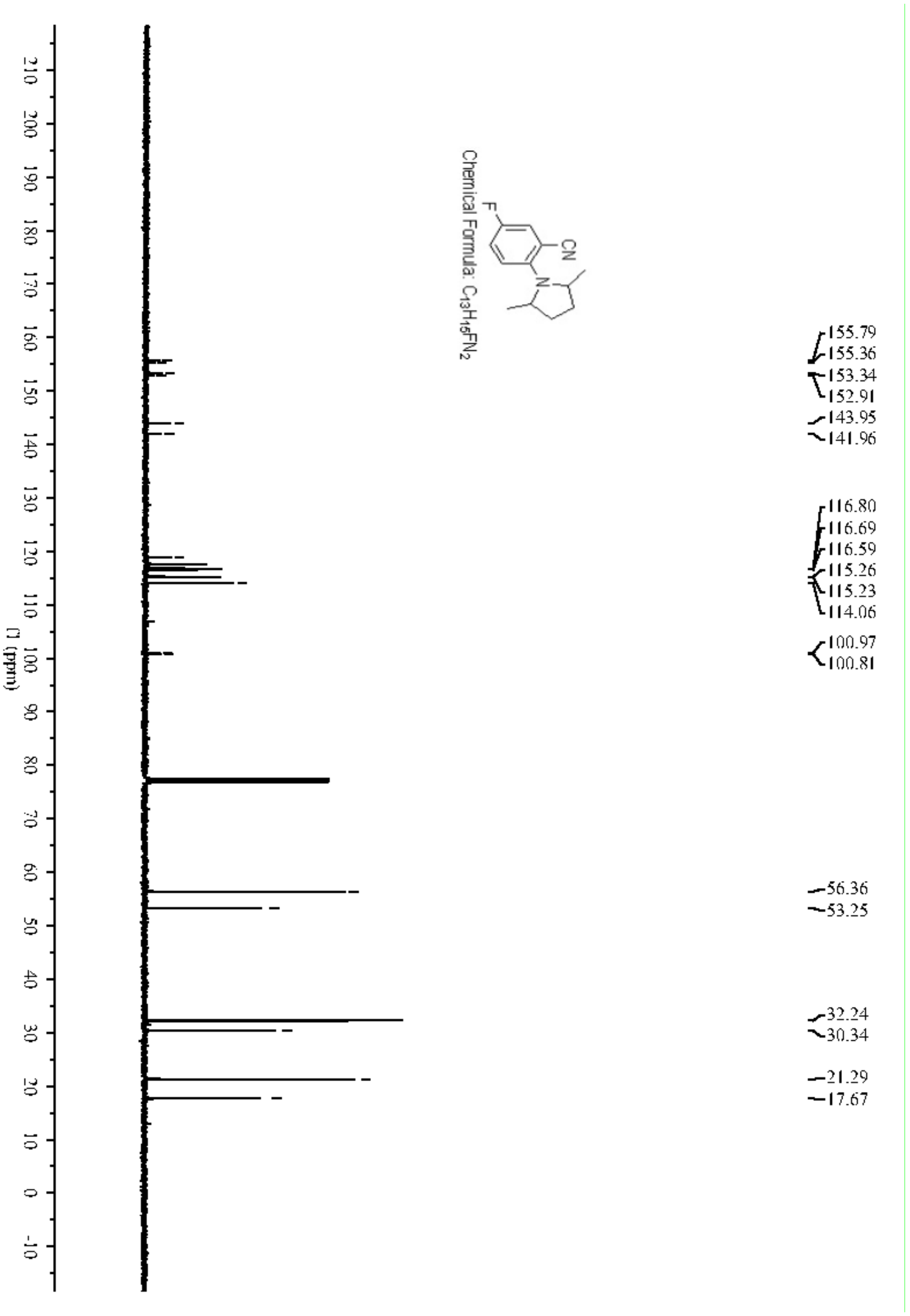

^{13}C NMR (101 MHz, CDCl3) spectrum of **3a12**

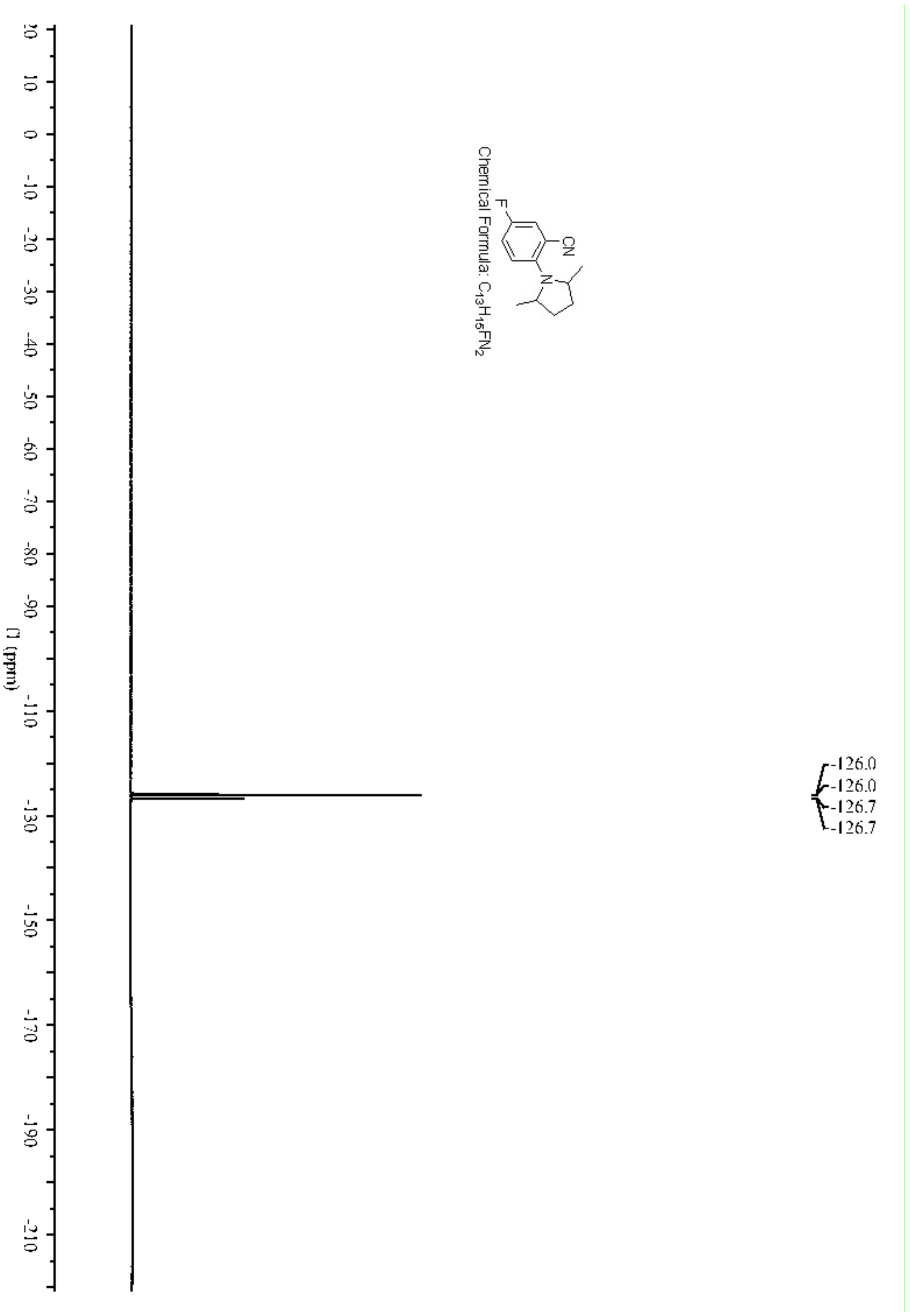

^{19}F NMR (377 MHz, CDCl3) spectrum of **3a12**

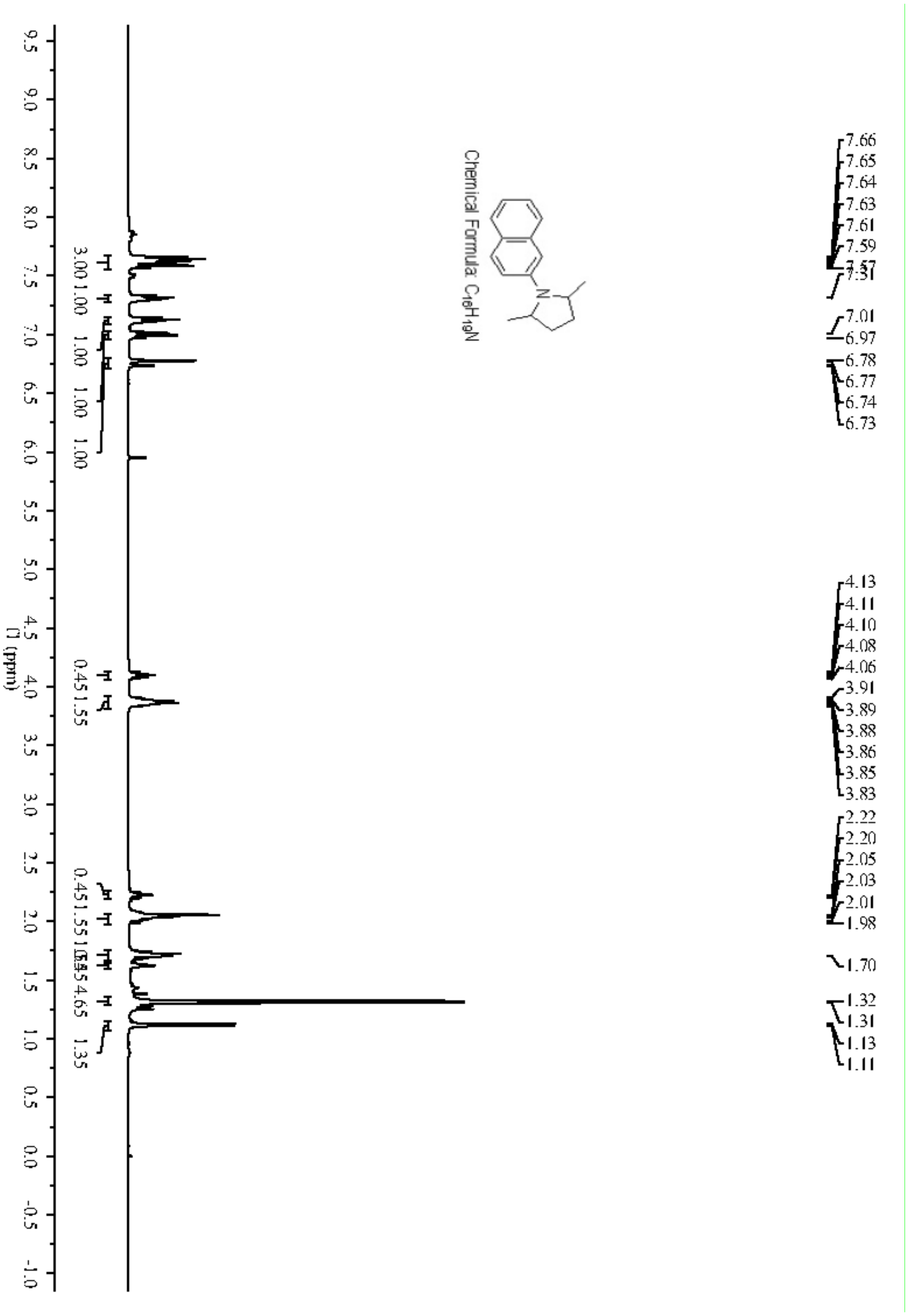

^{1}H NMR (400 MHz, $CDCl_3$) spectrum of **3a13**

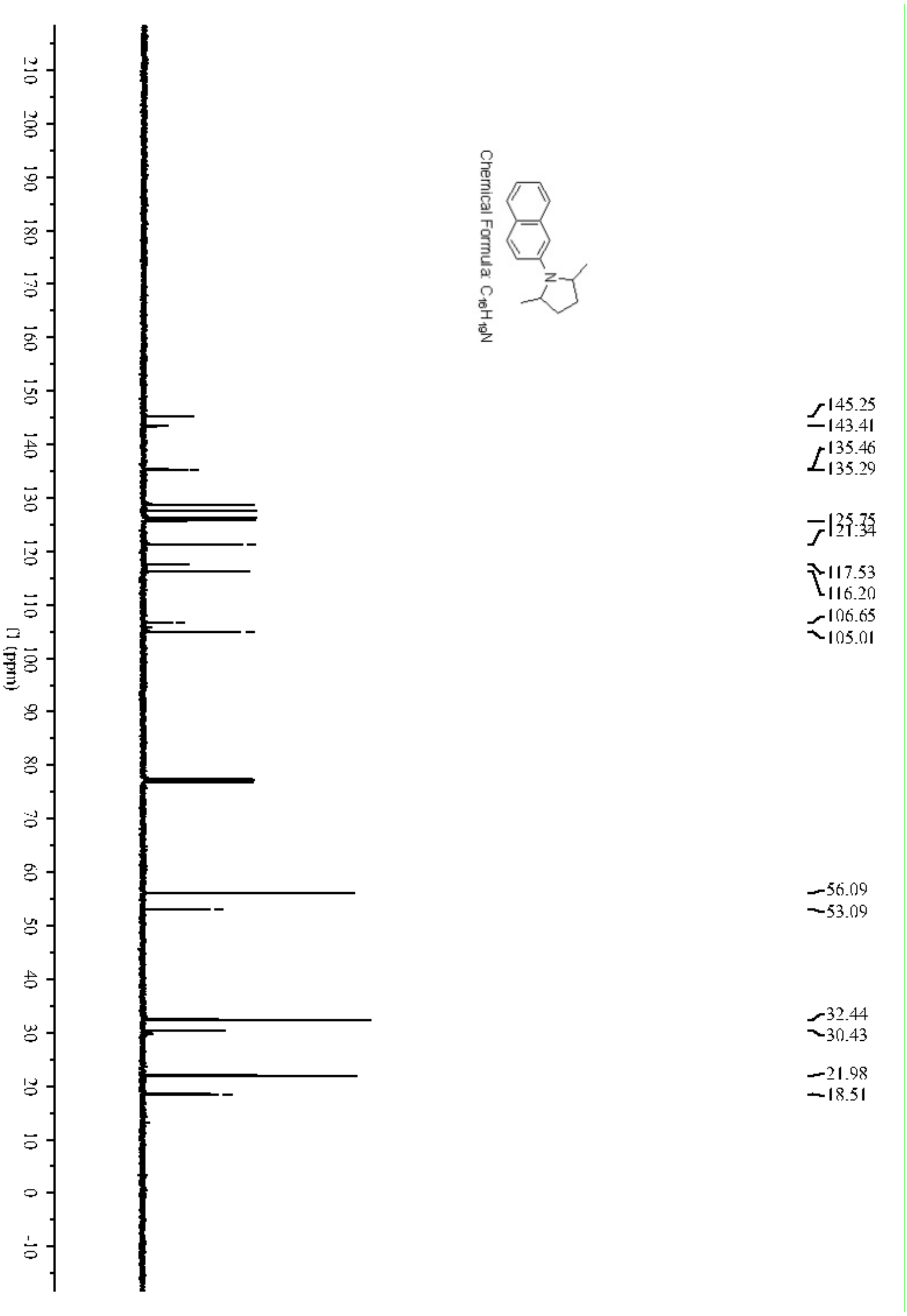

^{13}C NMR (101 MHz, $CDCl_3$) spectrum of **3a13**

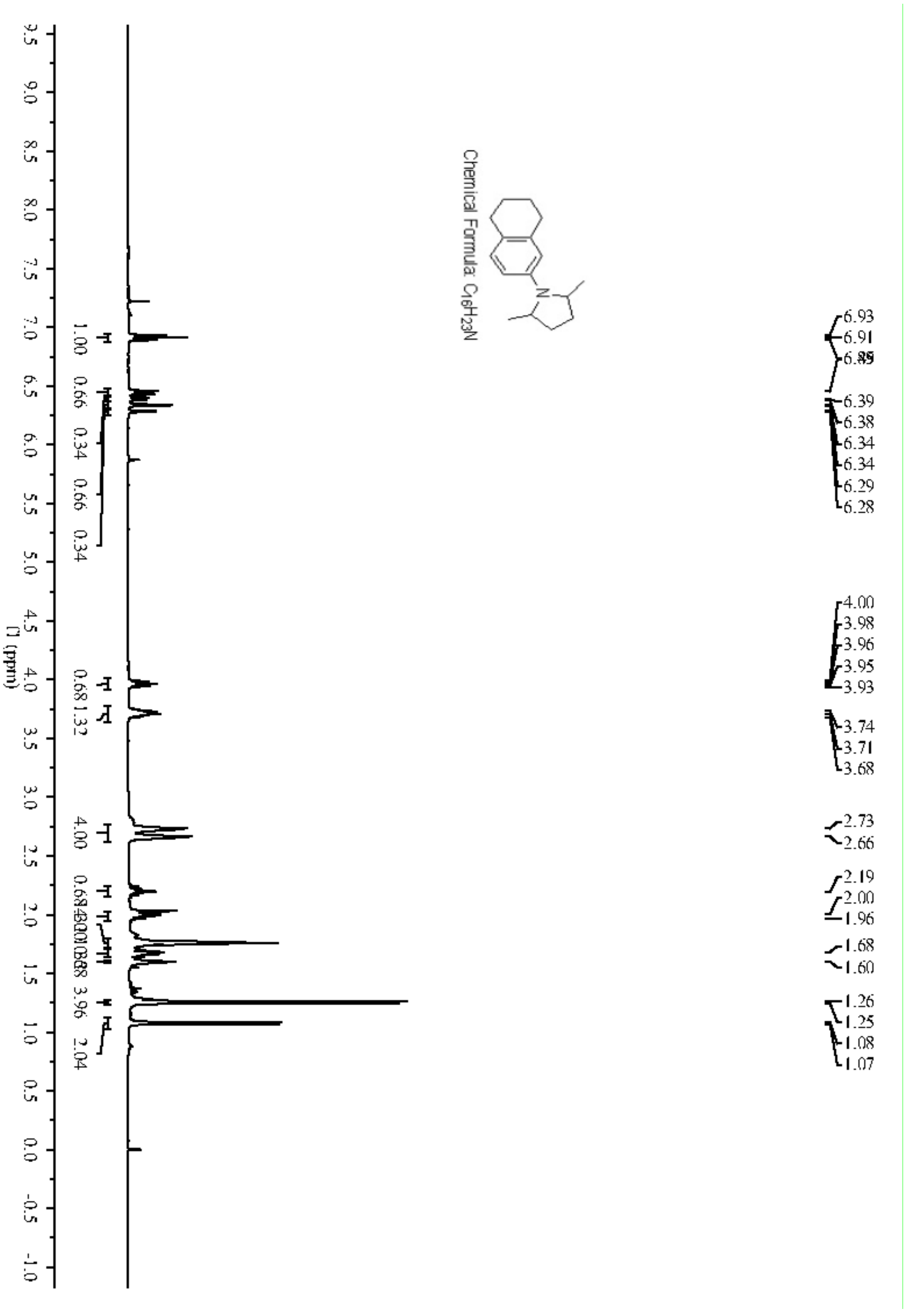

^{1}H NMR (400 MHz, $CDCl_3$) spectrum of **3a14**

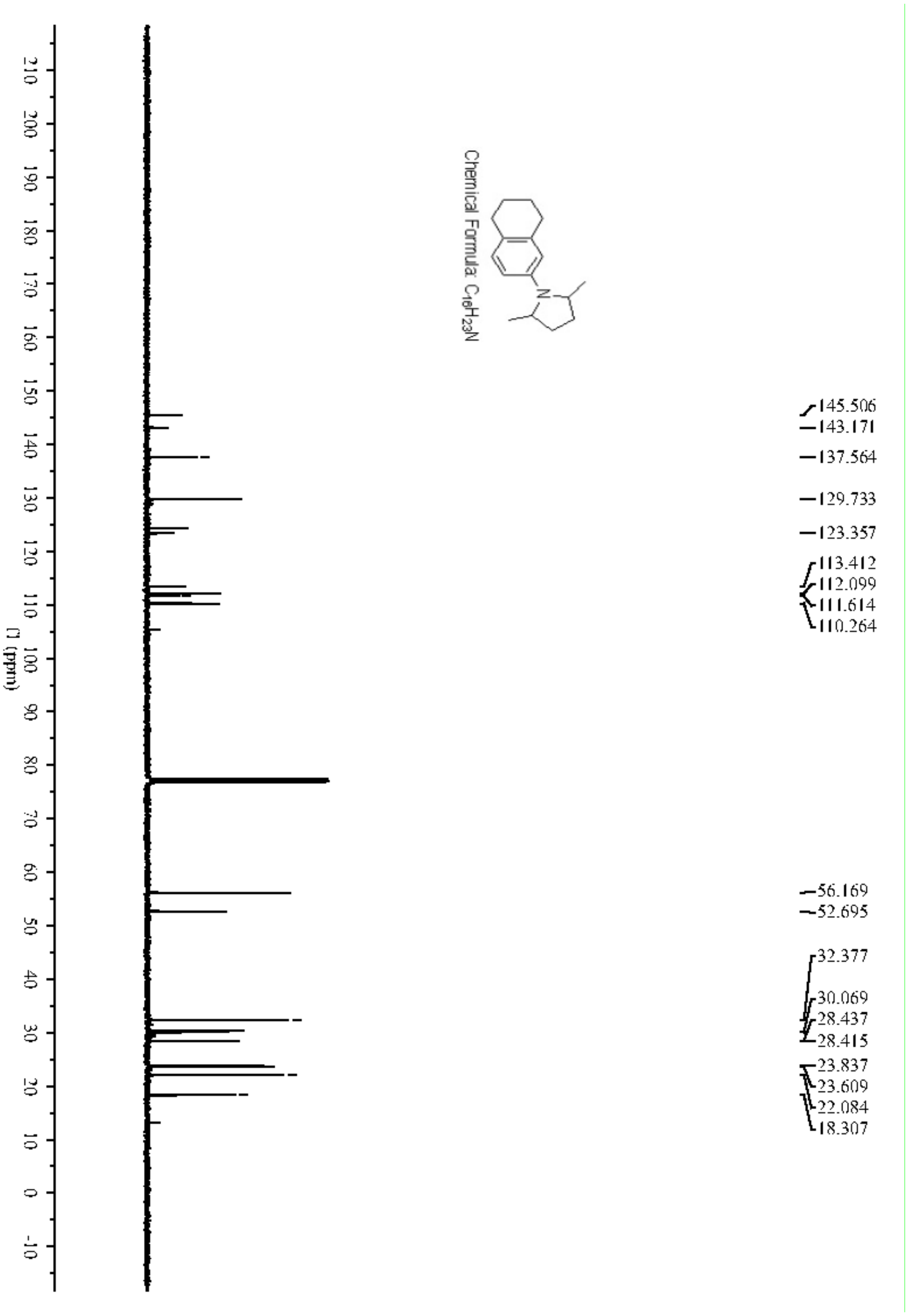

^{13}C NMR (101 MHz, $CDCl_3$) spectrum of **3a14**

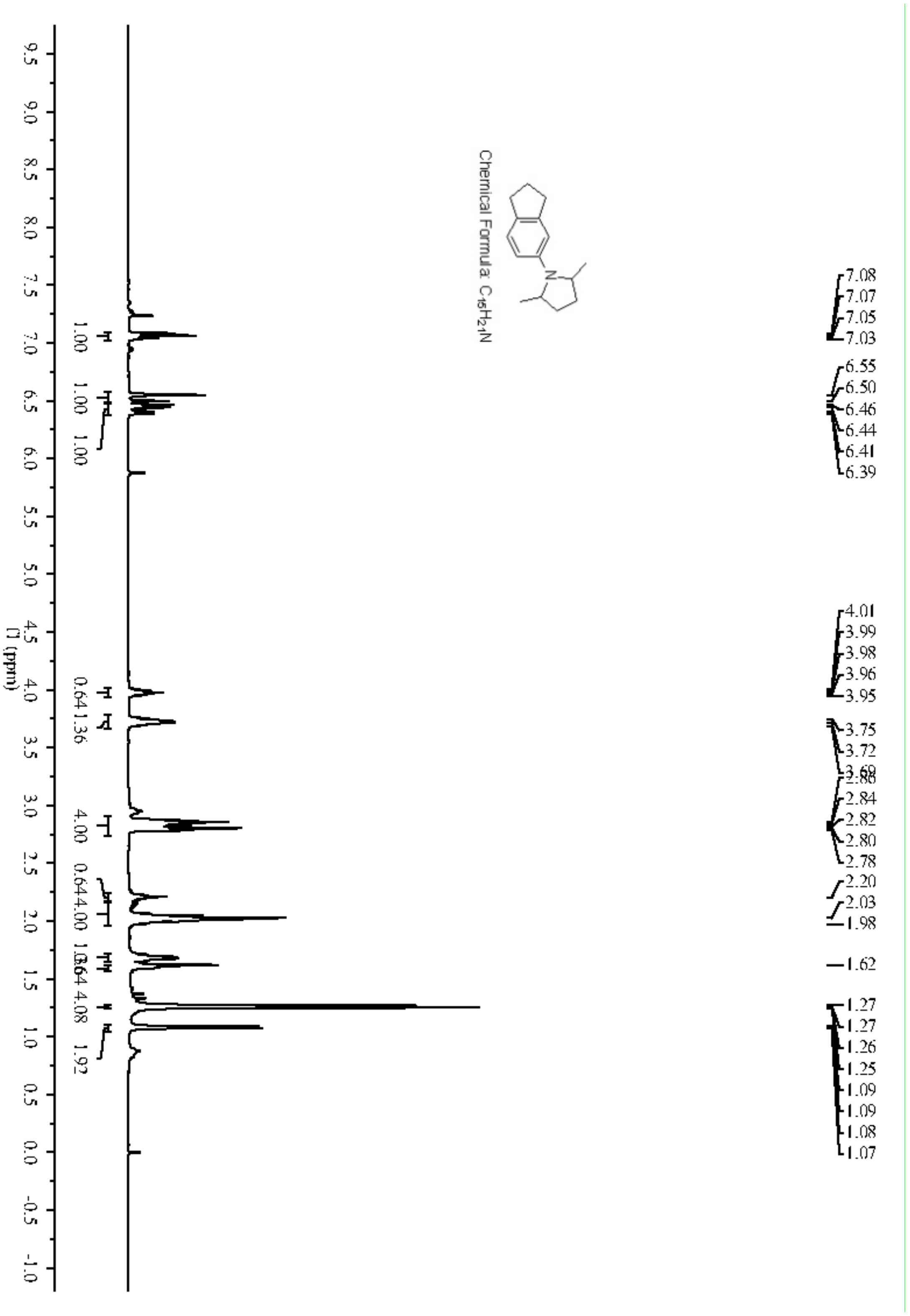

^{1}H NMR (400 MHz, $CDCl_3$) spectrum of **3a15**

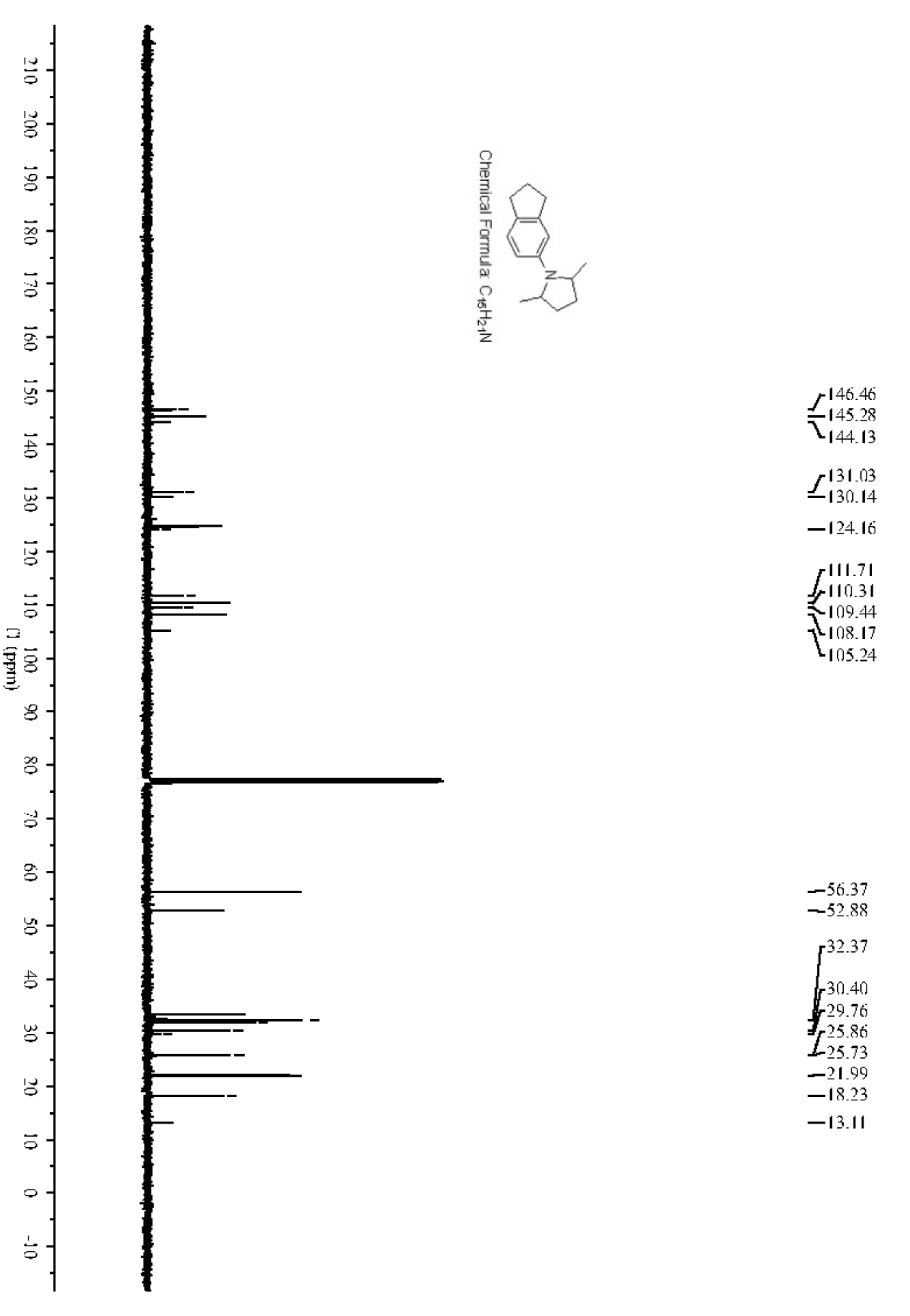

^{13}C NMR (101 MHz, $CDCl_3$) spectrum of **3a15**

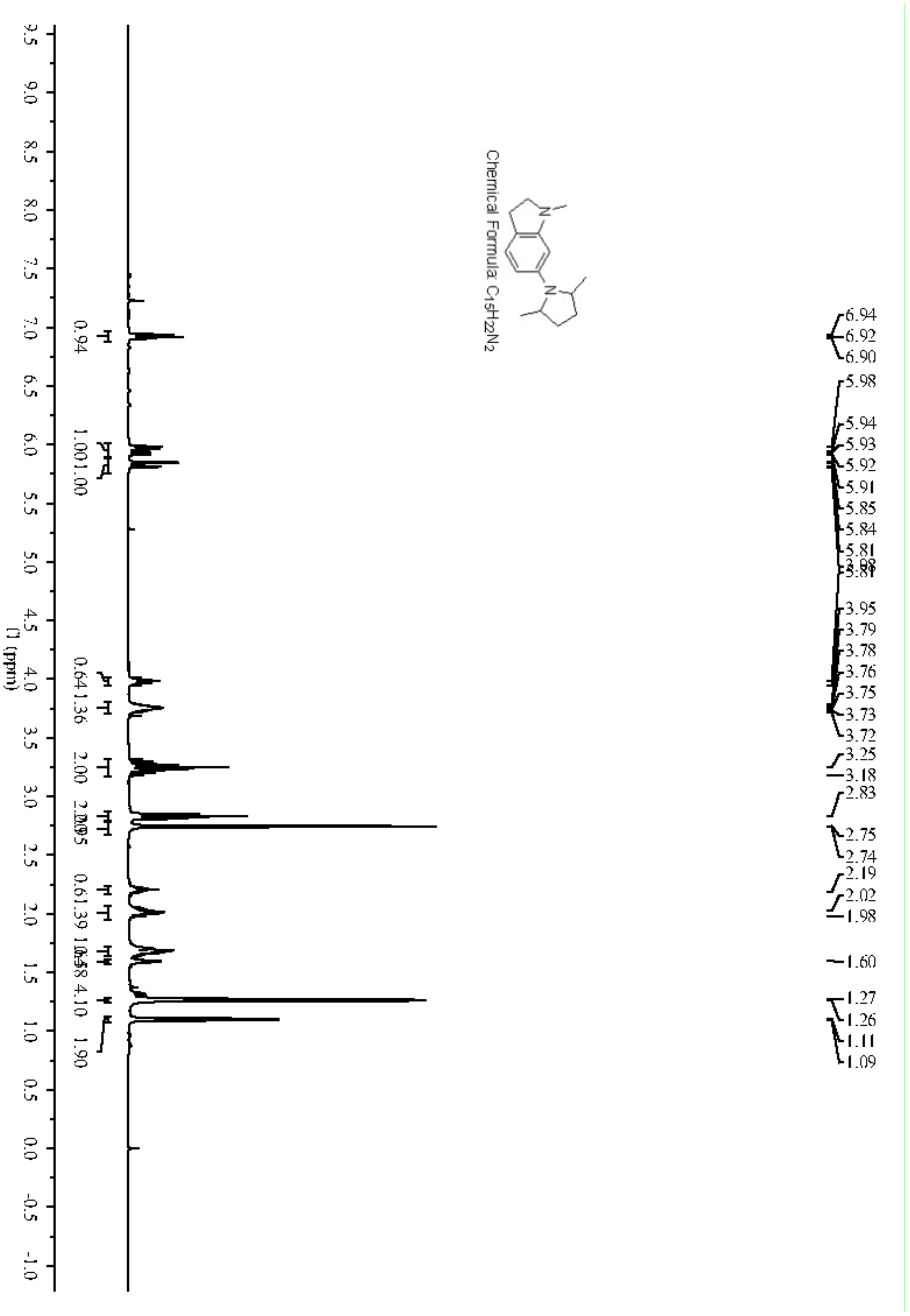

^{1}H NMR (400 MHz, $CDCl_3$) spectrum of **3a16**

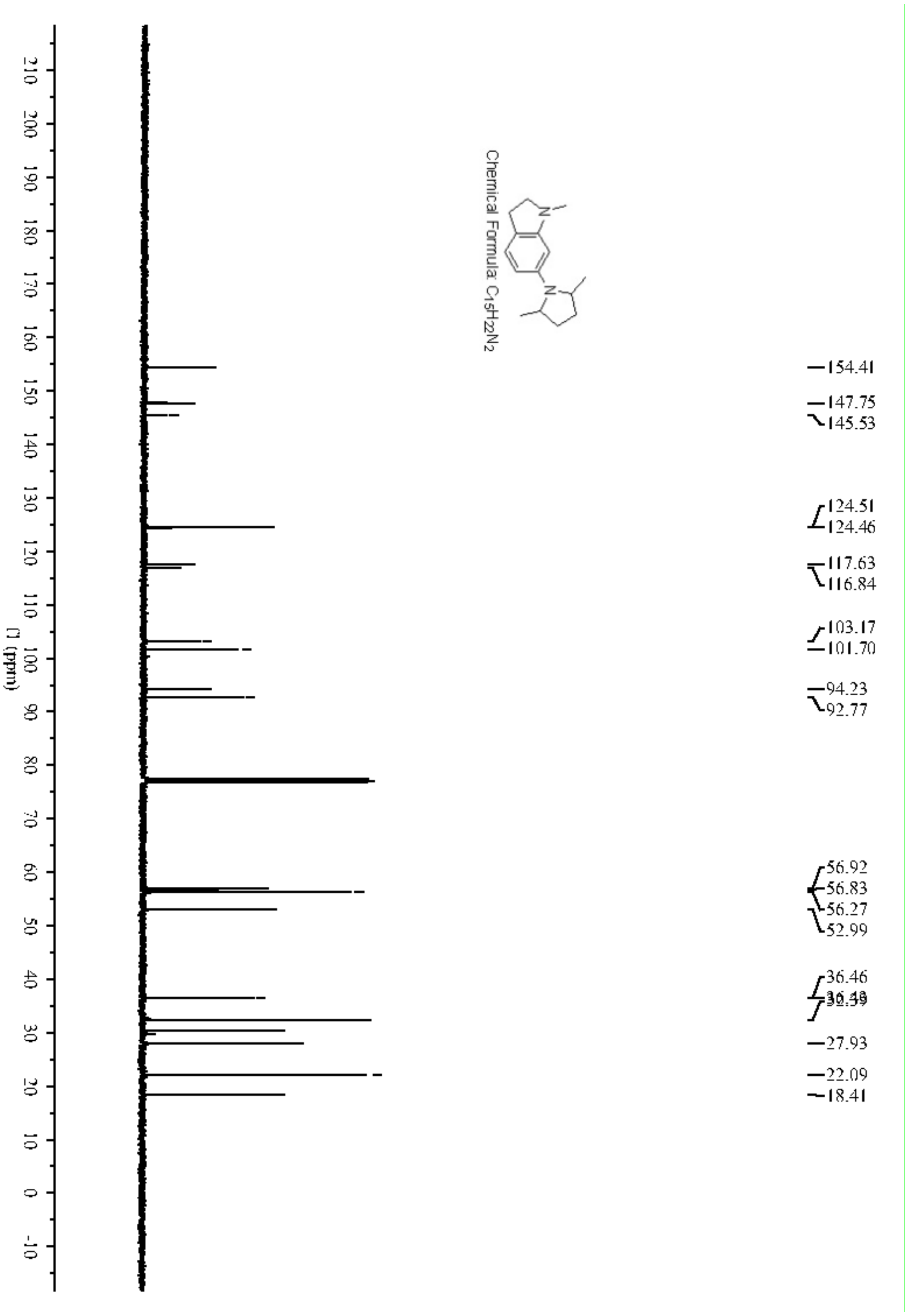

^{13}C NMR (101 MHz, $CDCl_3$) spectrum of **3a16**

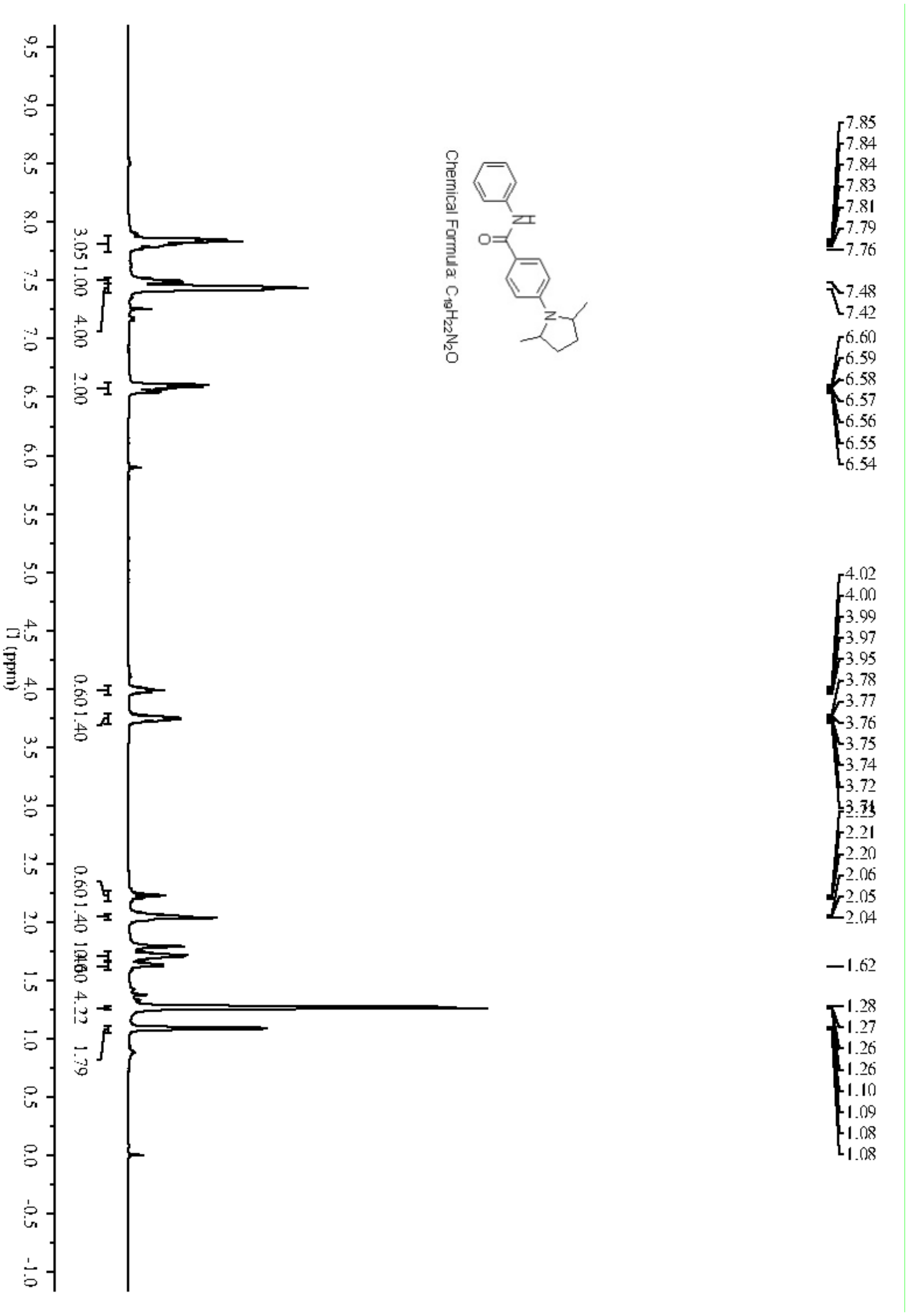

^{1}H NMR (400 MHz, $CDCl_3$) spectrum of **3a17**

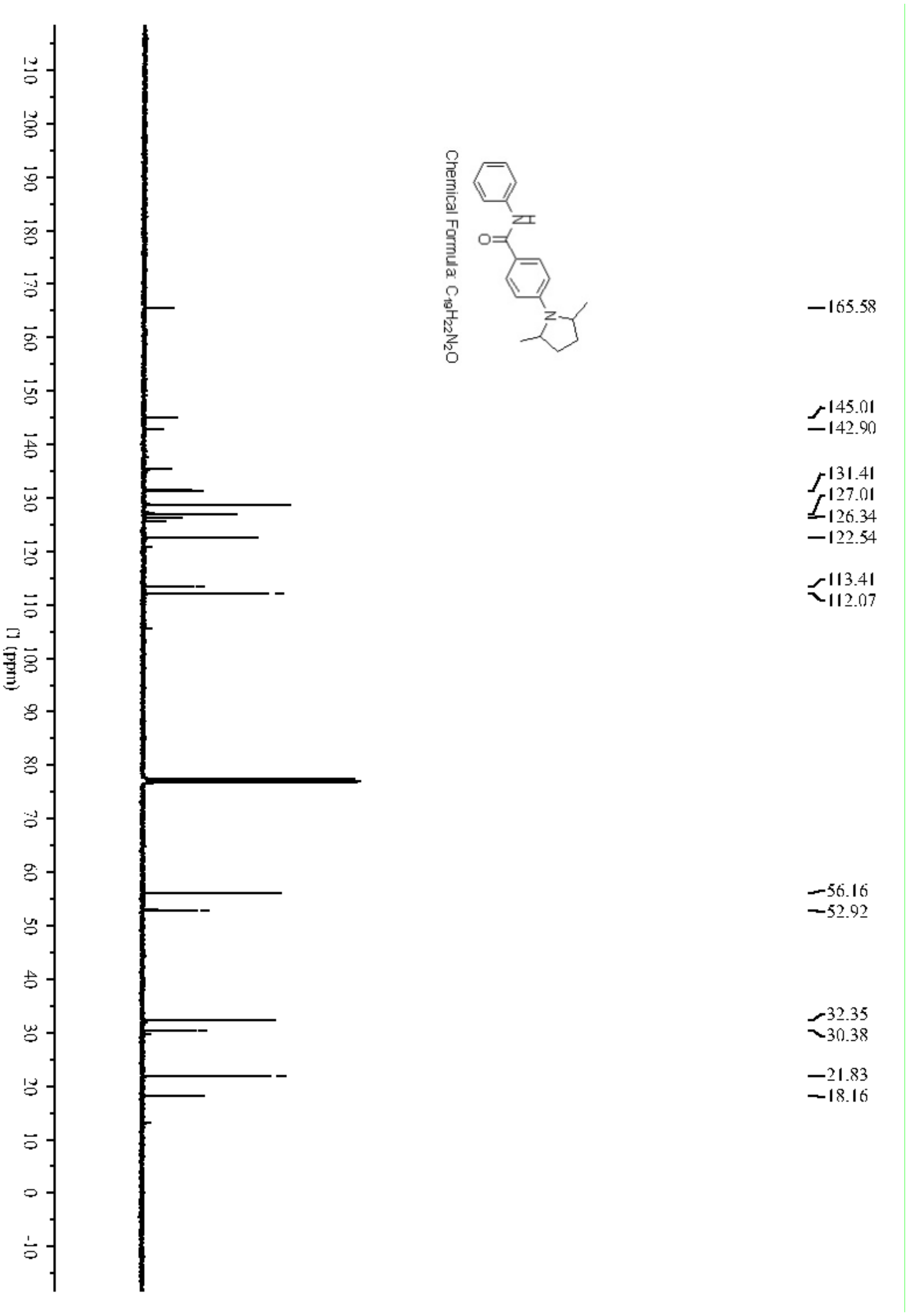

^{13}C NMR (101 MHz, $CDCl_3$) spectrum of **3a17**

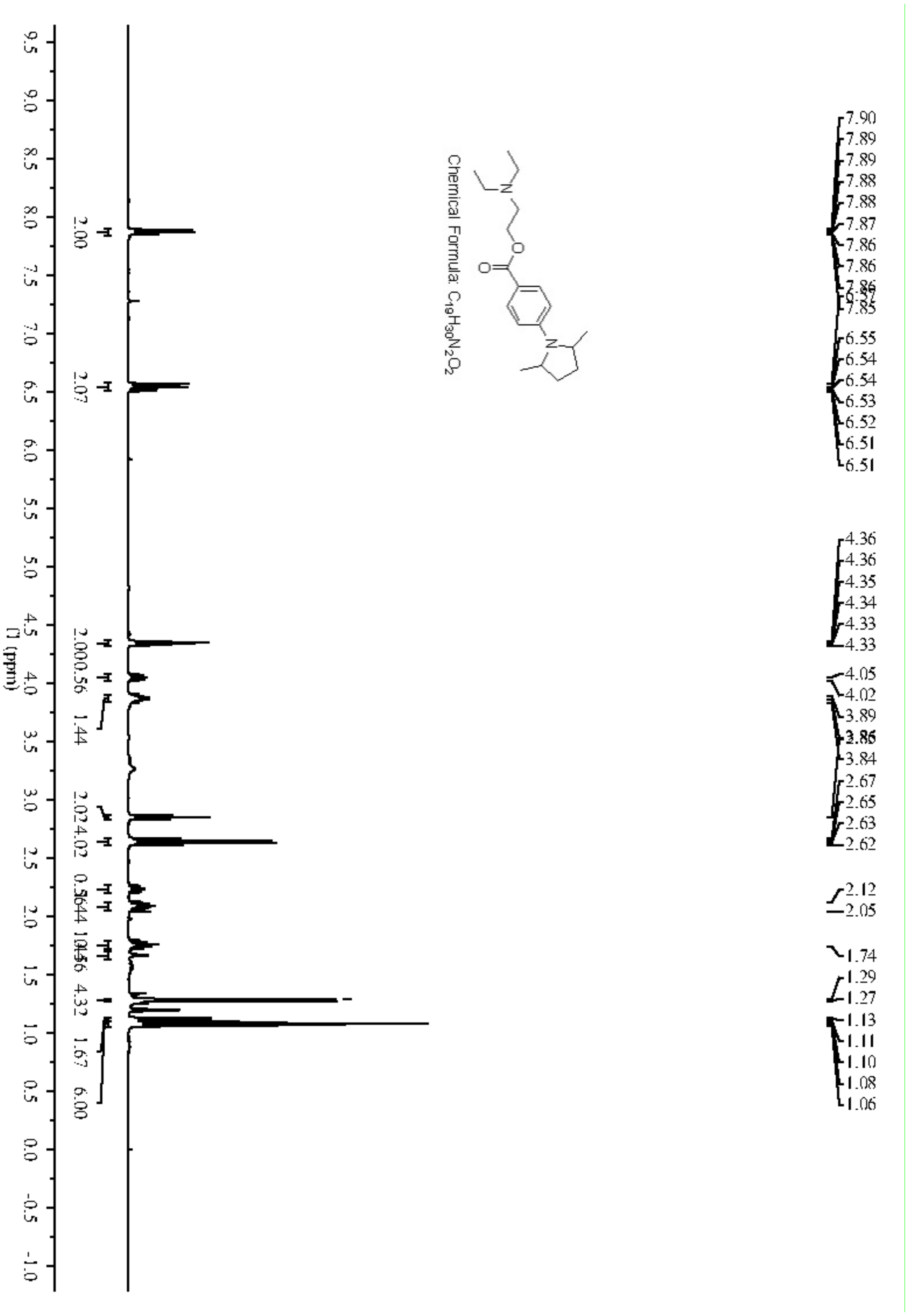

^{1}H NMR (400 MHz, $CDCl_3$) spectrum of **3a18**

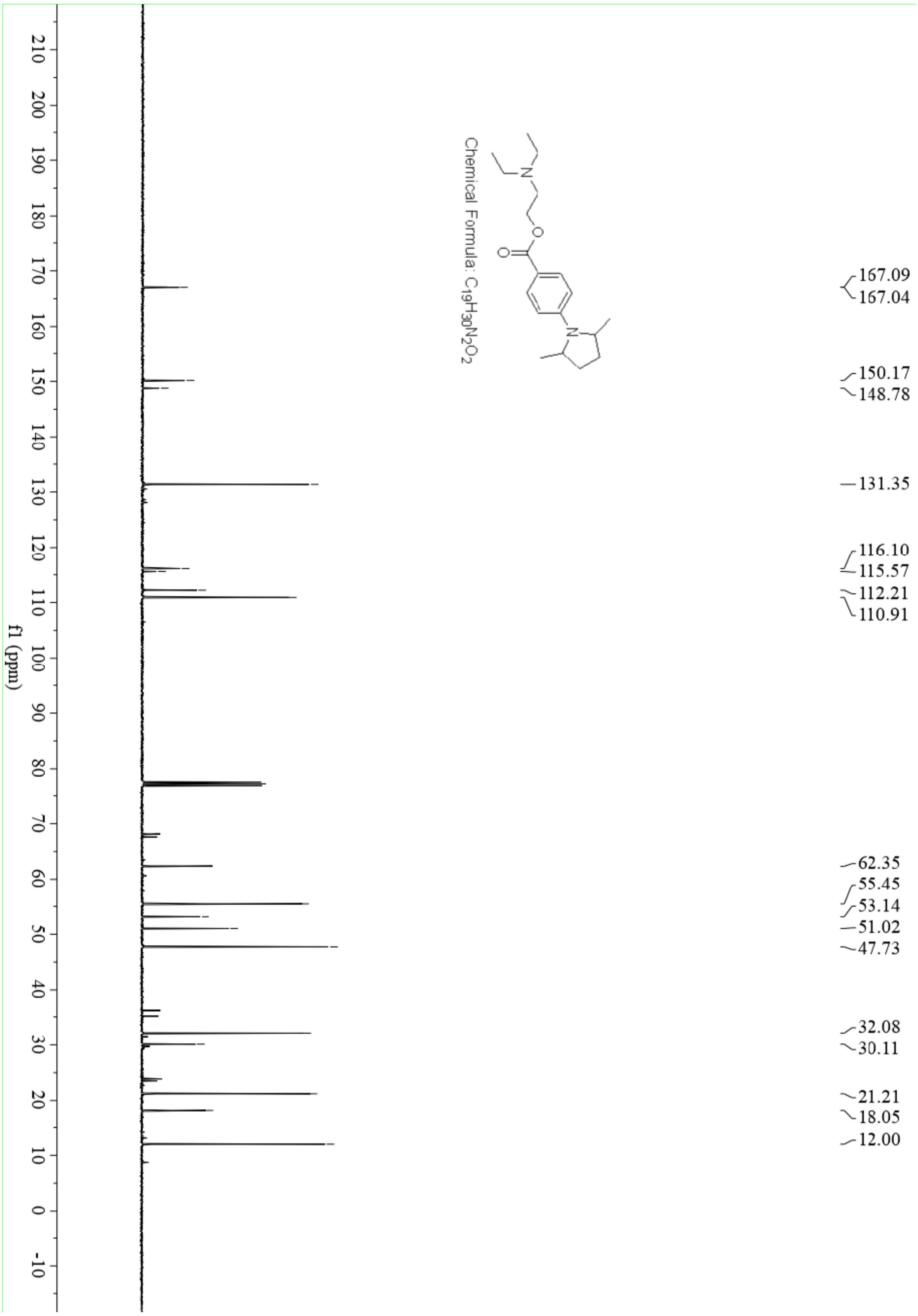

^{13}C NMR (101 MHz, $CDCl_3$) spectrum of **3a18**

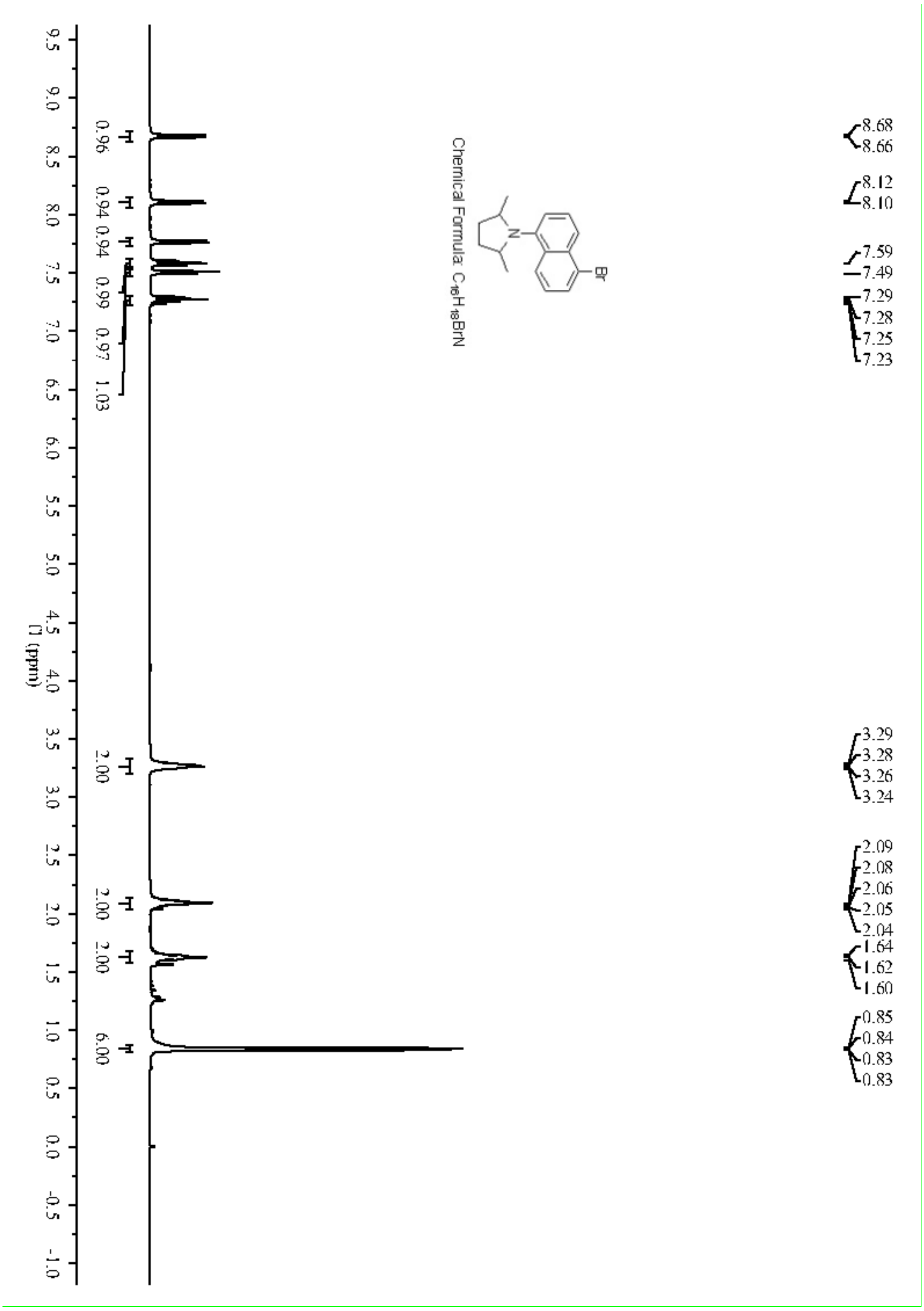

^{1}H NMR (400 MHz, $CDCl_3$) spectrum of **3a19**

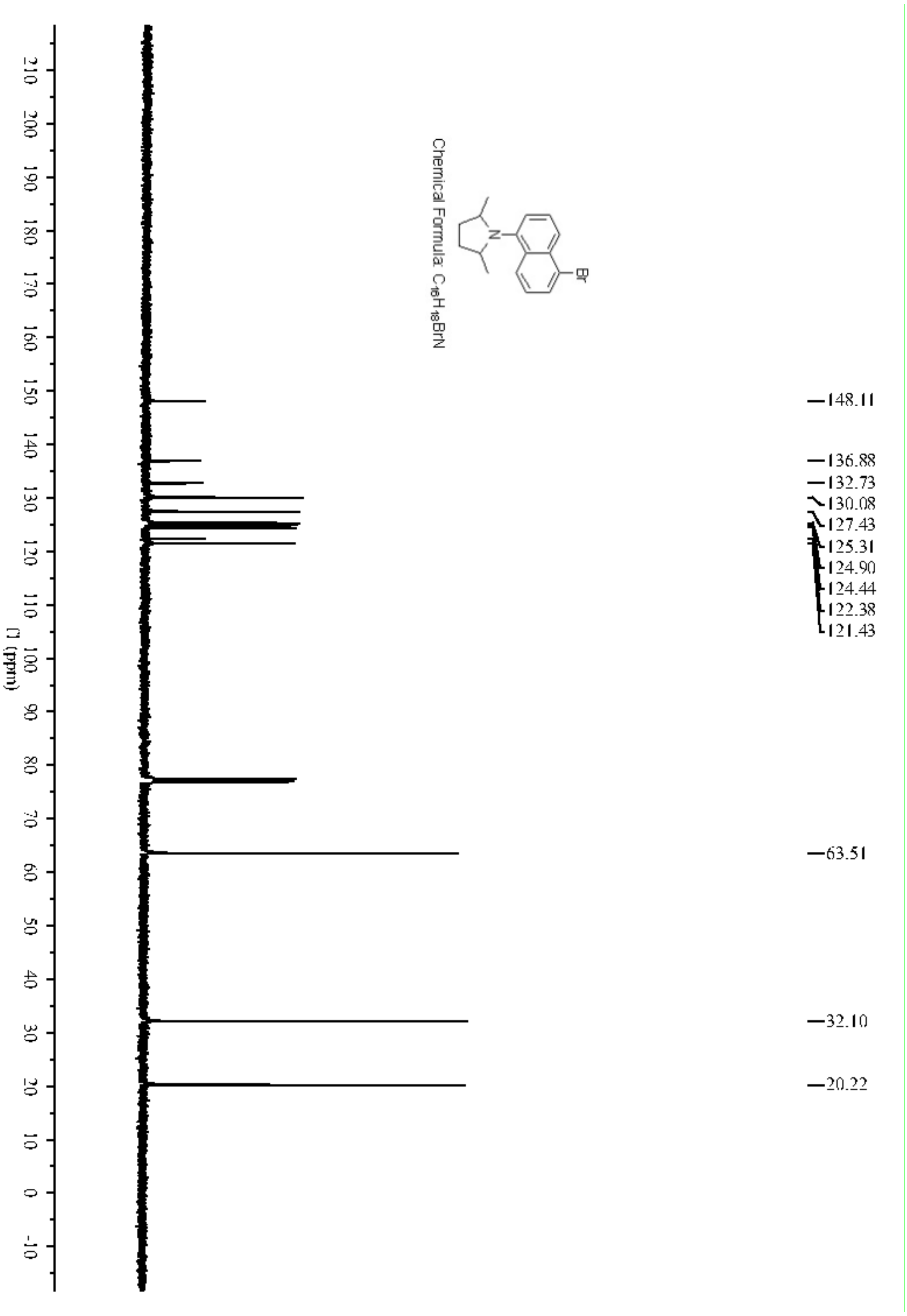

^{13}C NMR (101 MHz, $CDCl_3$) spectrum of **3a19**

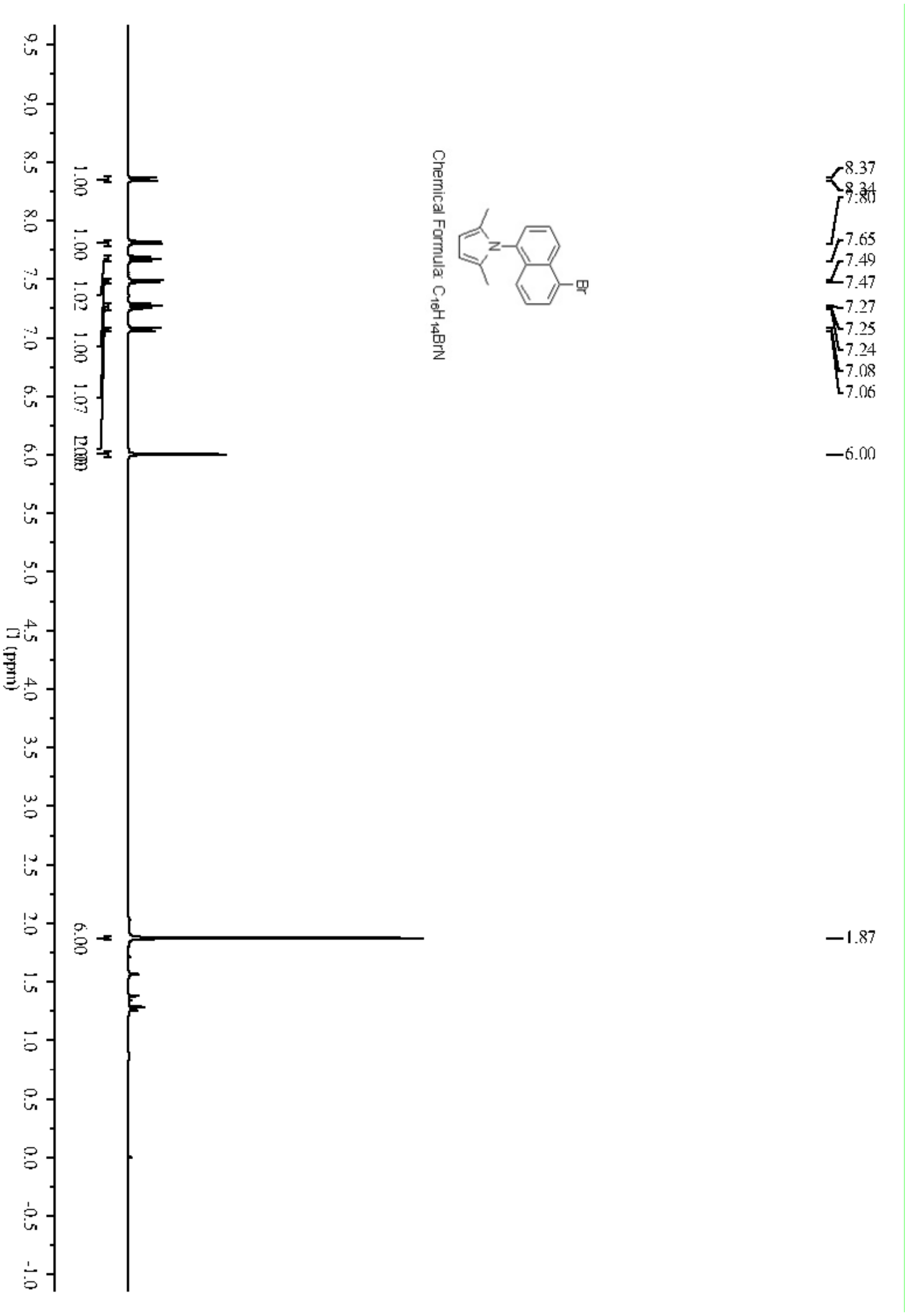

^{1}H NMR (400 MHz, $CDCl_3$) spectrum of **3a19’**

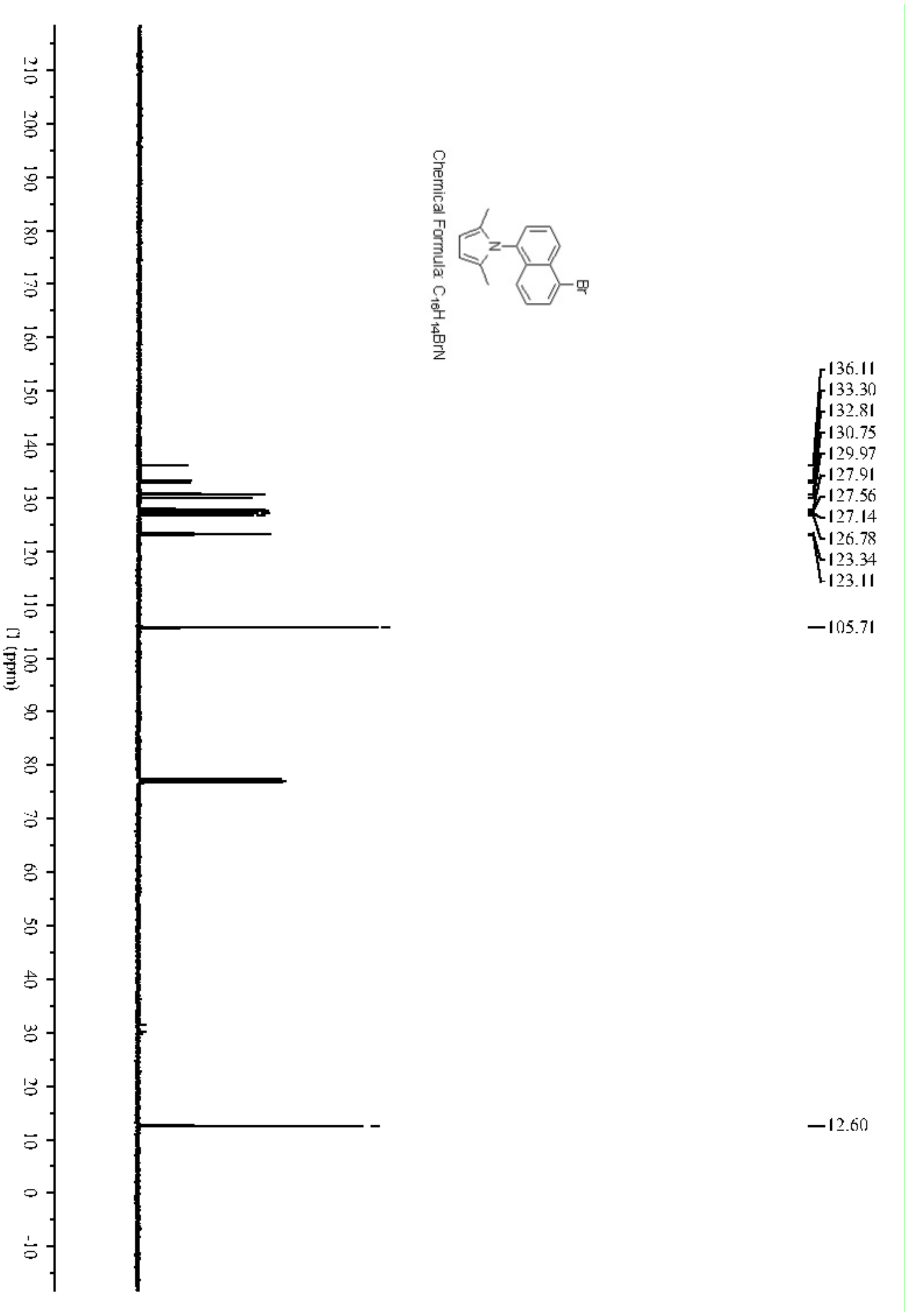

^{13}C NMR (101 MHz, CDCl$_3$) spectrum of **3a19'**

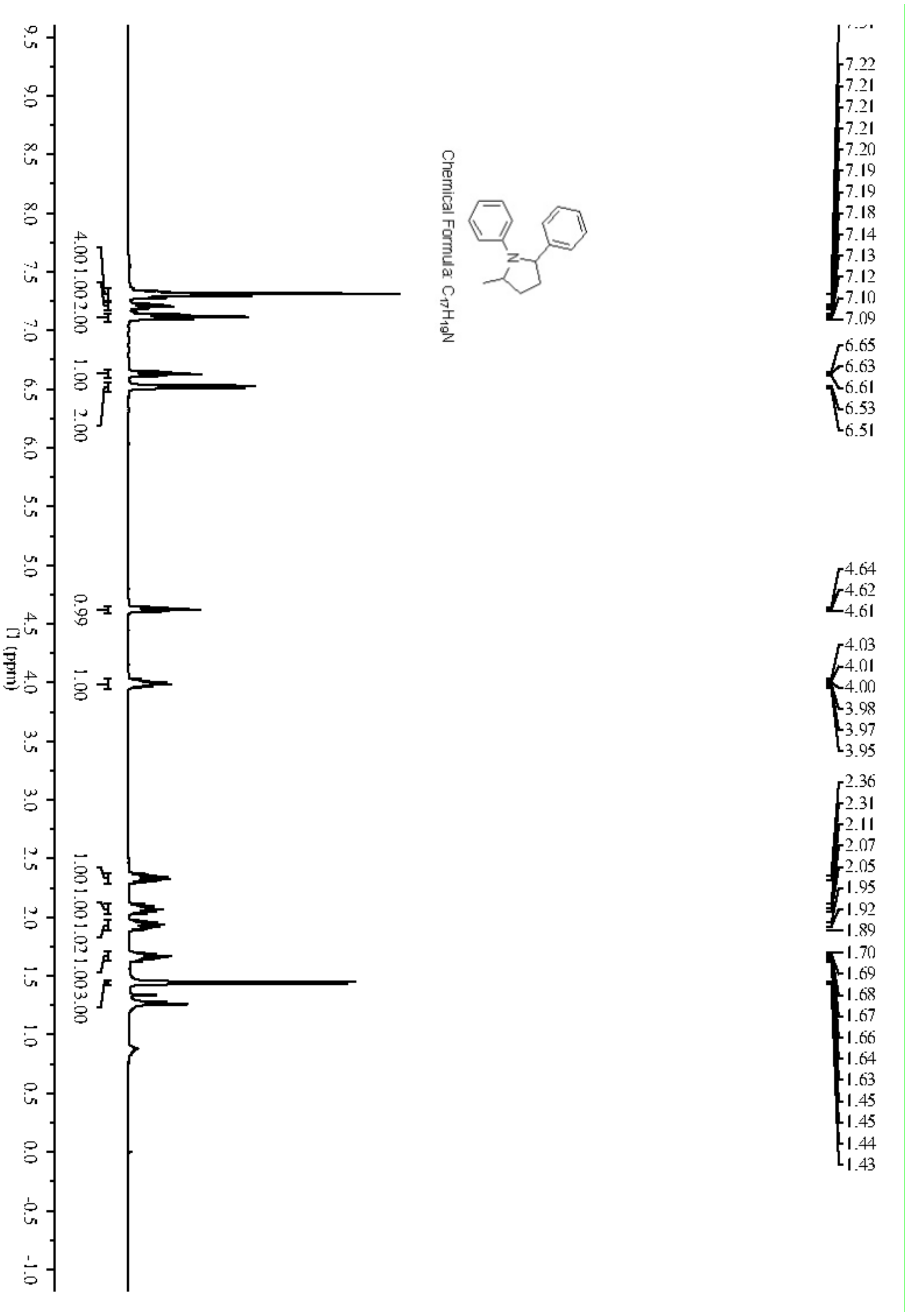

^{1}H NMR (400 MHz, CDCl3) spectrum of **3b1**

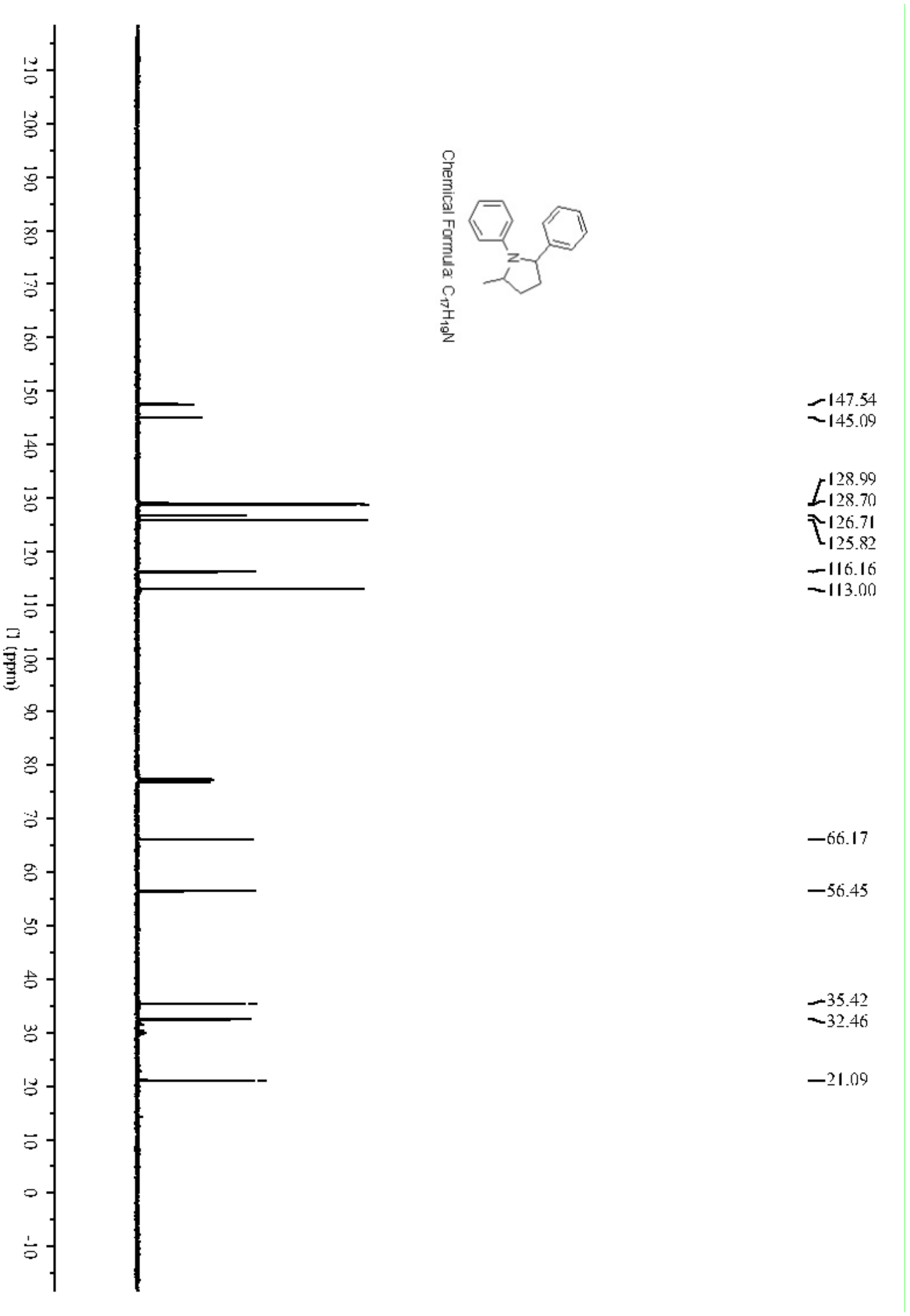

^{13}C NMR (101 MHz, $CDCl_3$) spectrum of **3b1**

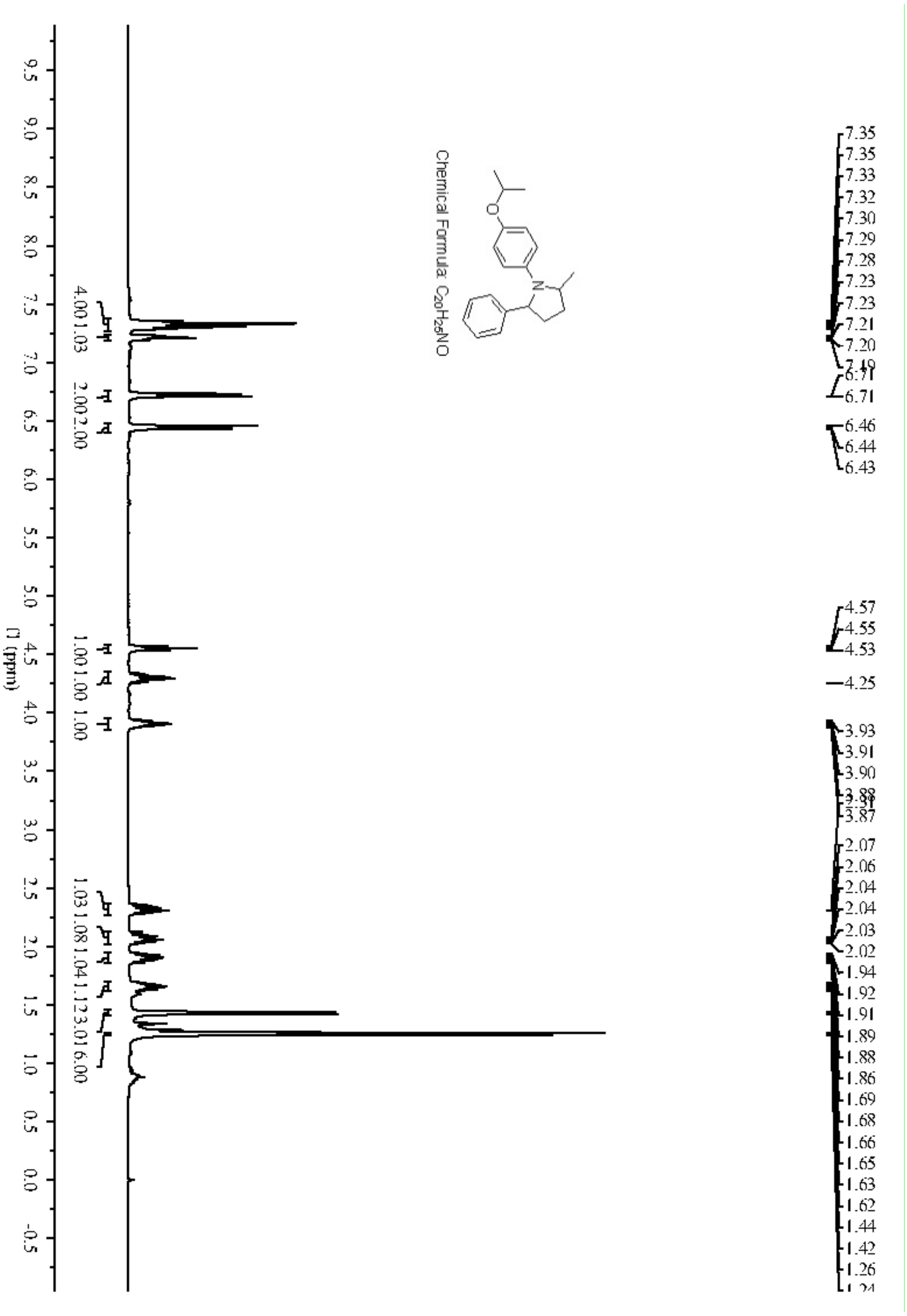

^{1}H NMR (400 MHz, $CDCl_3$) spectrum of **3b2**

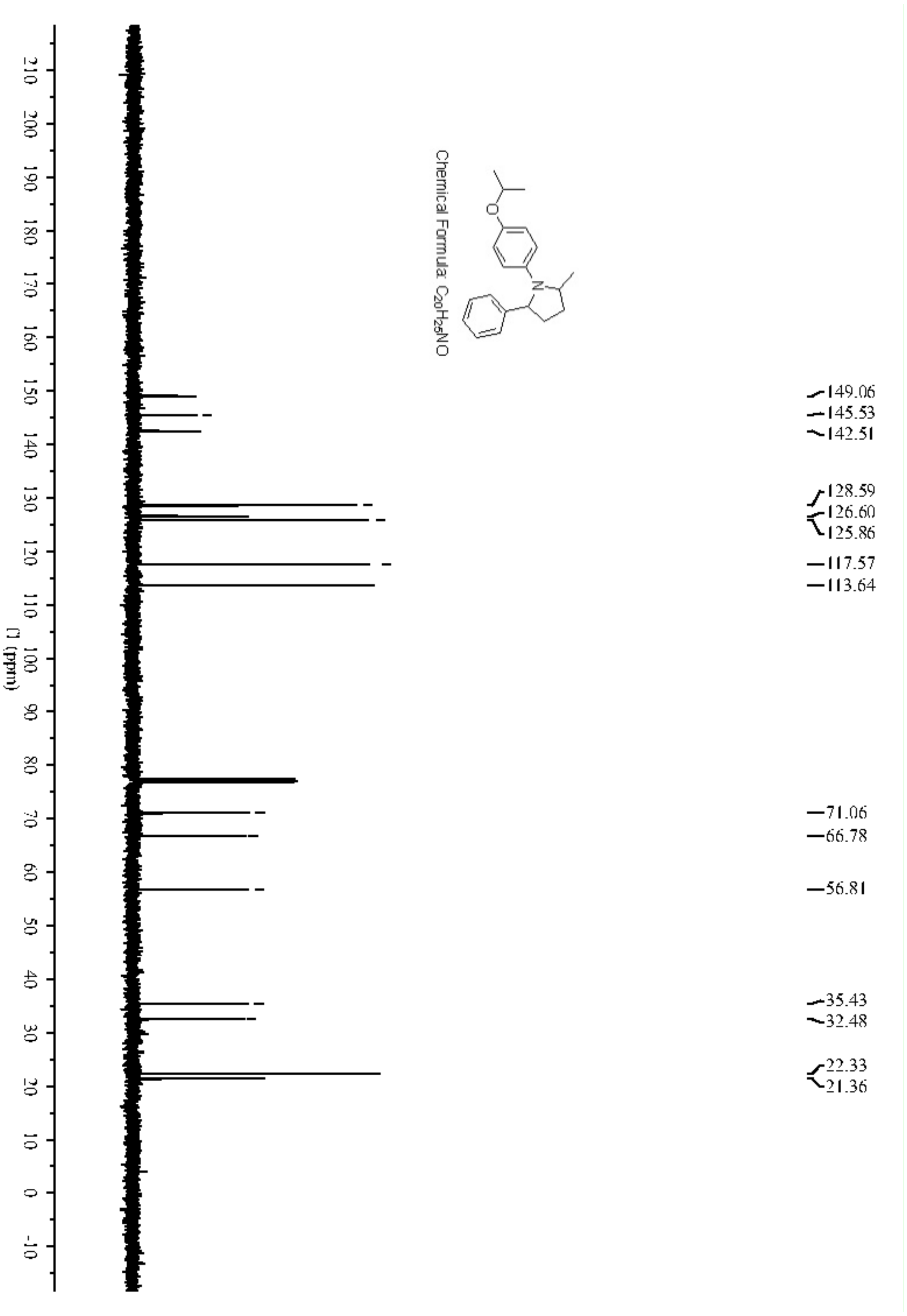

^{13}C NMR (101 MHz, $CDCl_3$) spectrum of **3b2**

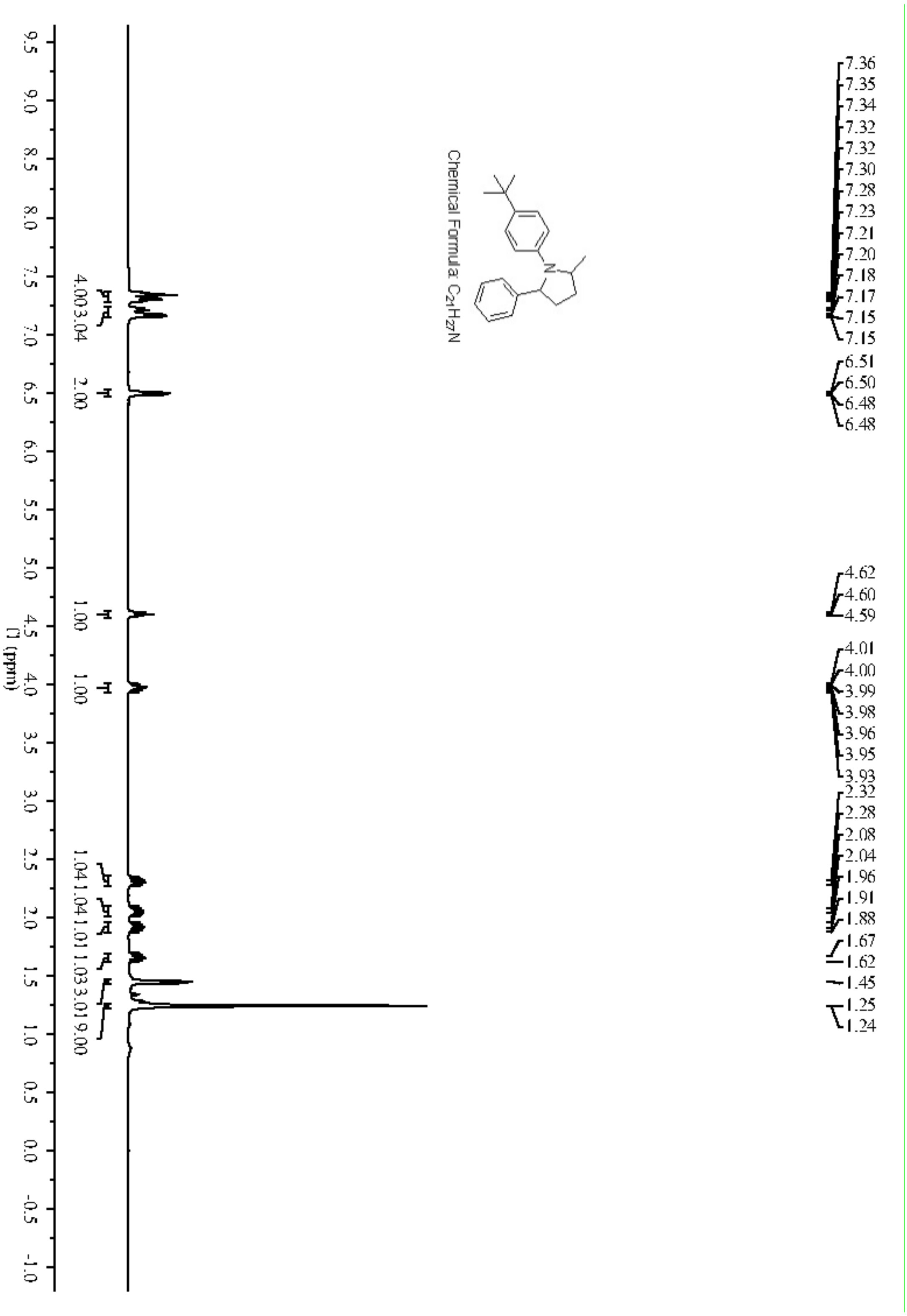

^{1}H NMR (400 MHz, $CDCl_3$) spectrum of **3b3**

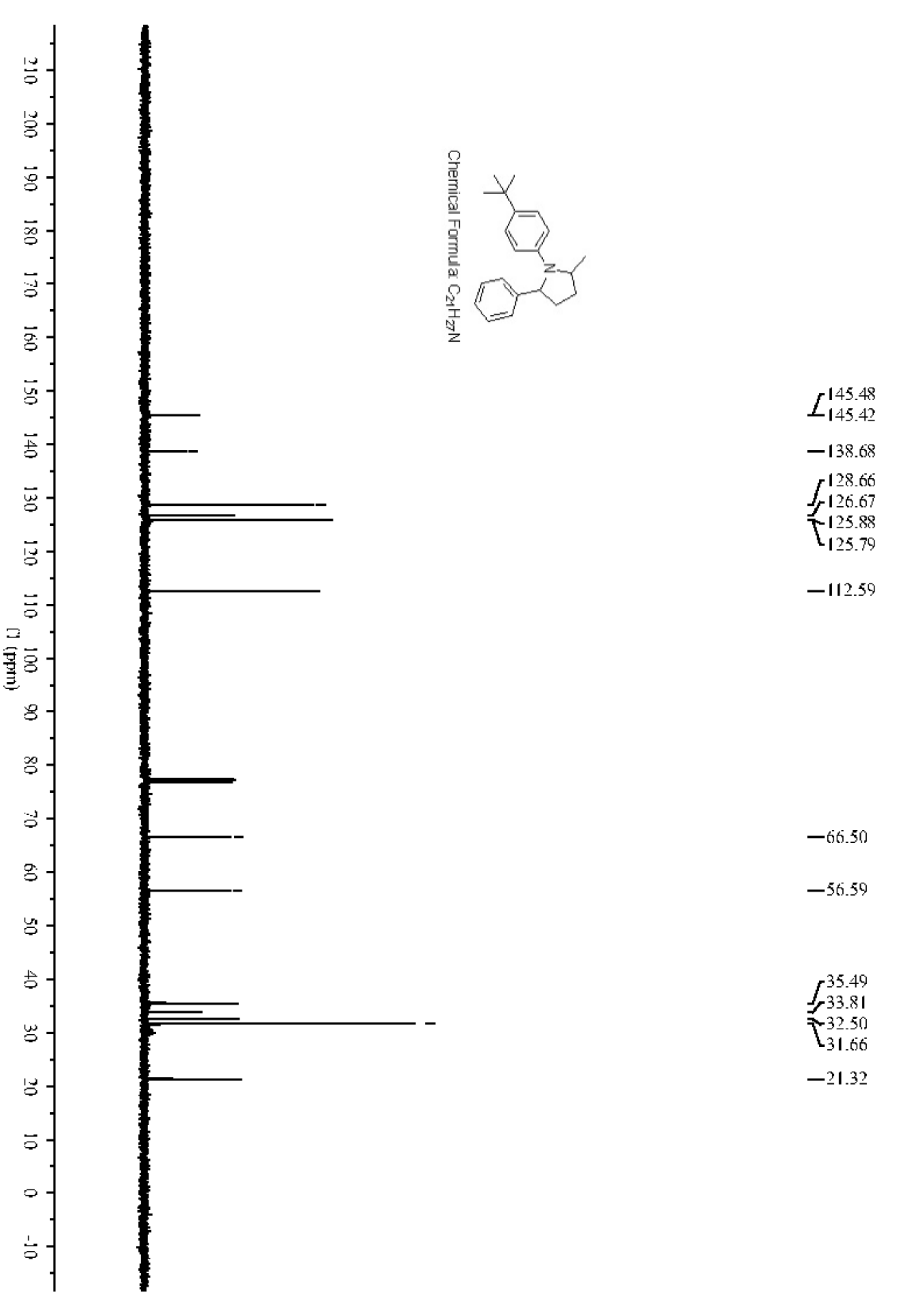

^{13}C NMR (101 MHz, $CDCl_3$) spectrum of **3b3**

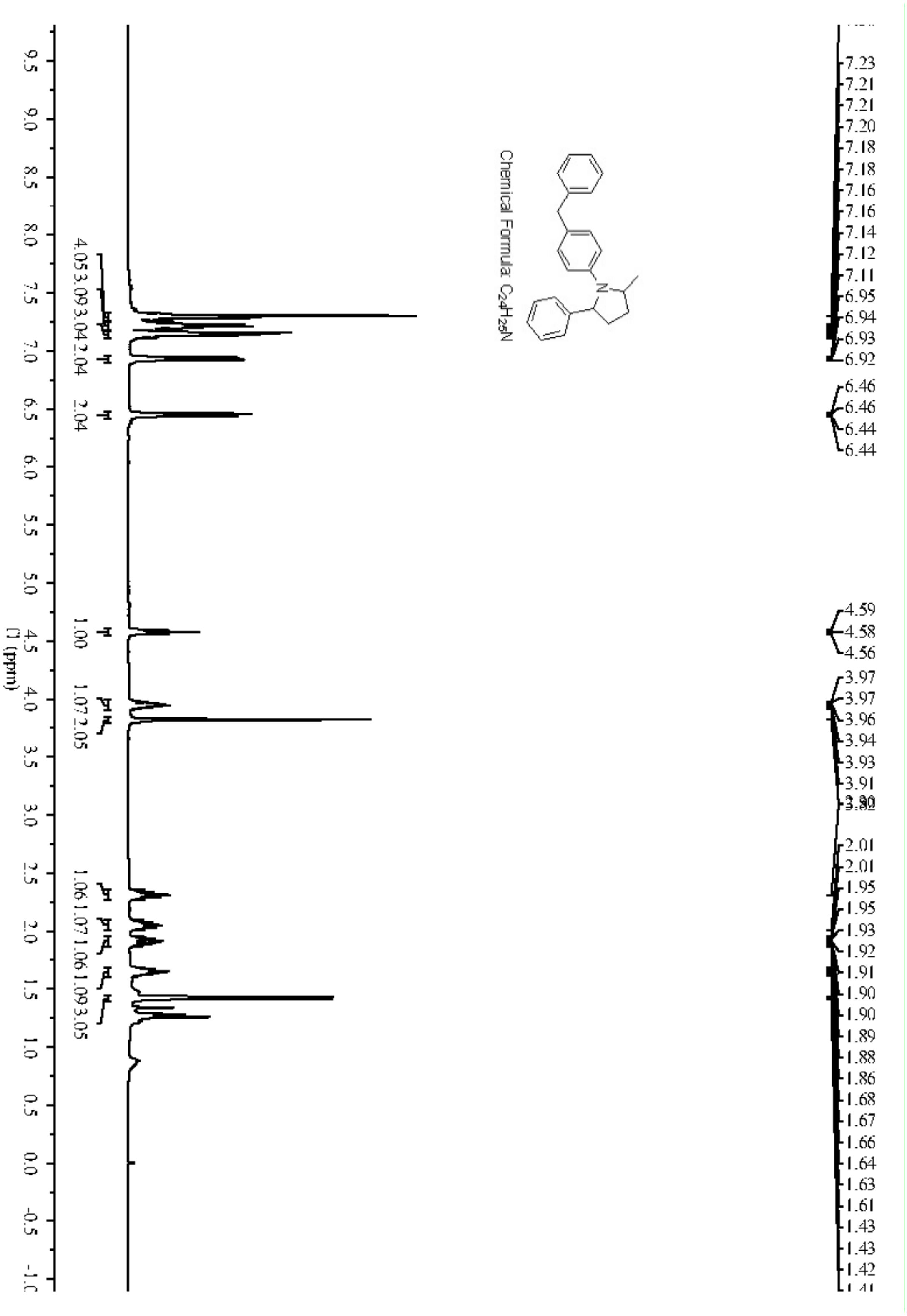

^{1}H NMR (400 MHz, $CDCl_3$) spectrum of **3b4**

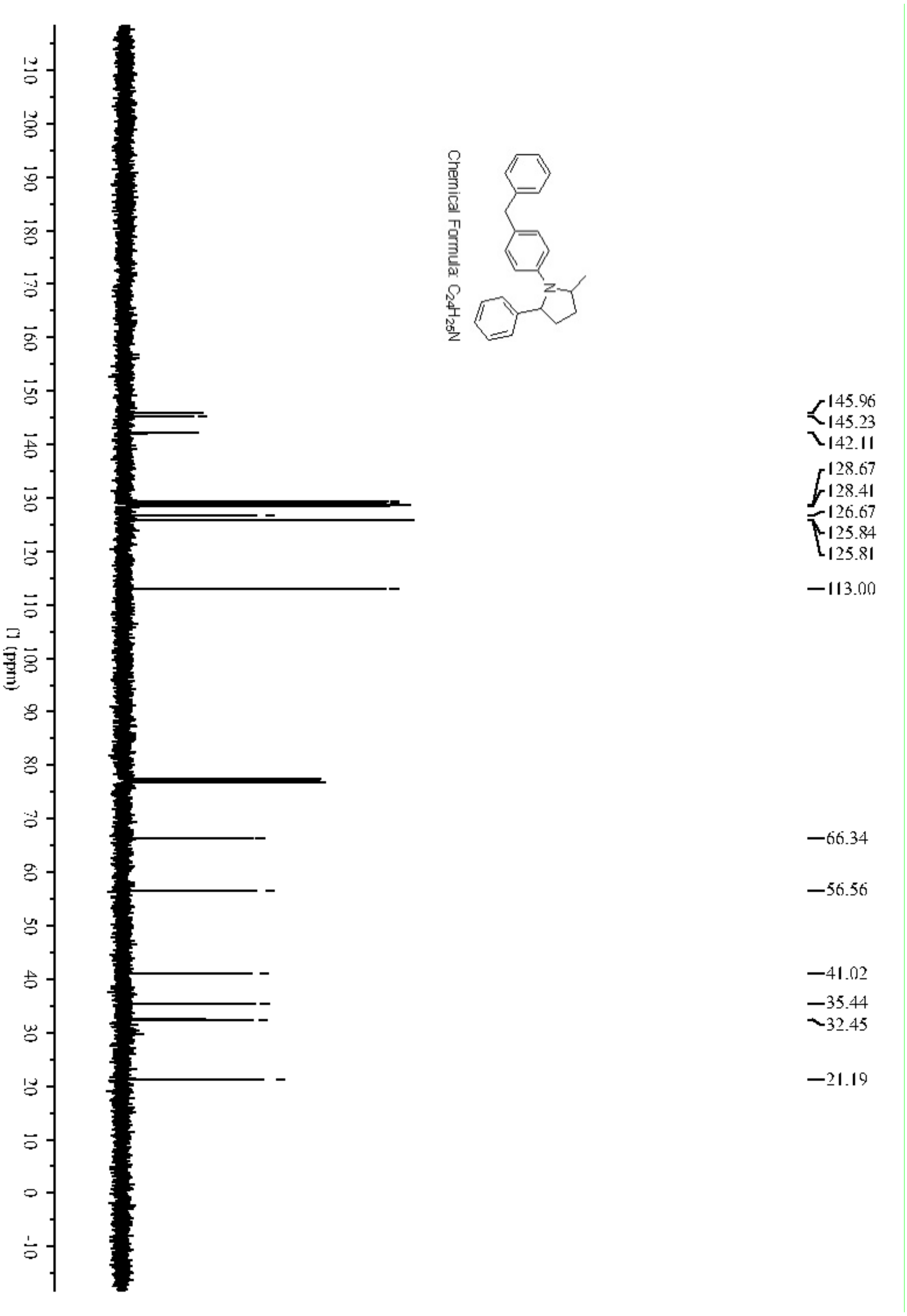

^{13}C NMR (101 MHz, $CDCl_3$) spectrum of **3b4**

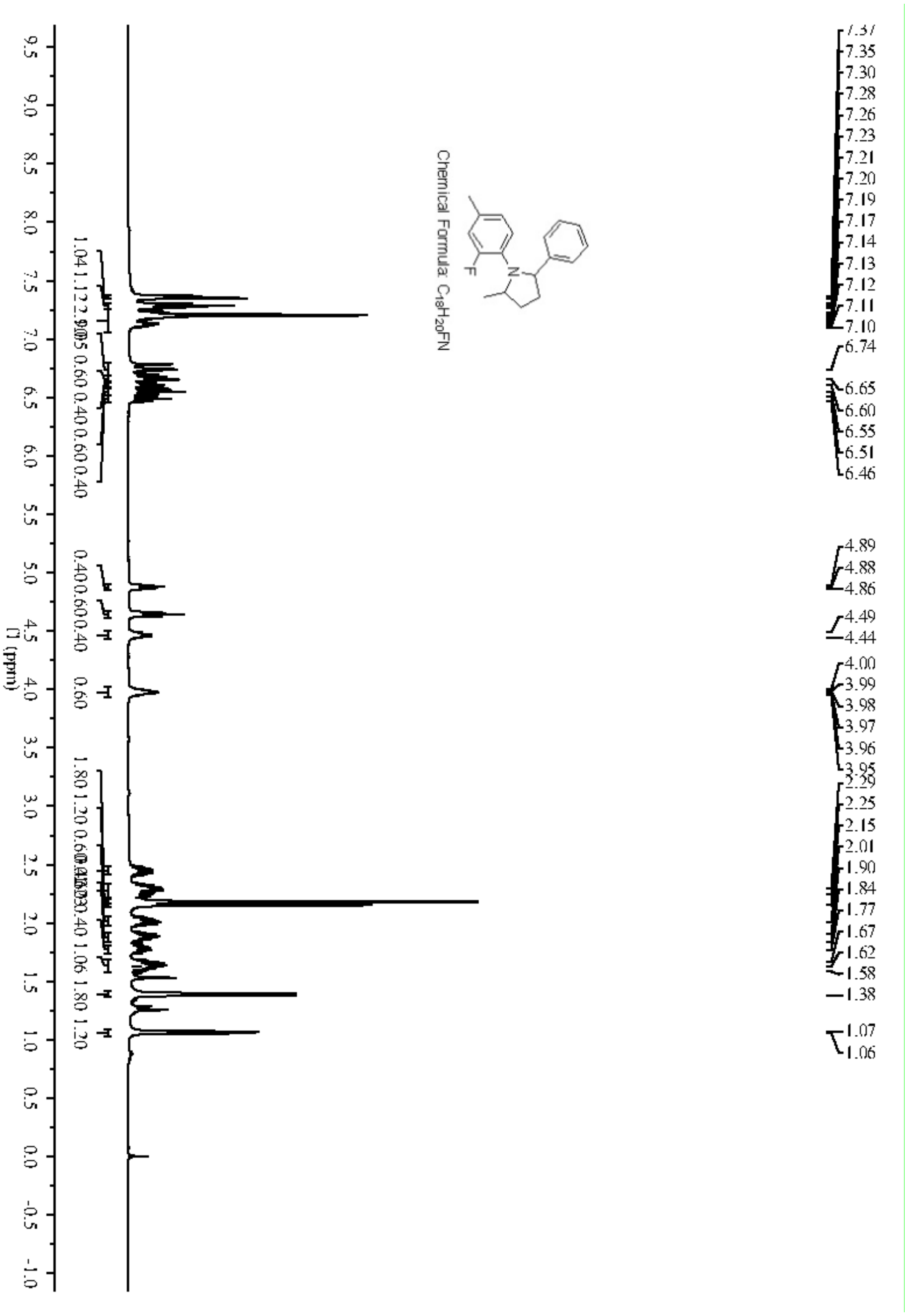

^{1}H NMR (400 MHz, $CDCl_3$) spectrum of **3b5**

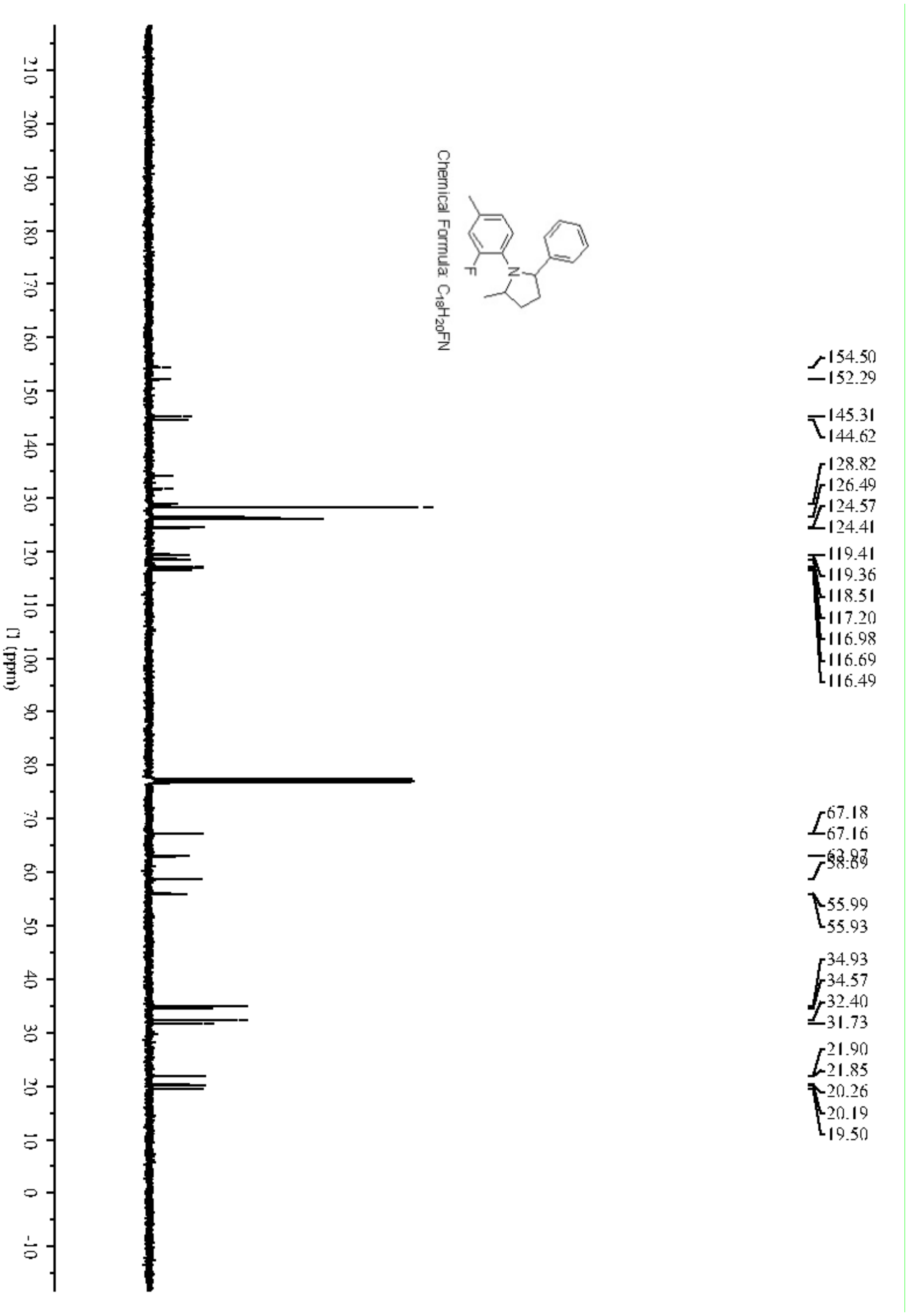

^{13}C NMR (101 MHz, $CDCl_3$) spectrum of **3b5**

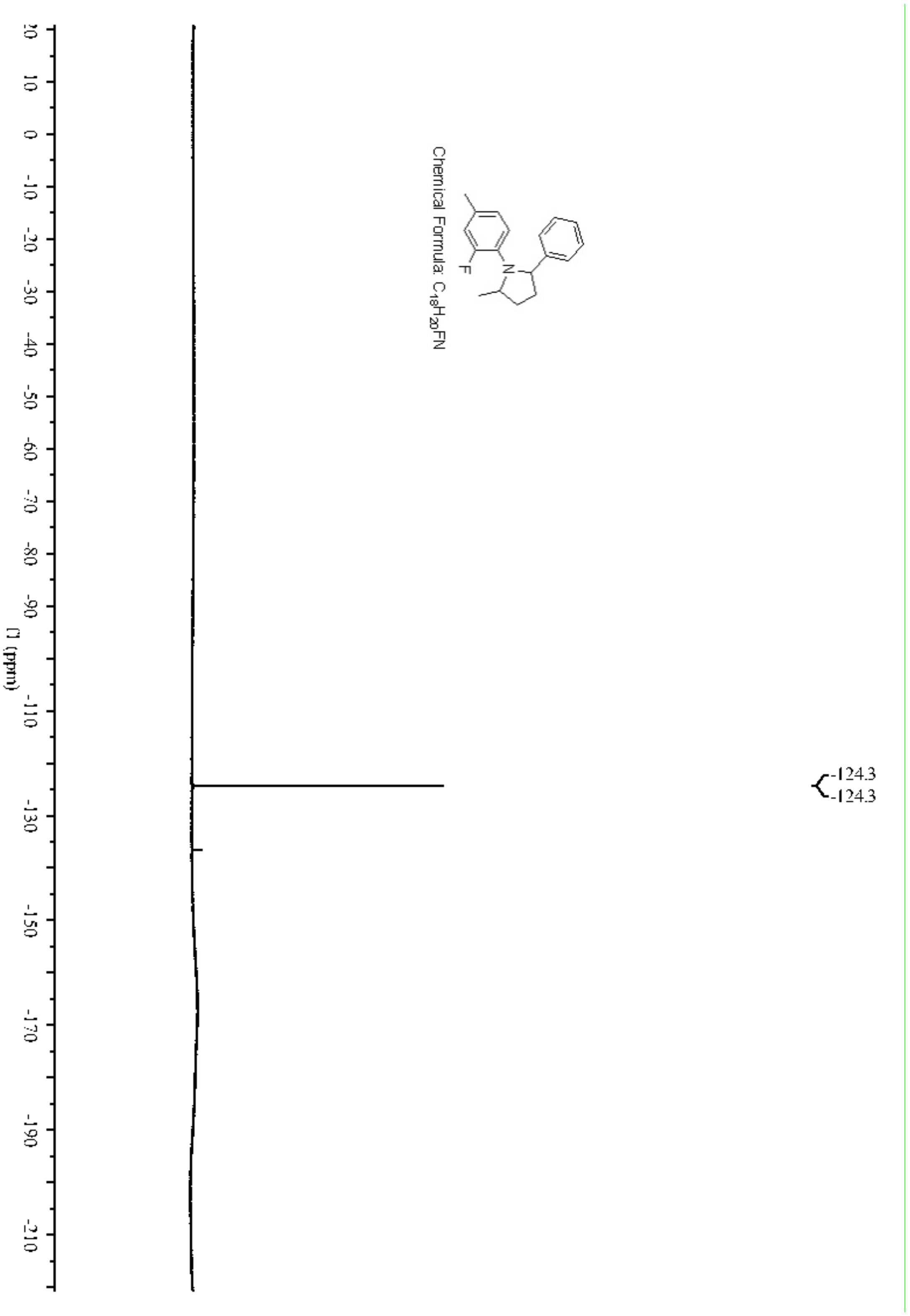

^{19}F NMR (377 MHz, $CDCl_3$) spectrum of **3b5**

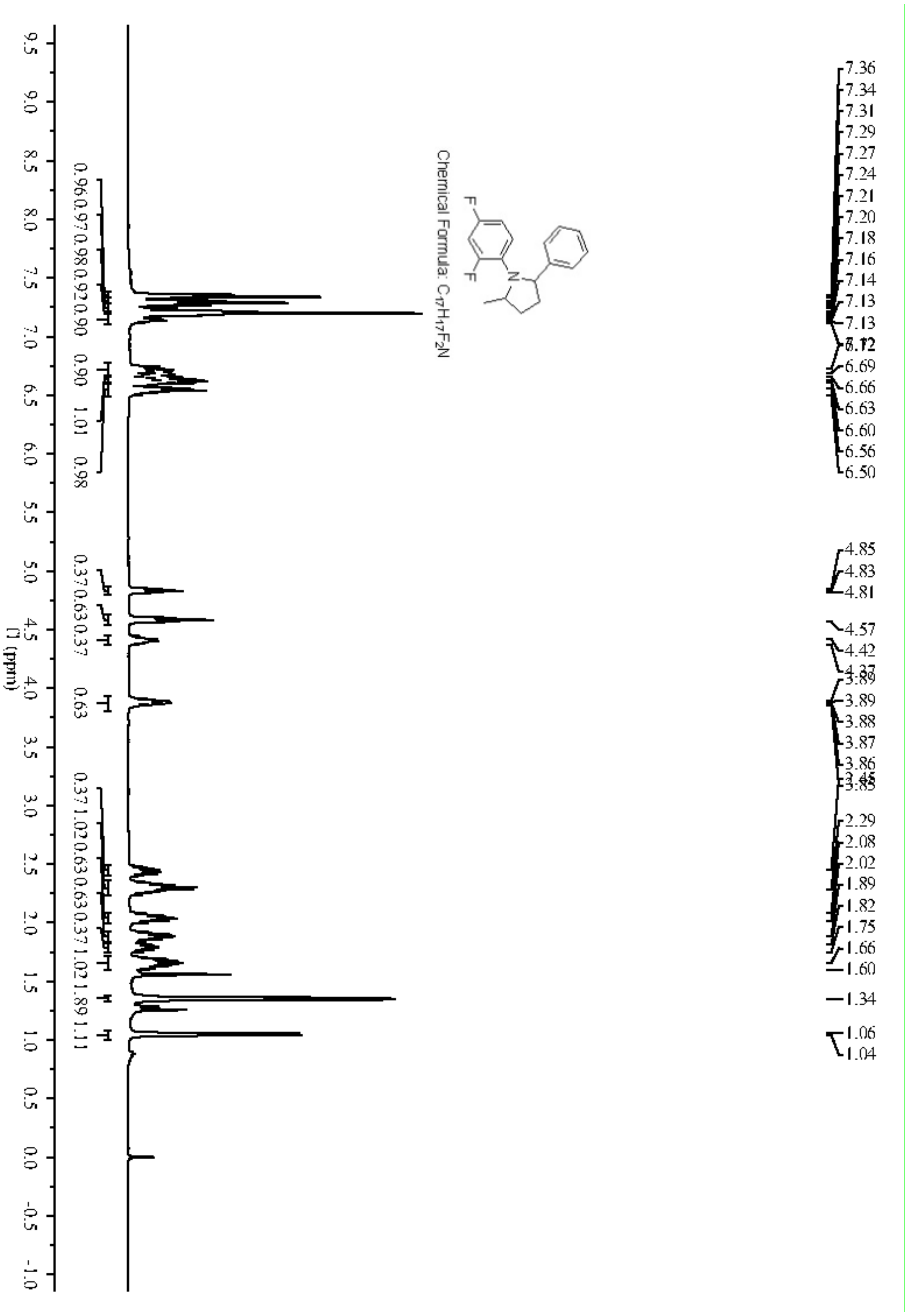

^{1}H NMR (400 MHz, $CDCl_3$) spectrum of **3b6**

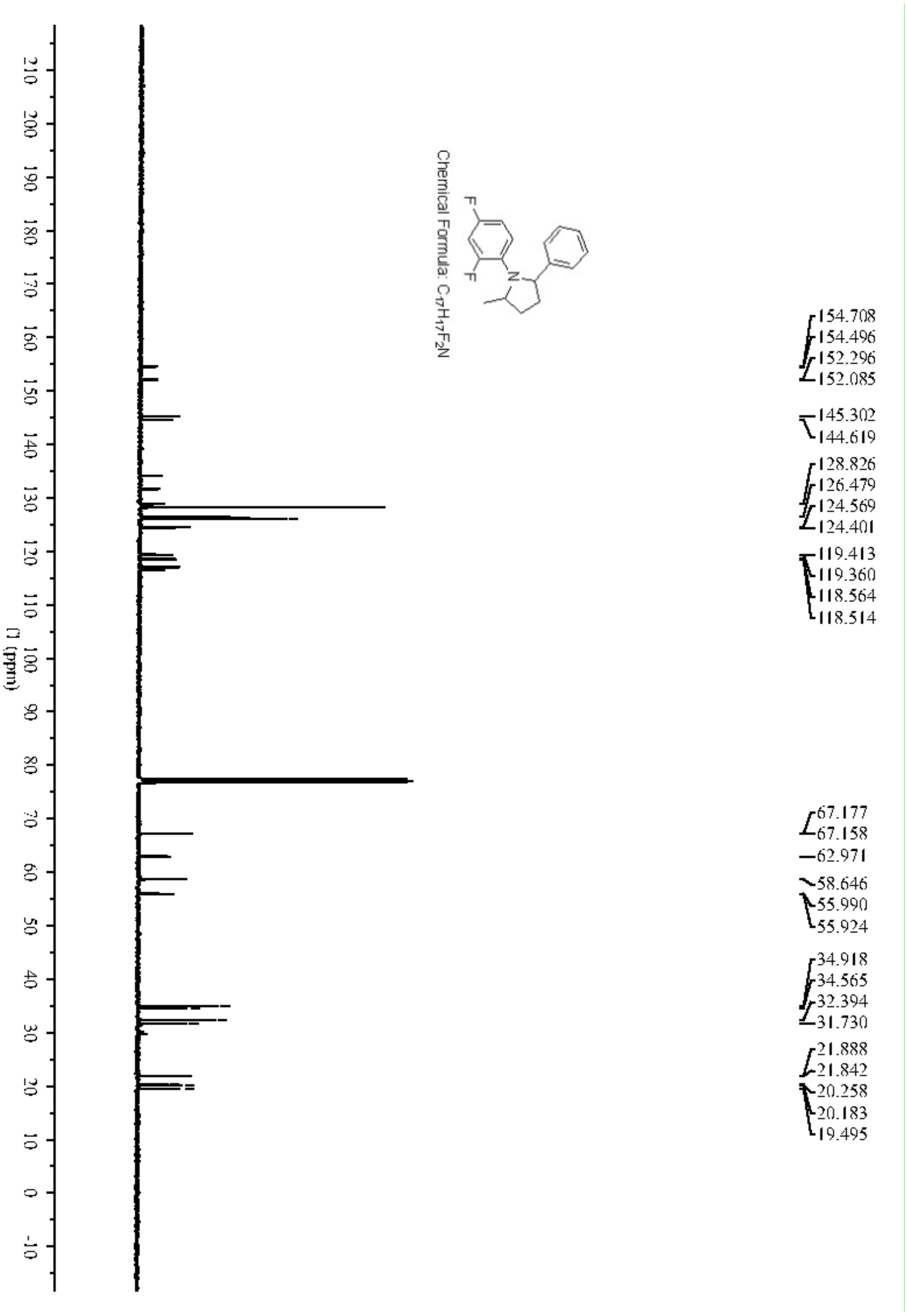

^{13}C NMR (101 MHz, $CDCl_3$) spectrum of **3b6**

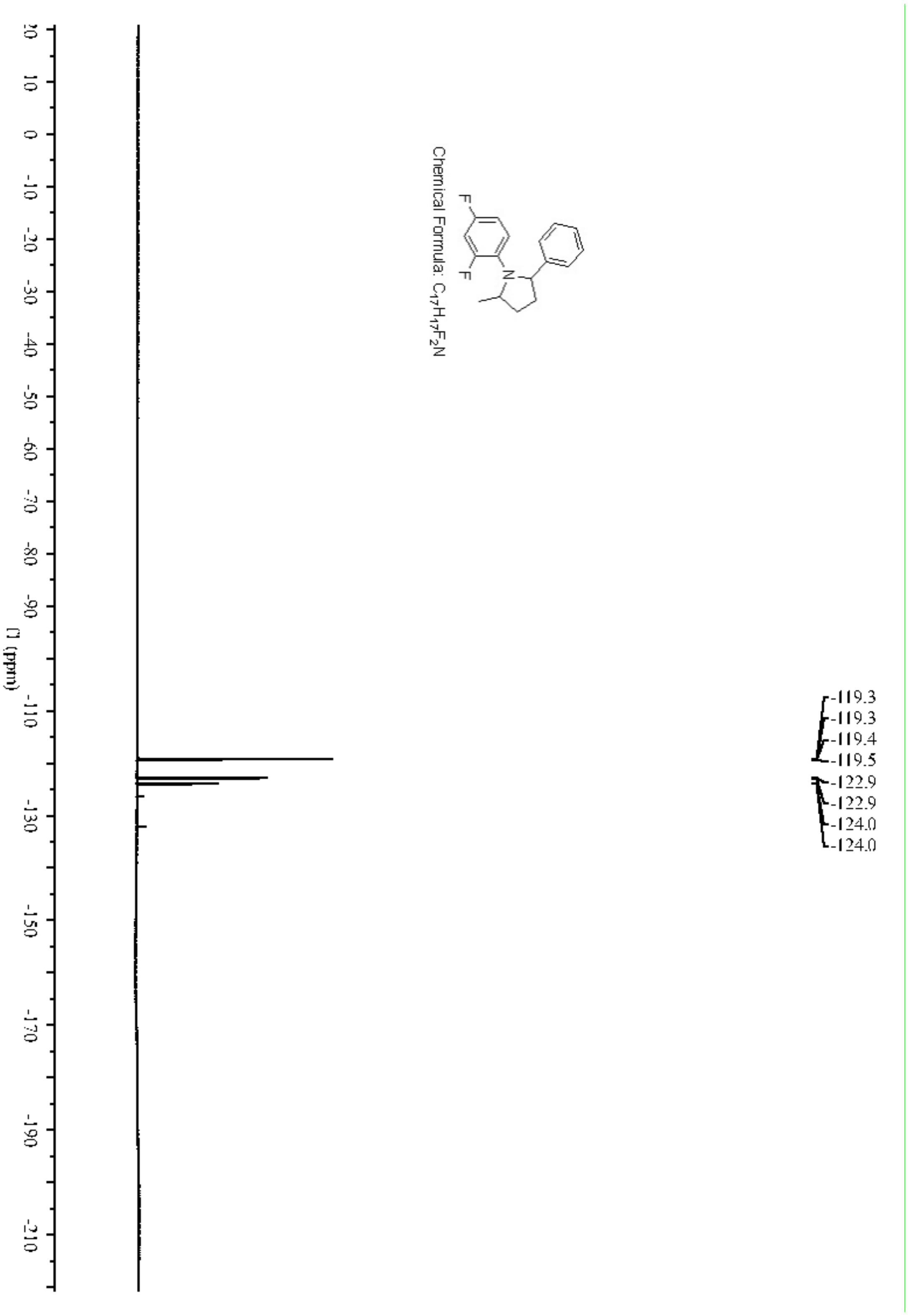

^{19}F NMR (377 MHz, $CDCl_3$) spectrum of **3b6**

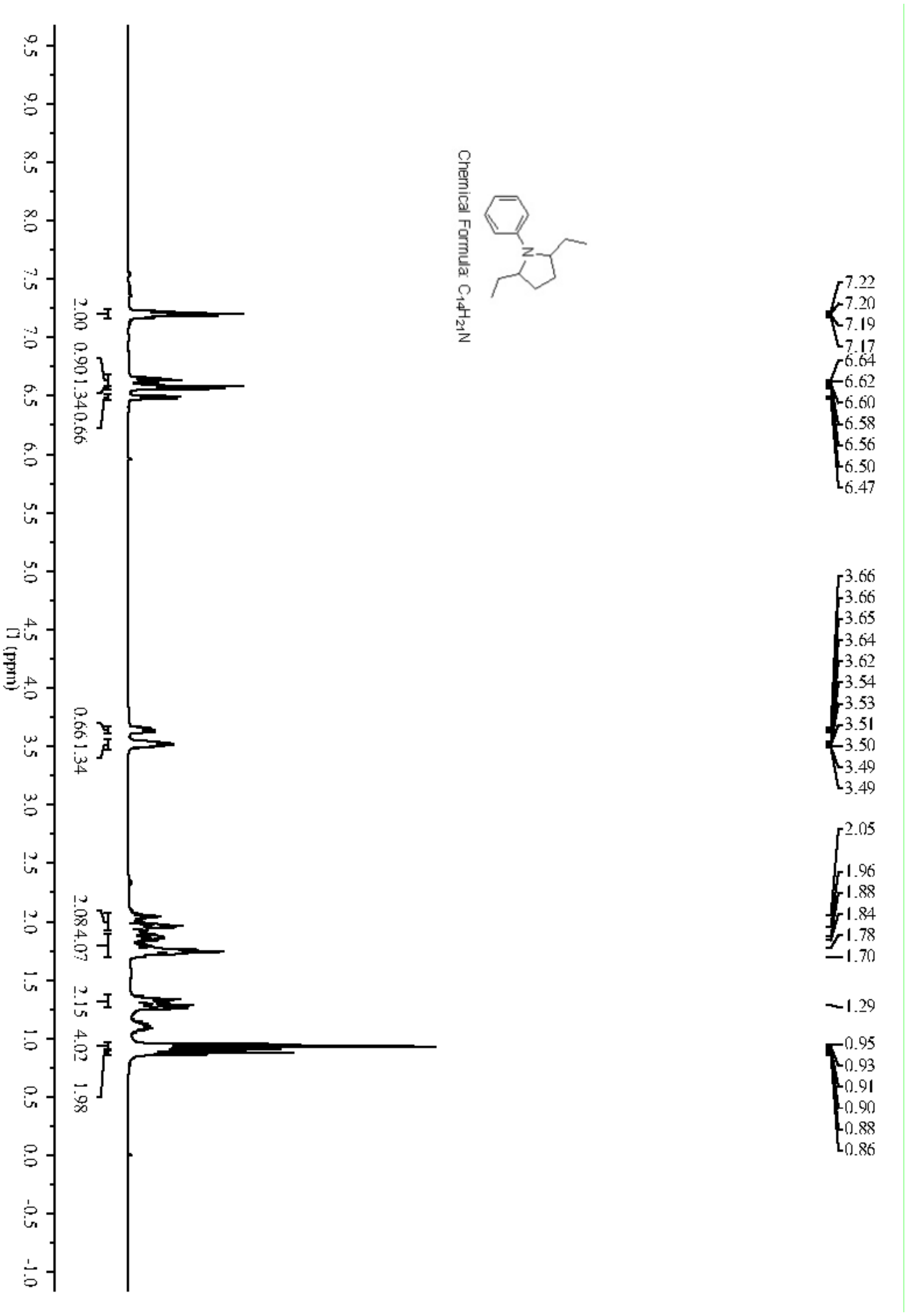

^{1}H NMR (400 MHz, $CDCl_3$) spectrum of **3c1**

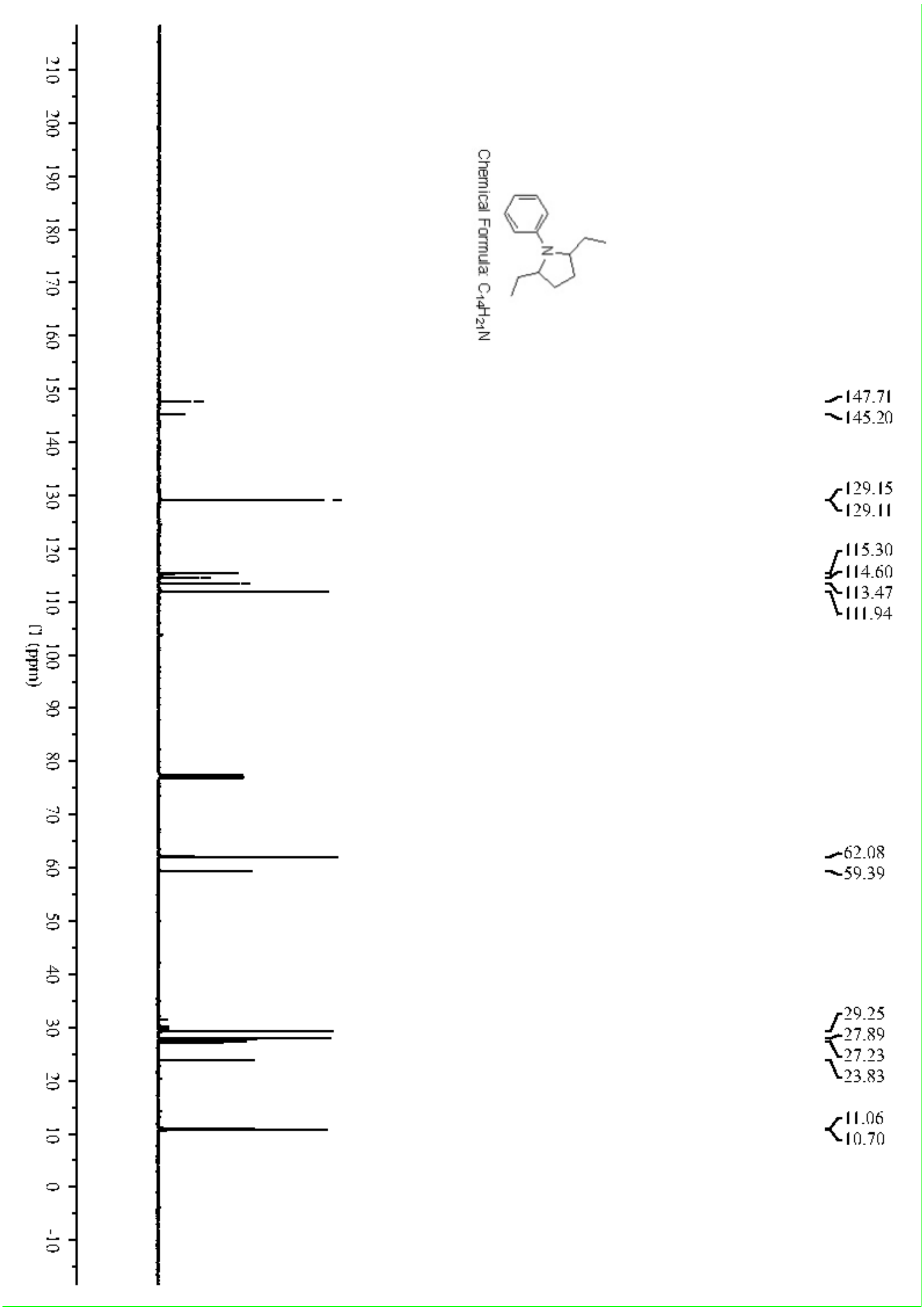

^{13}C NMR (101 MHz, $CDCl_3$) spectrum of **3c1**

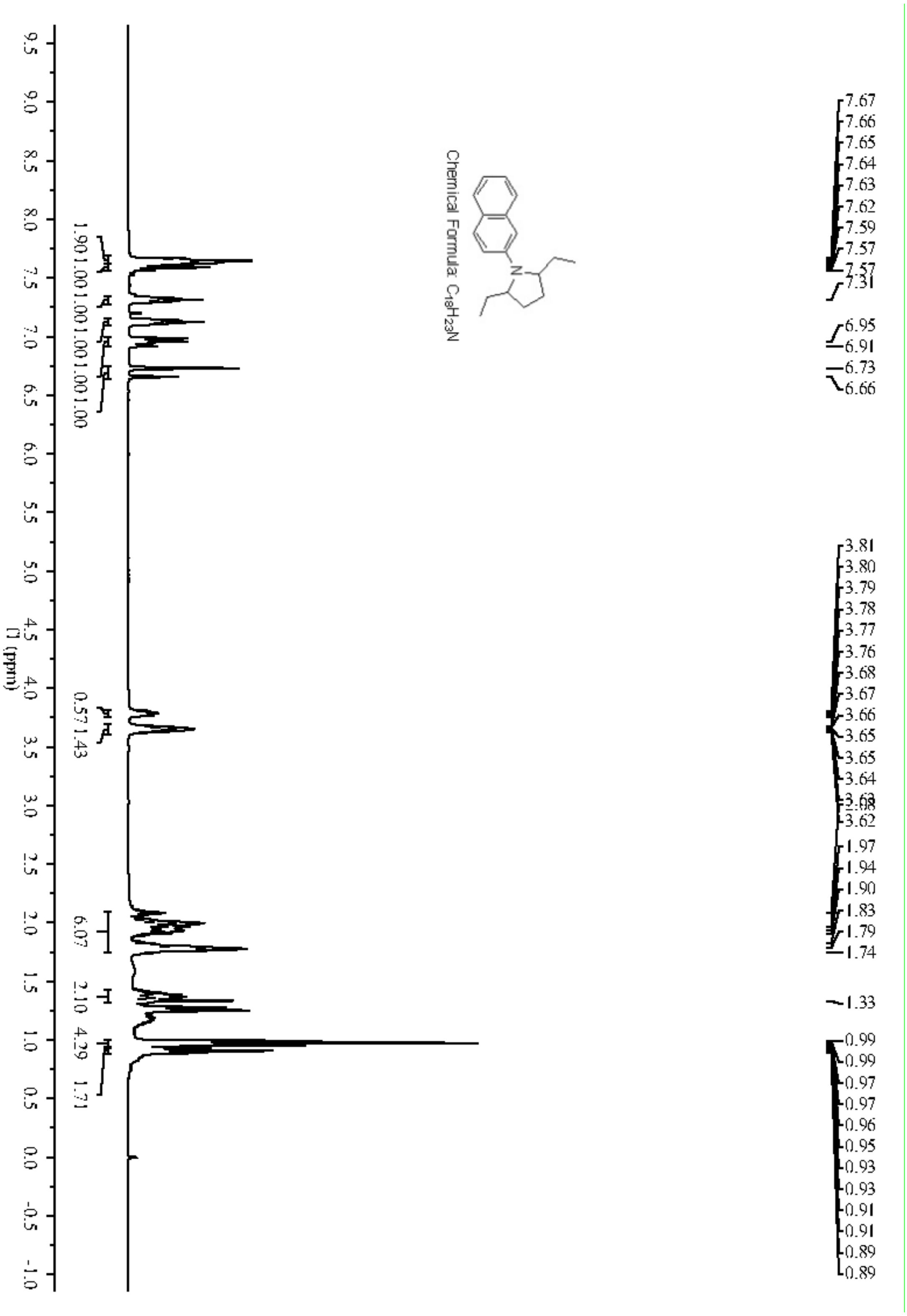

^{1}H NMR (400 MHz, $CDCl_3$) spectrum of **3c2**

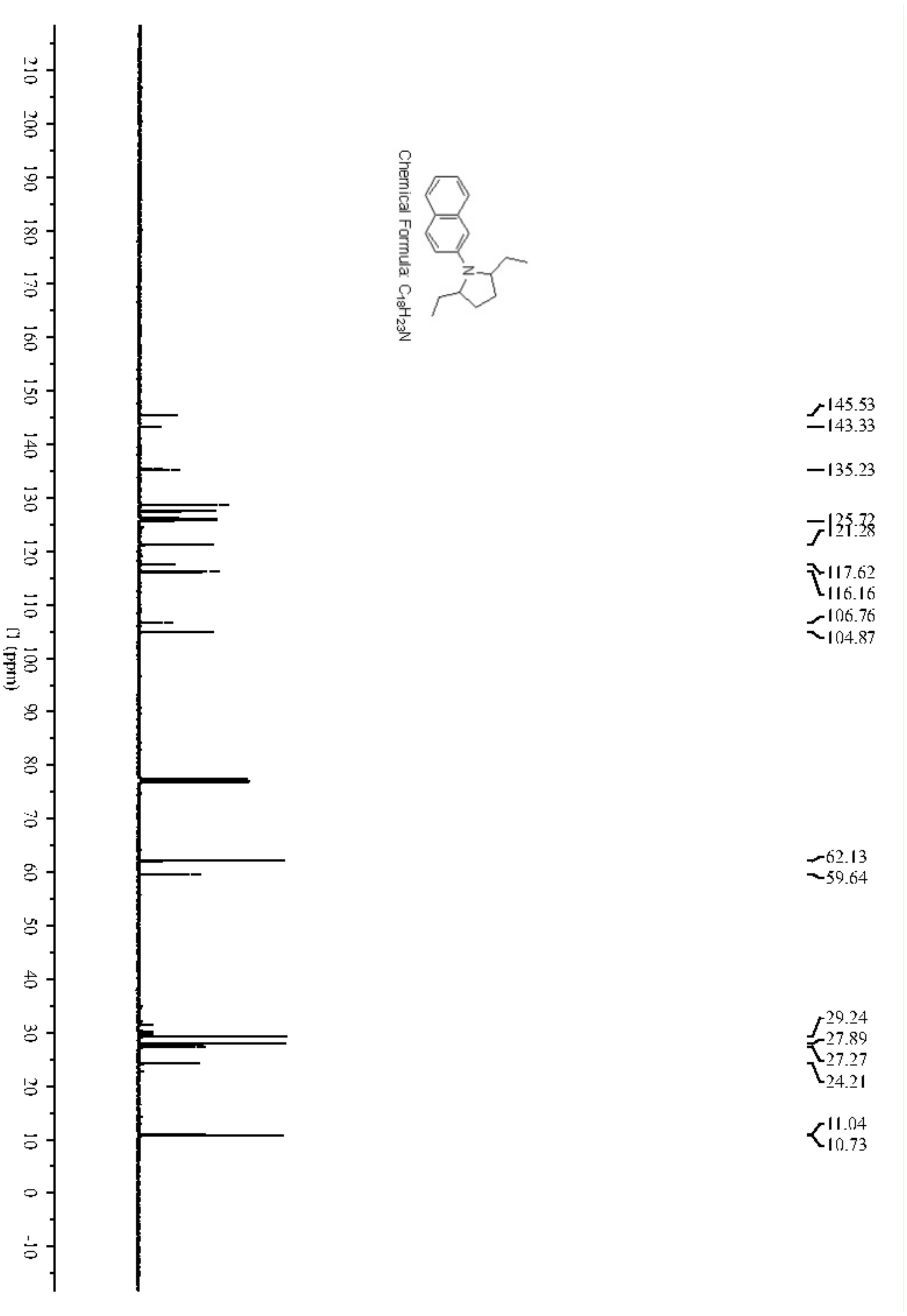

^{13}C NMR (101 MHz, $CDCl_3$) spectrum of **3c2**

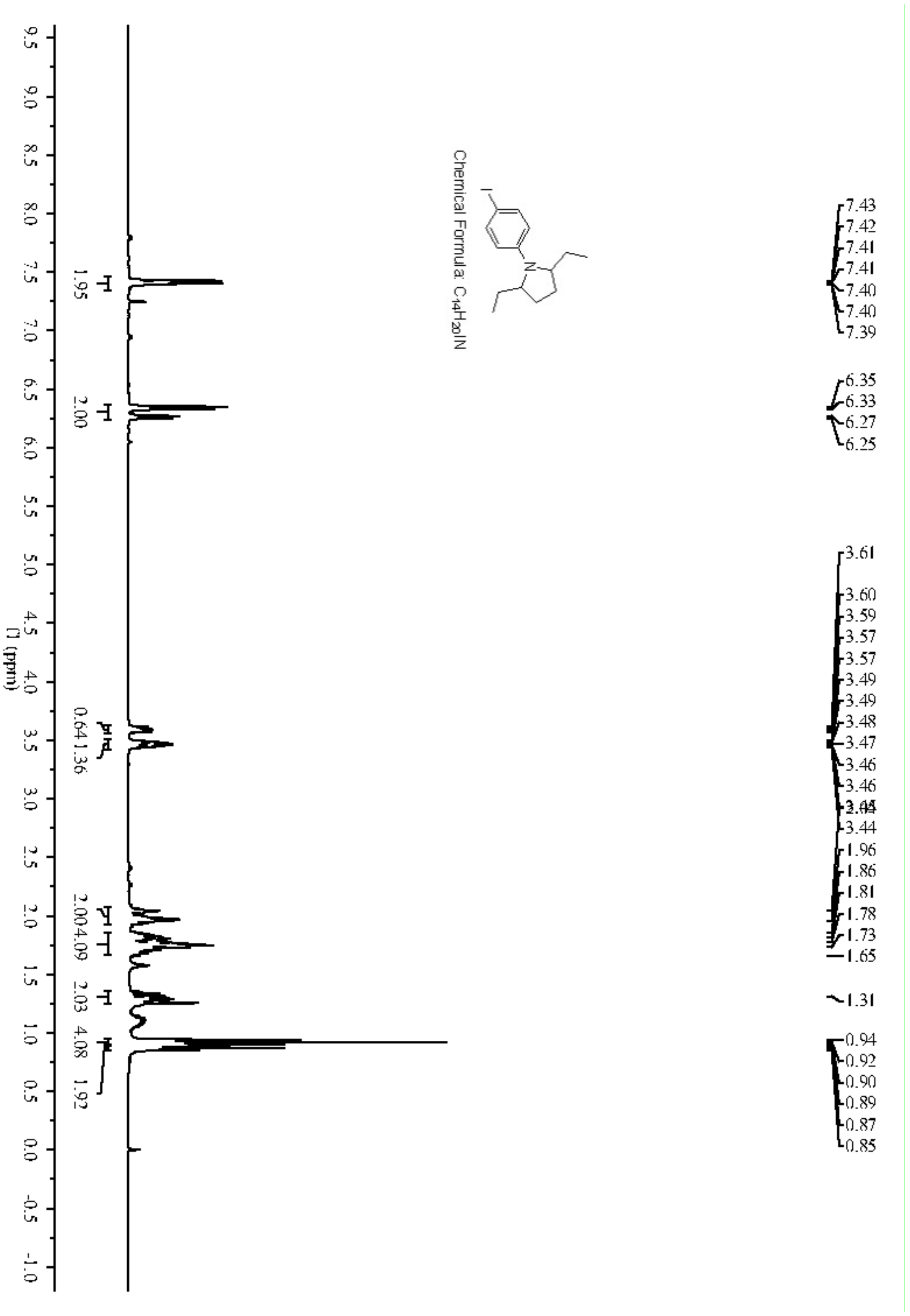

^{1}H NMR (400 MHz, CDCl3) spectrum of **3c3**

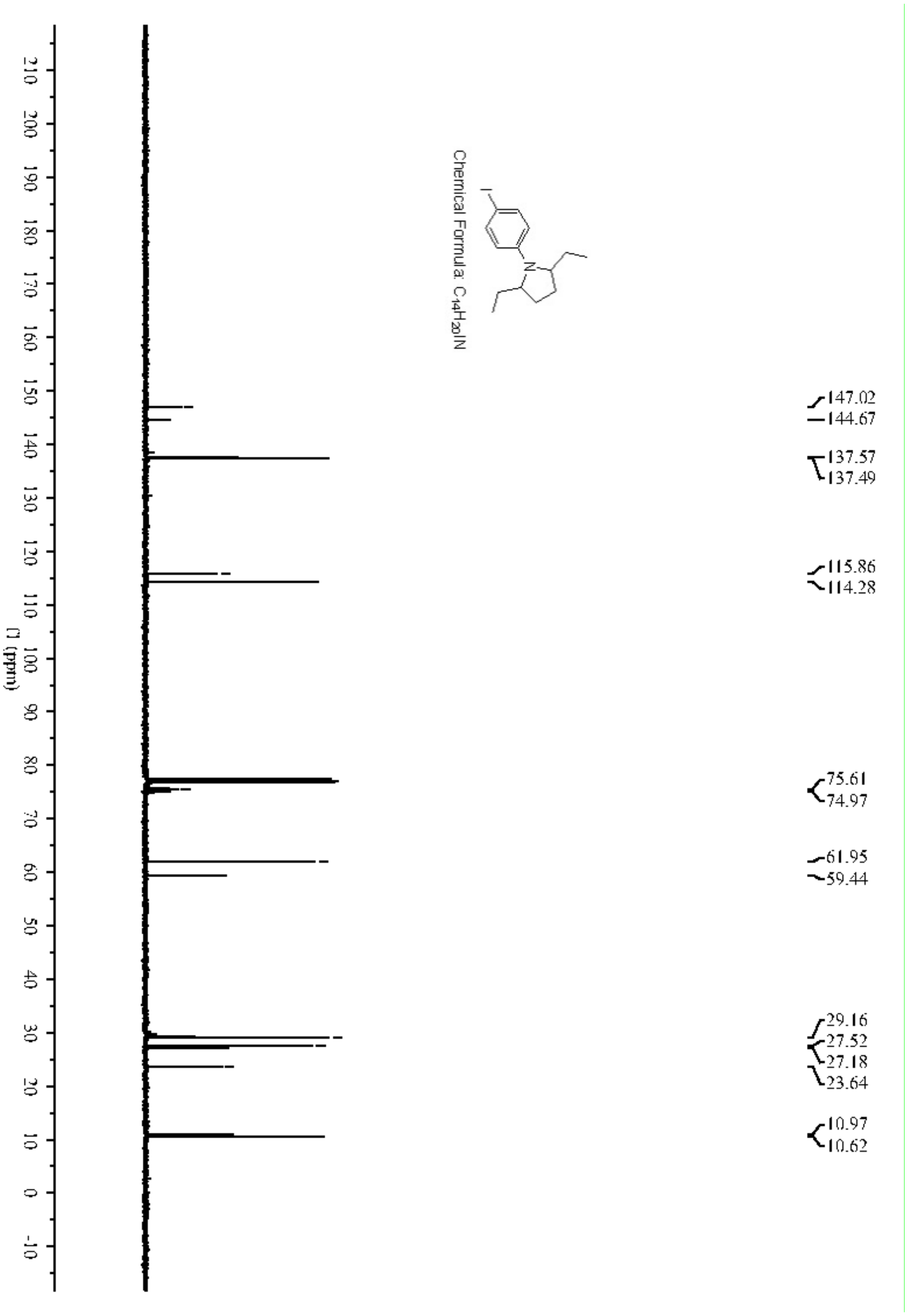

^{13}C NMR (101 MHz, $CDCl_3$) spectrum of **3c3**

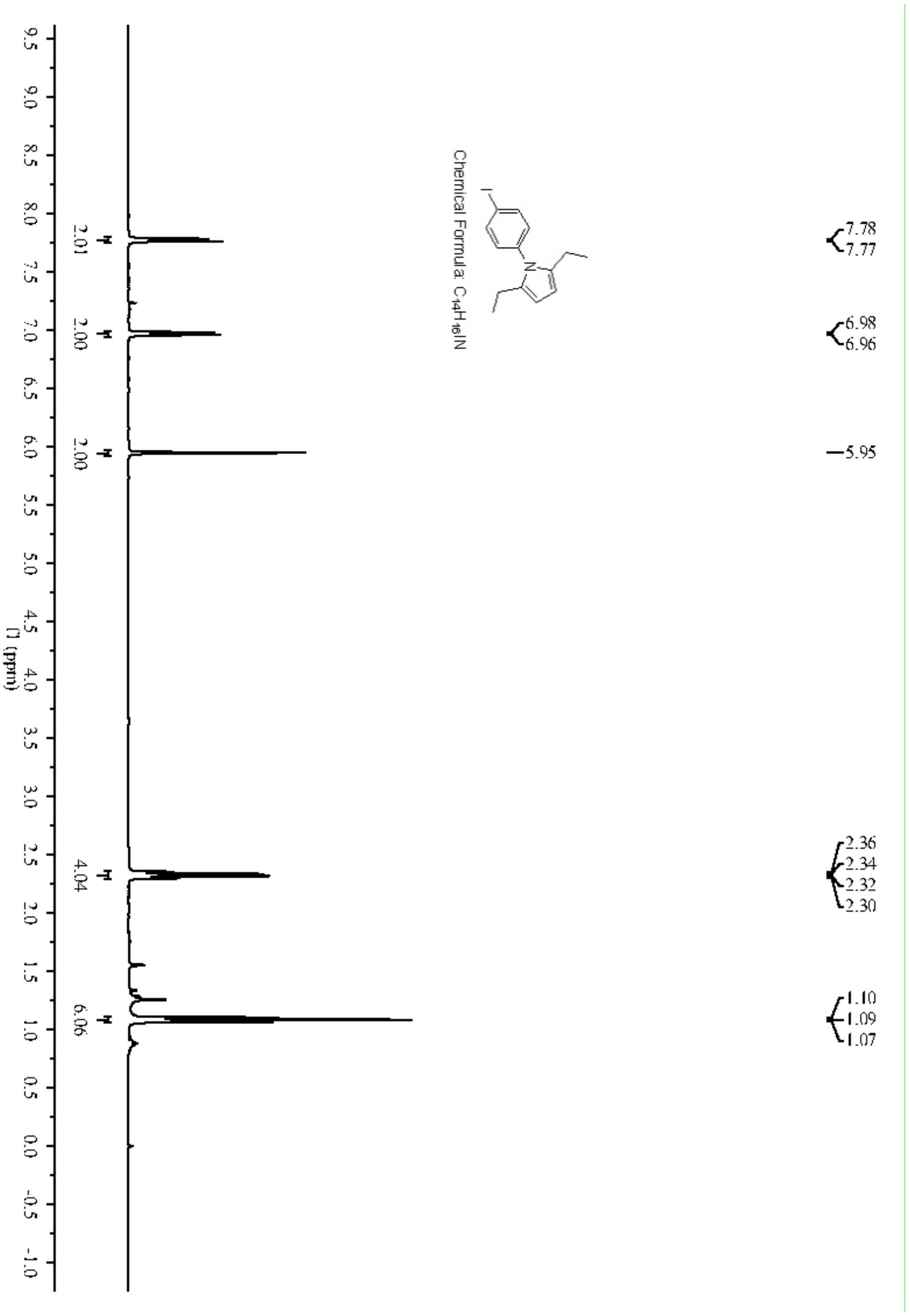

^{1}H NMR (400 MHz, $CDCl_3$) spectrum of **3c3**’

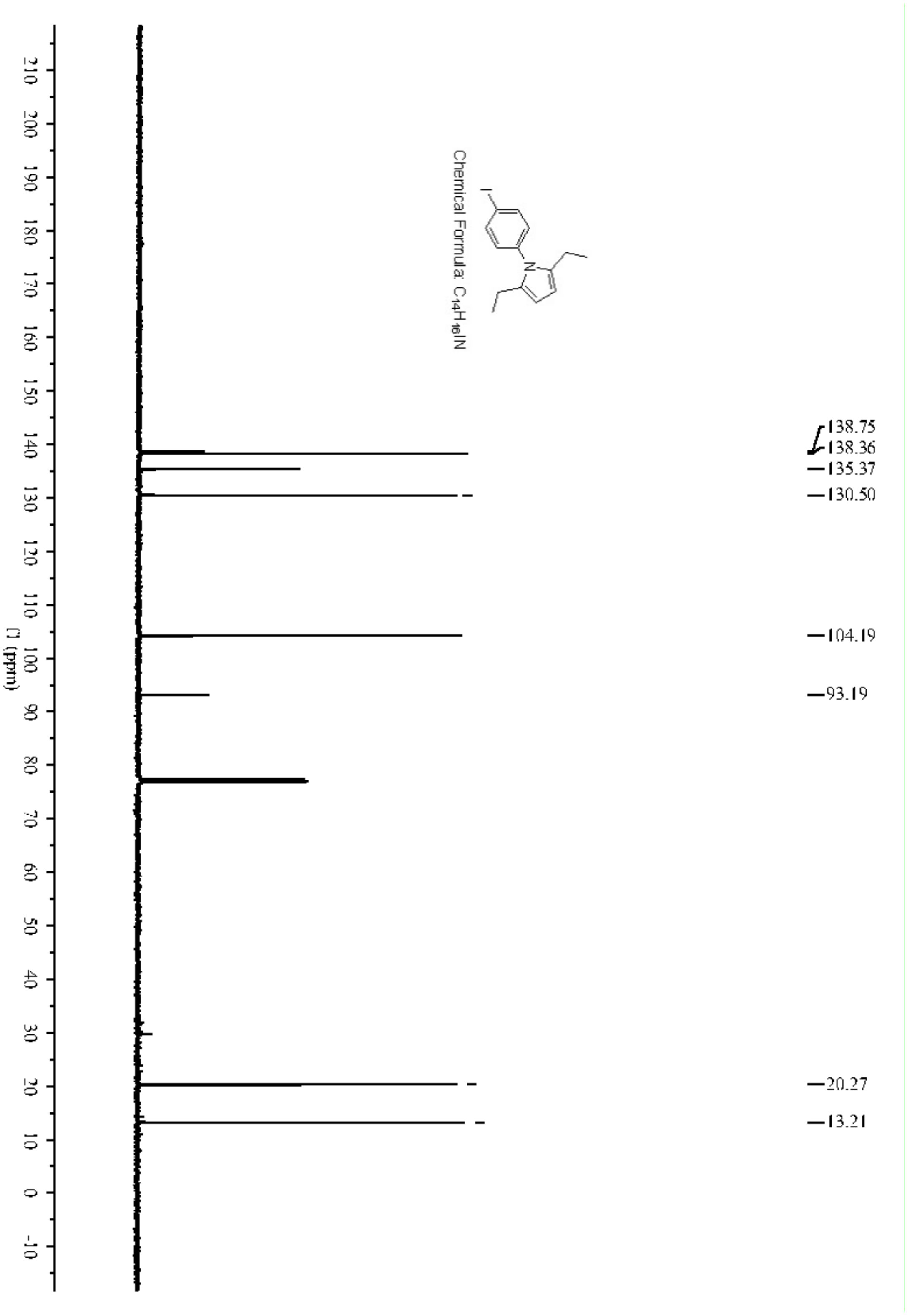

^{13}C NMR (101 MHz, $CDCl_3$) spectrum of **3c3'**

G. X-ray crystal structure of compound 3b4.

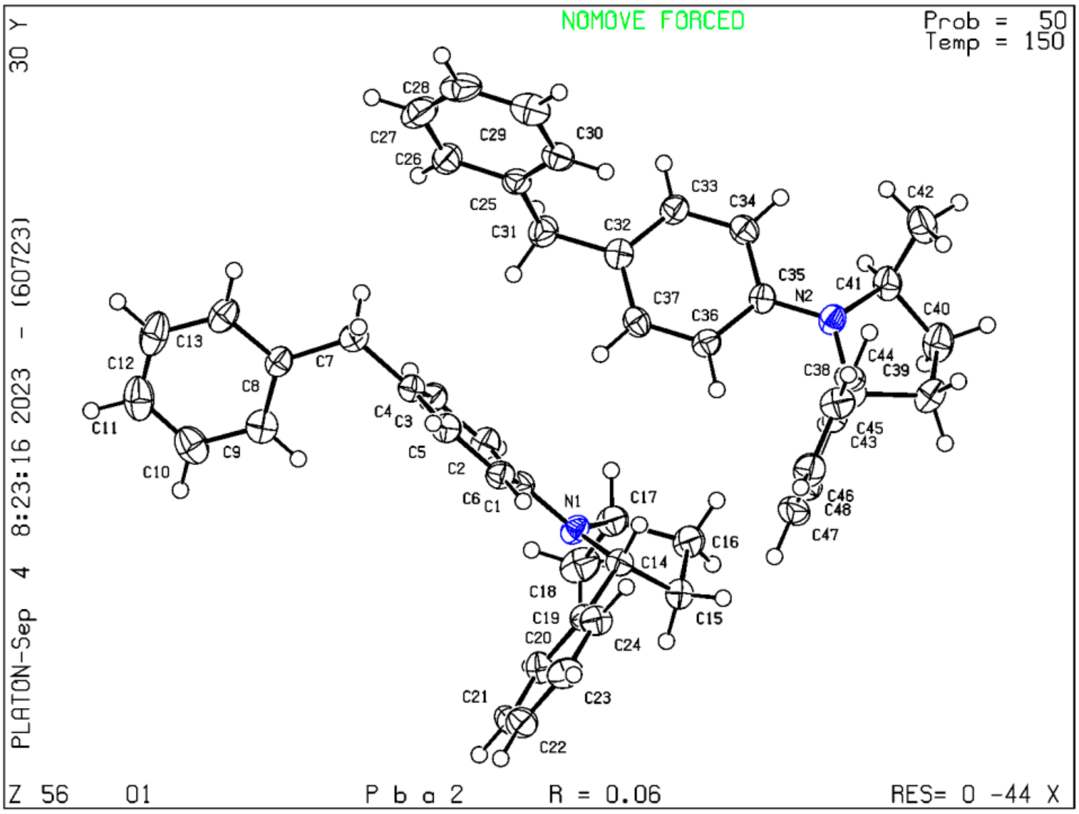

Figure S1 X-ray crystal structures of compound **3b4**.

H. Crystal structure determination

**Table S1 Crystal data and structure refinement for 3b4.**	
Identification code	3bv
Empirical formula	$C_{24}H_{25}N$
Formula weight	327.45
Temperature/K	150.00(10)
Crystal system	orthorhombic
Space group	Pba2
a/Å	45.643(3)
b/Å	10.5128(11)
c/Å	7.6049(4)
α/°	90
β/°	90
γ/°	90
Volume/Å^3	3649.1(5)
Z	8
ρ_{calc}g/cm^3	1.192
μ/mm^{-1}	0.515
F(000)	1408.0
Crystal size/mm^3	0.16 × 0.12 × 0.1
Radiation	Cu Kα (λ = 1.54184)
2Θ range for data collection/°	7.748 to 147.568
Index ranges	$-56 \leq h \leq 20$, $-13 \leq k \leq 9$, $-9 \leq l \leq 5$
Reflections collected	9021
Independent reflections	4665 [R_{int} = 0.0548, R_{sigma} = 0.0656]
Data/restraints/parameters	4665/1/453
Goodness-of-fit on F^2	1.034
Final R indexes [I>=2σ (I)]	R_1 = 0.0611, wR_2 = 0.1544
Final R indexes [all data]	R_1 = 0.0743, wR_2 = 0.1681
Largest diff. peak/hole / e Å^{-3}	0.29/-0.27
Flack parameter	-1.3(10)

I. IR Spectra

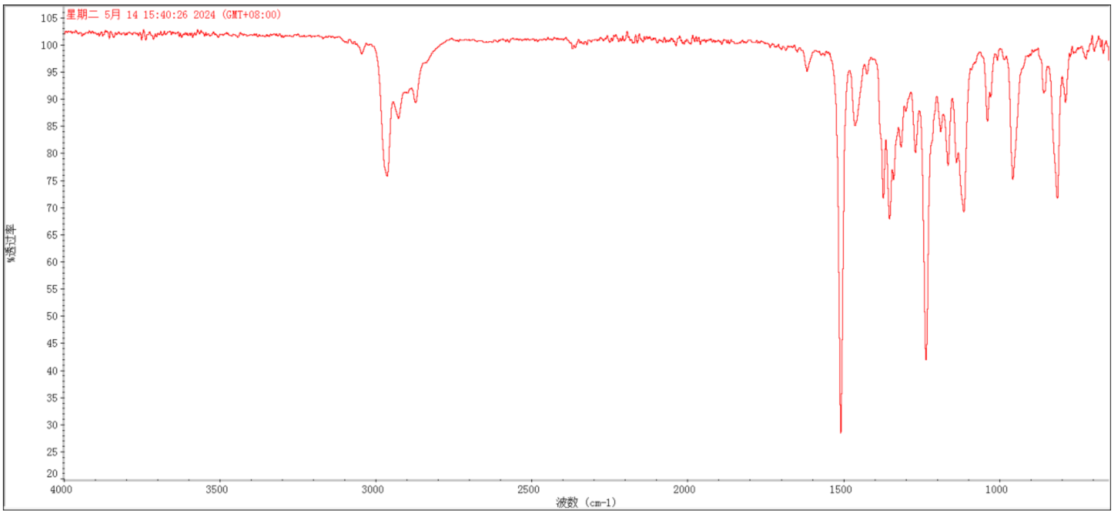

IR Spectra of **3a4**

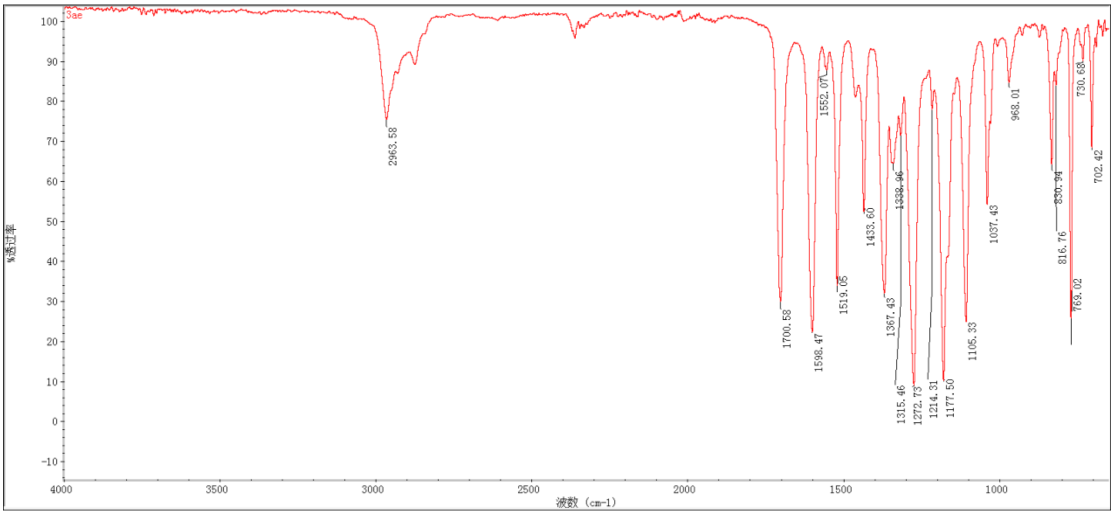

IR Spectra of **3a5**

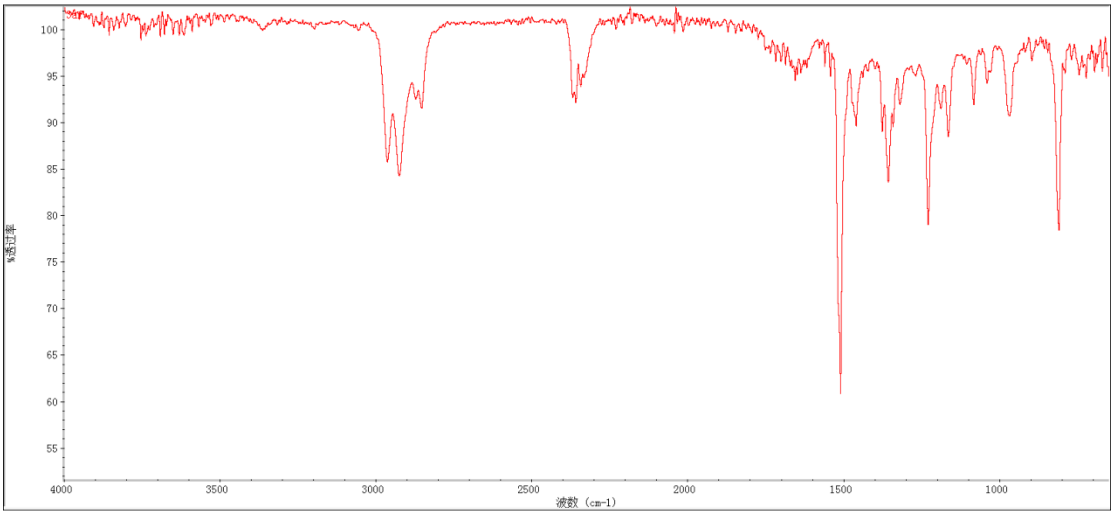

IR Spectra of **3a6**

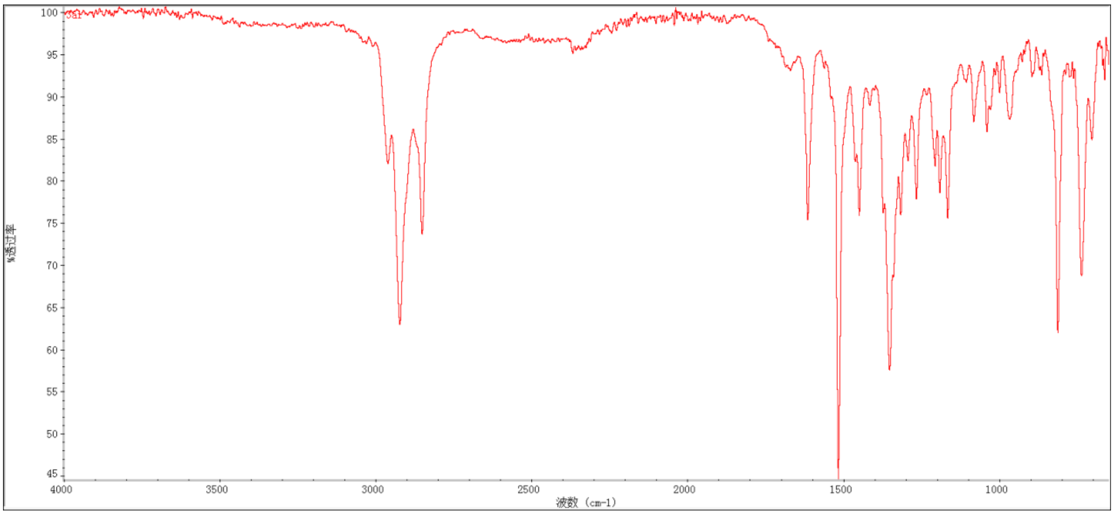

IR Spectra of **3a9**

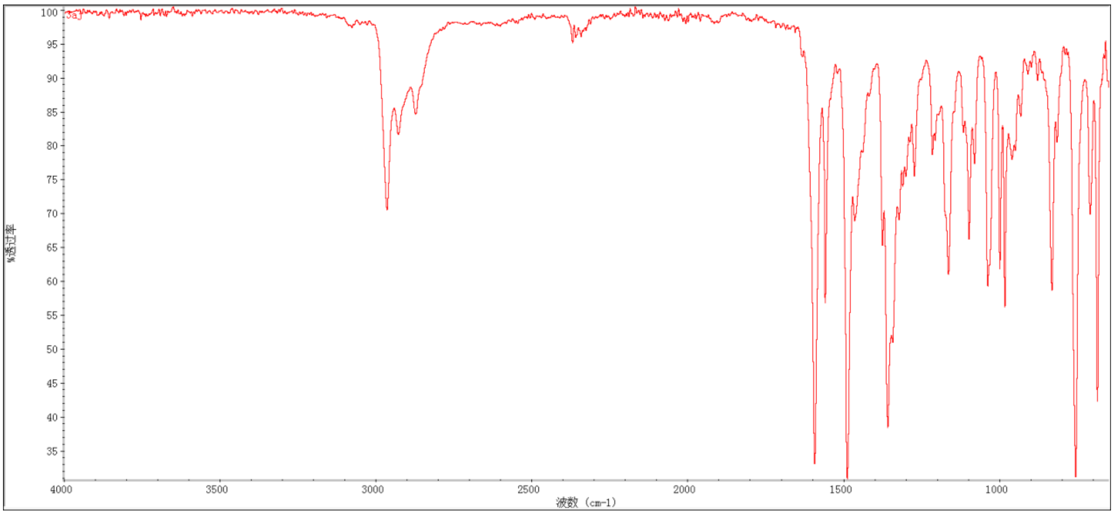

IR Spectra of **3a10**

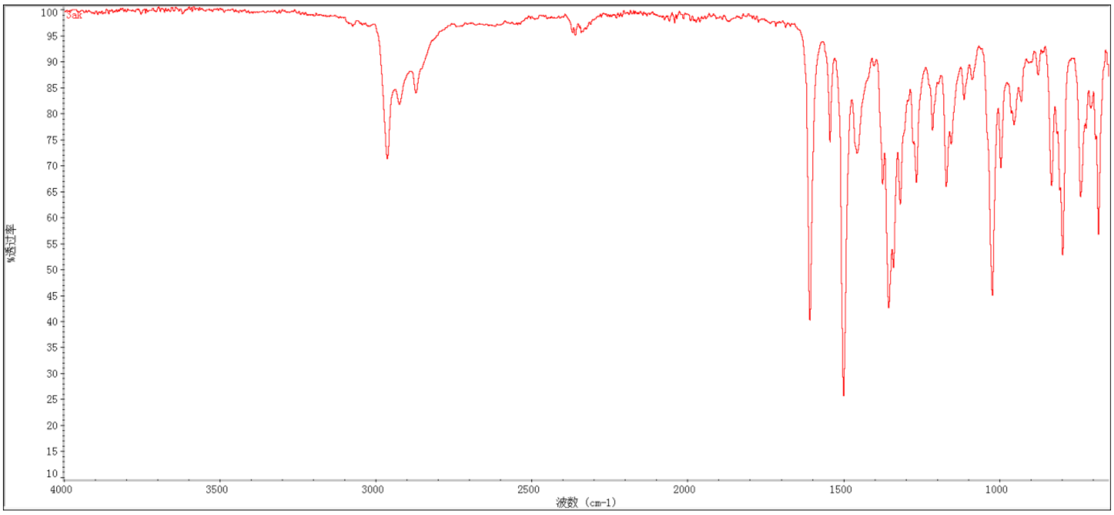

IR Spectra of **3a11**

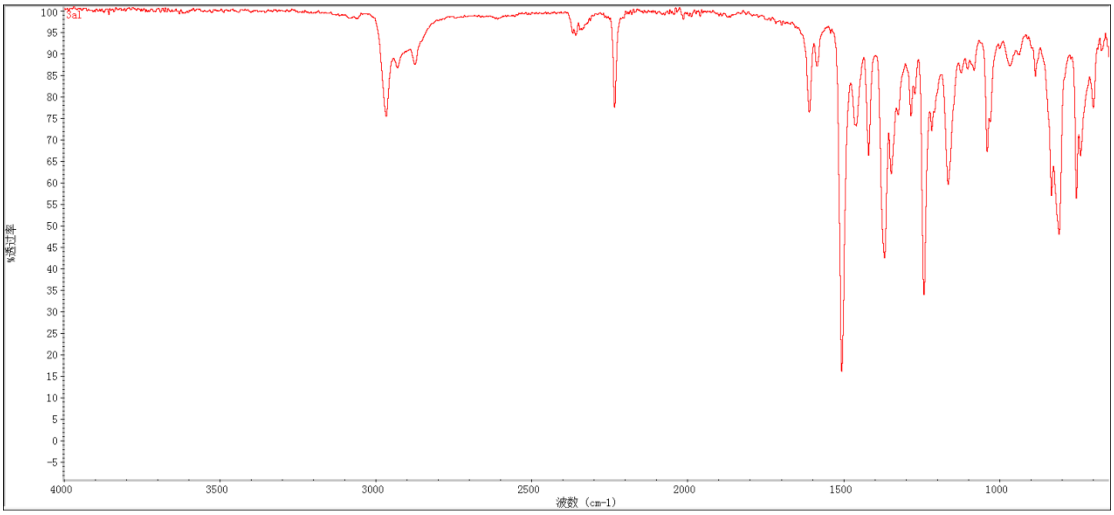

IR Spectra of **3a12**

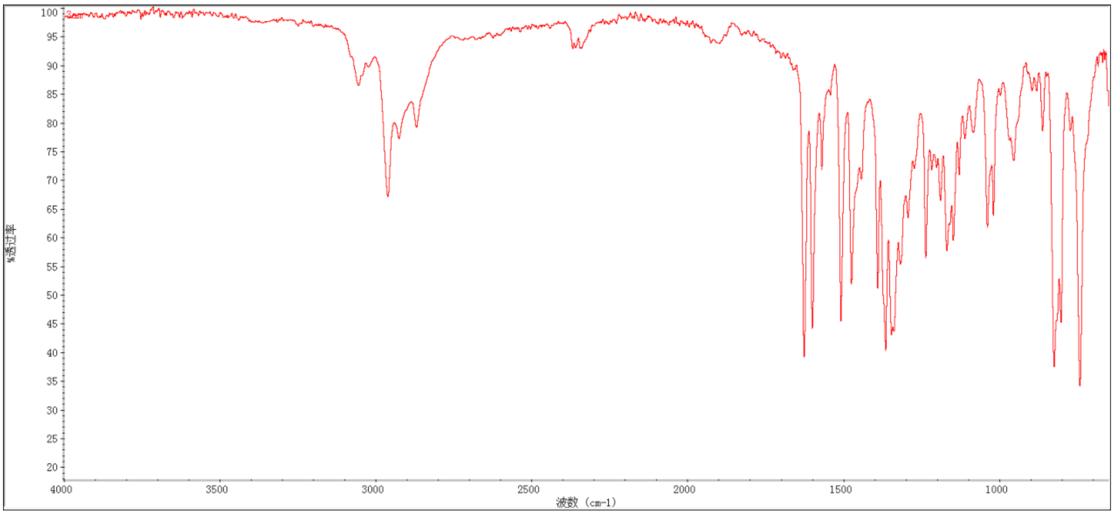

IR Spectra of **3a13**

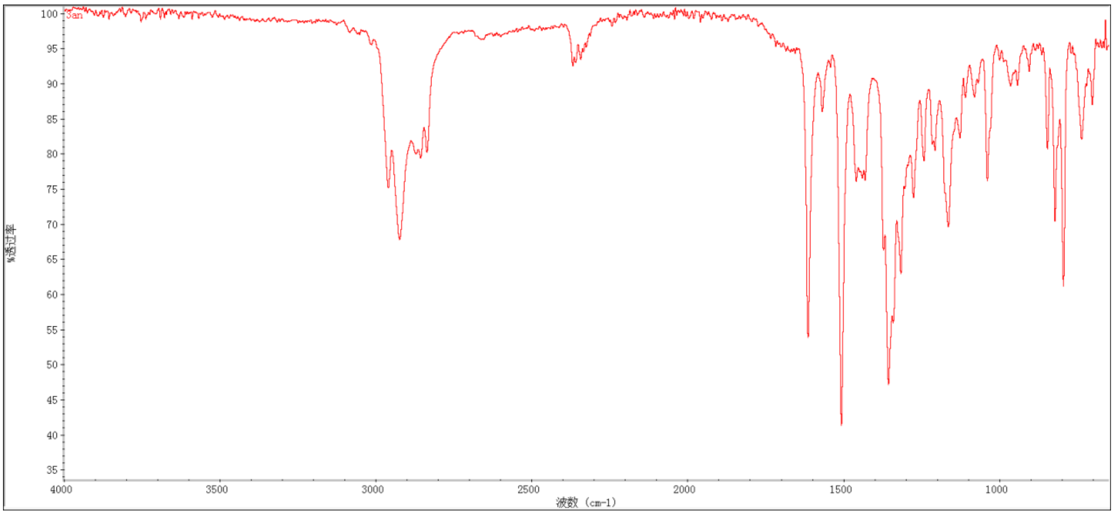

IR Spectra of **3a14**

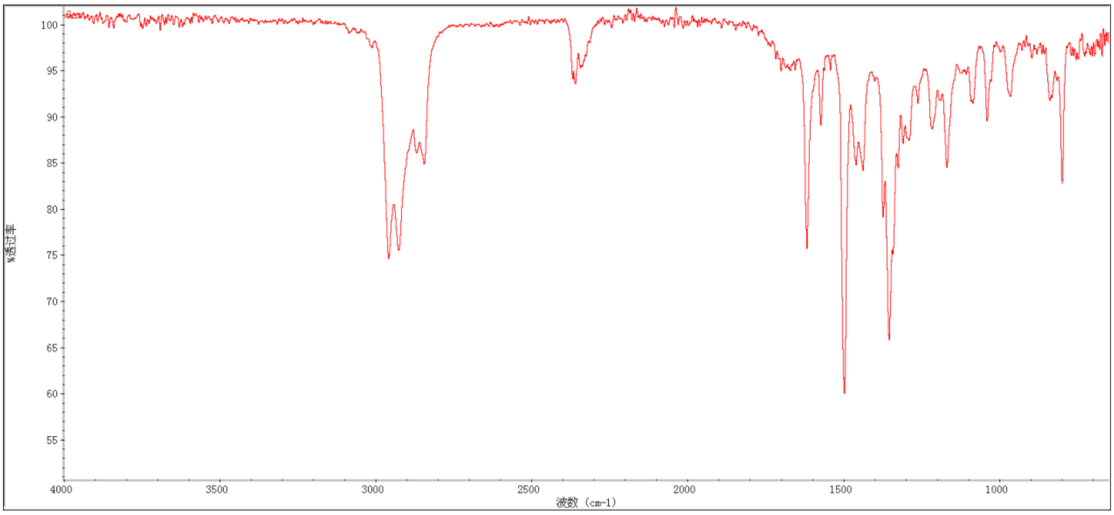

IR Spectra of **3a15**

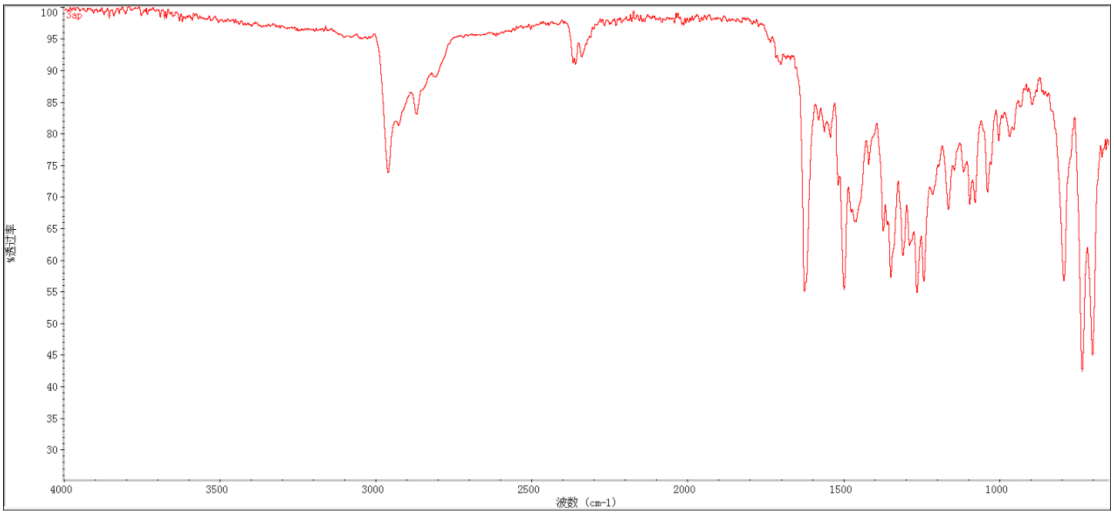

IR Spectra of **3a16**

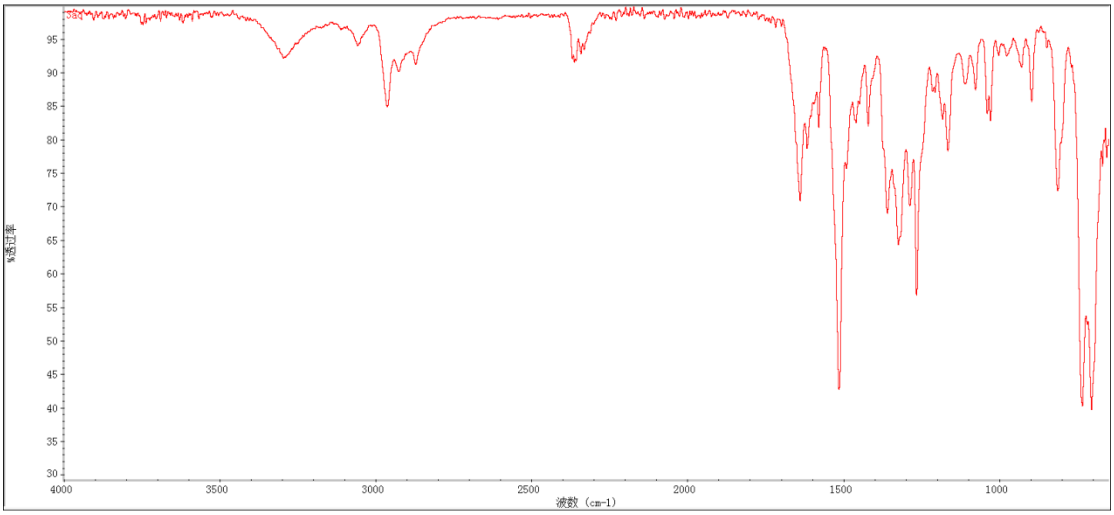

IR Spectra of **3a17**

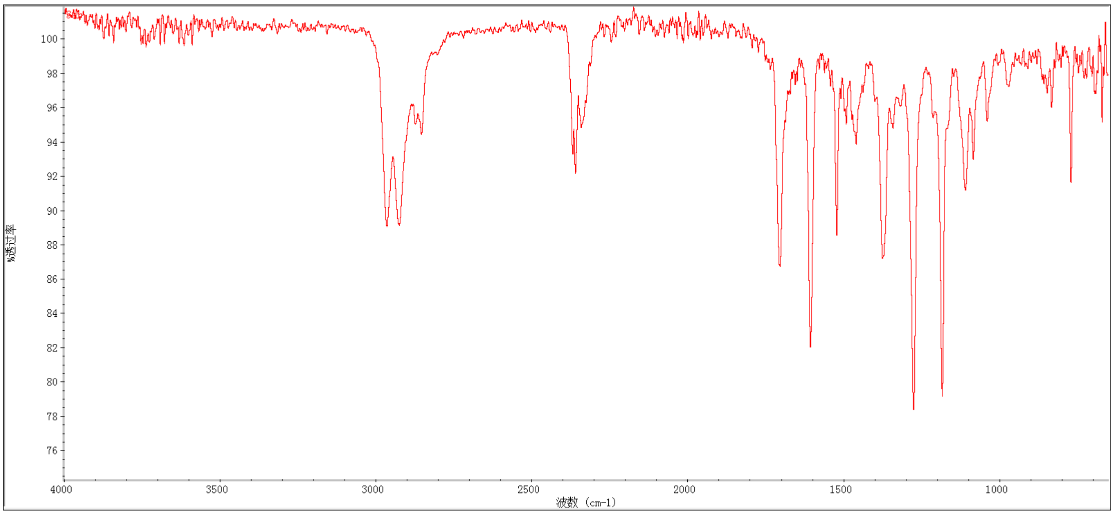

IR Spectra of **3a18**

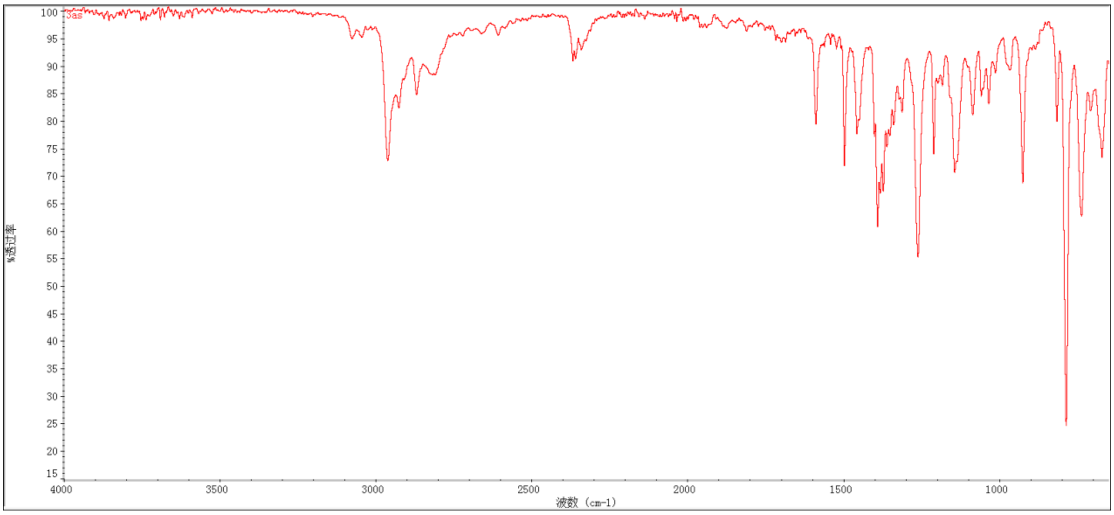

IR Spectra of **3a19**

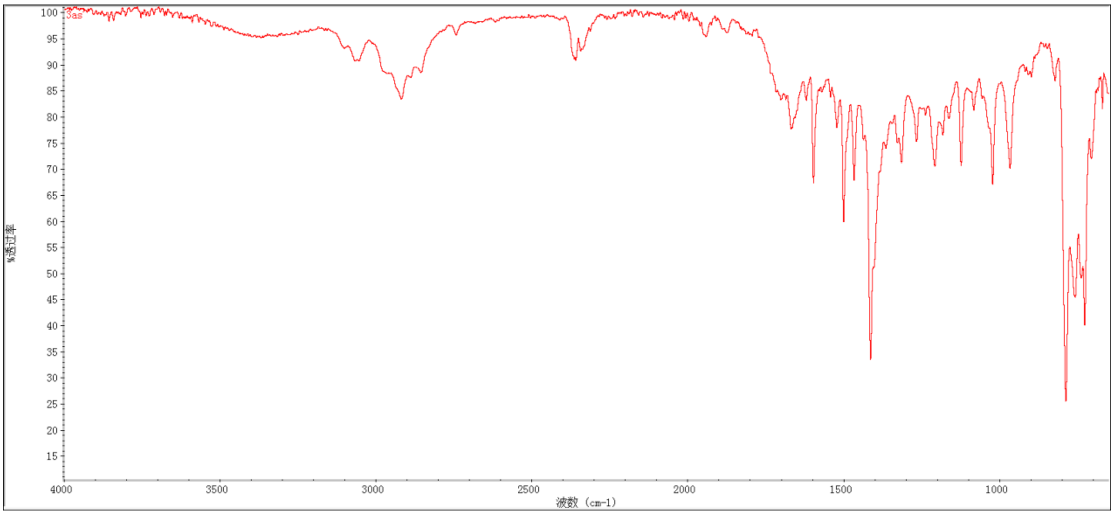

IR Spectra of **3a19’**

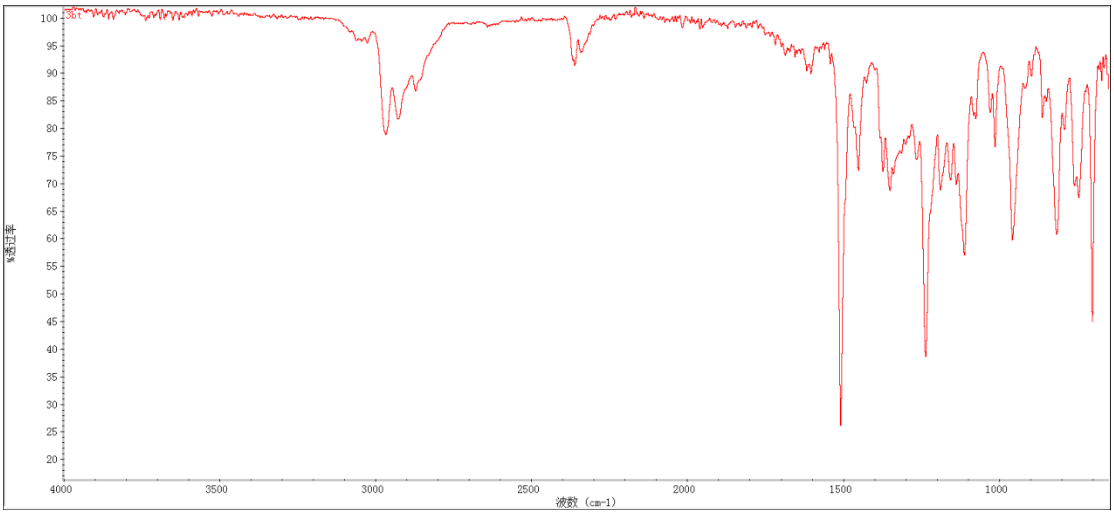

IR Spectra of **3b2**

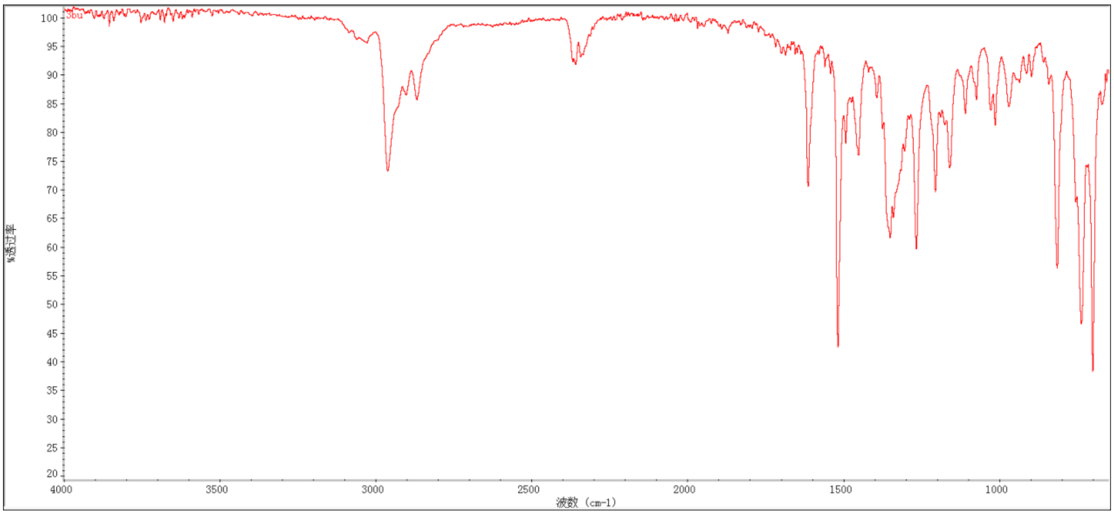

IR Spectra of **3b3**

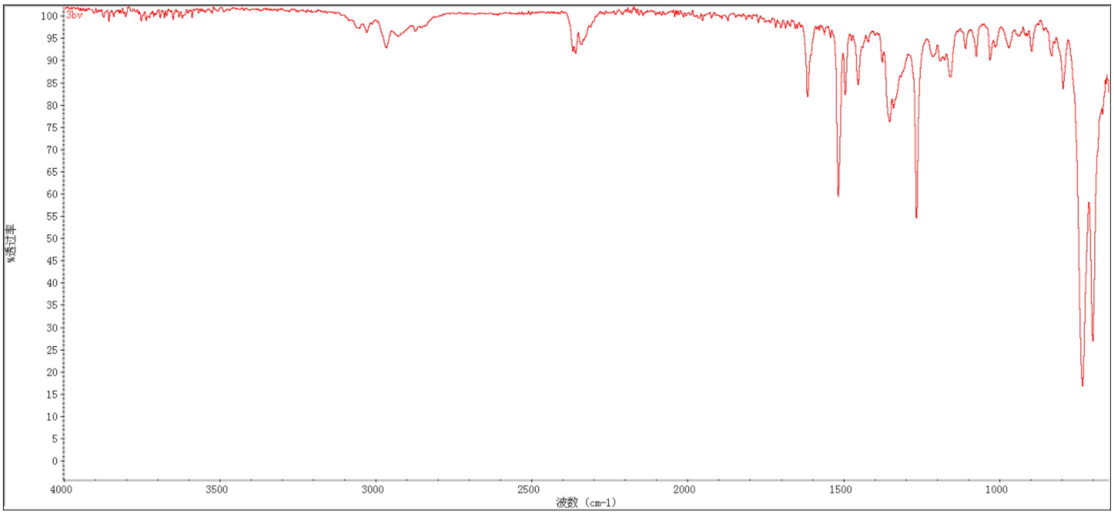

IR Spectra of **3b4**

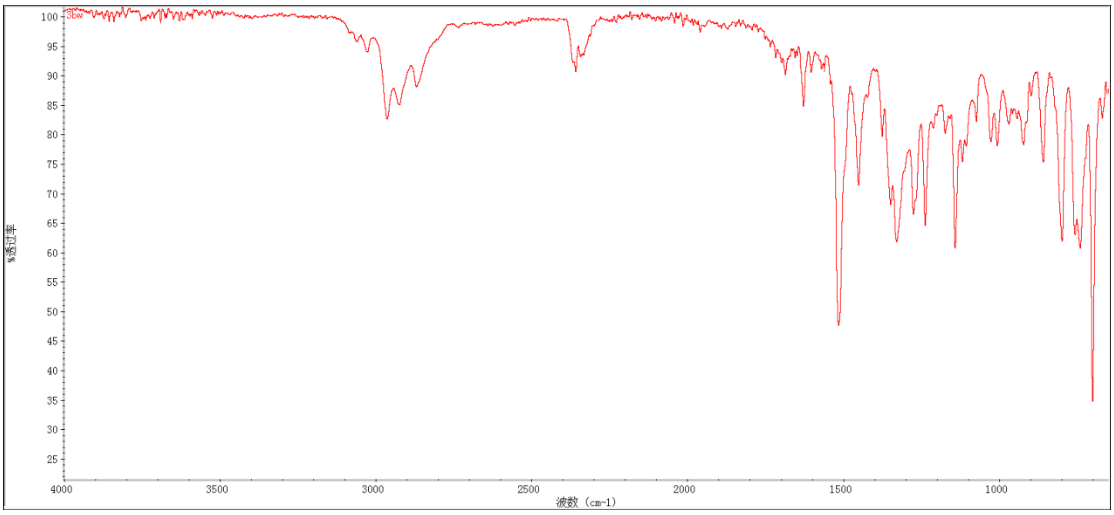

IR Spectra of **3b5**

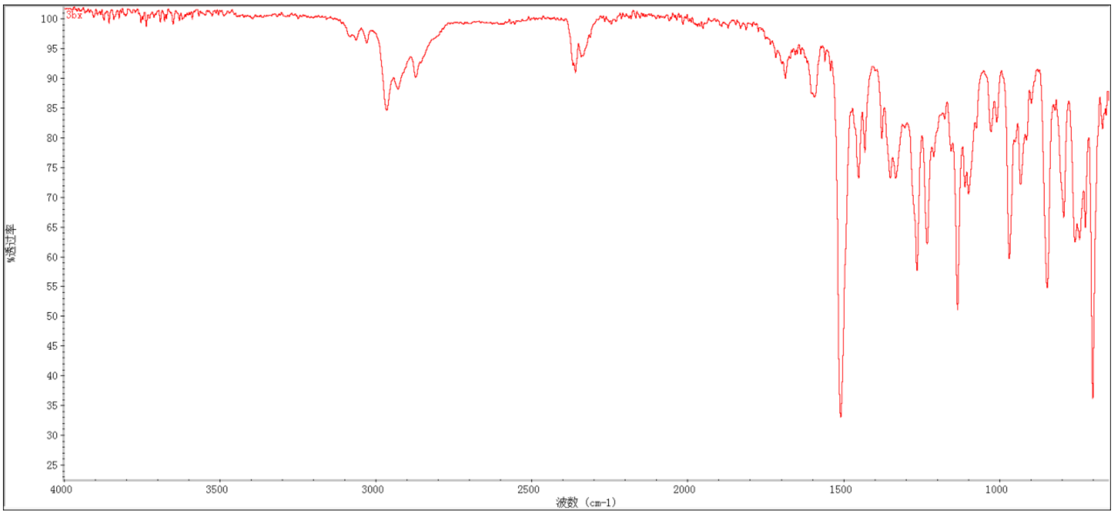

IR Spectra of **3b6**

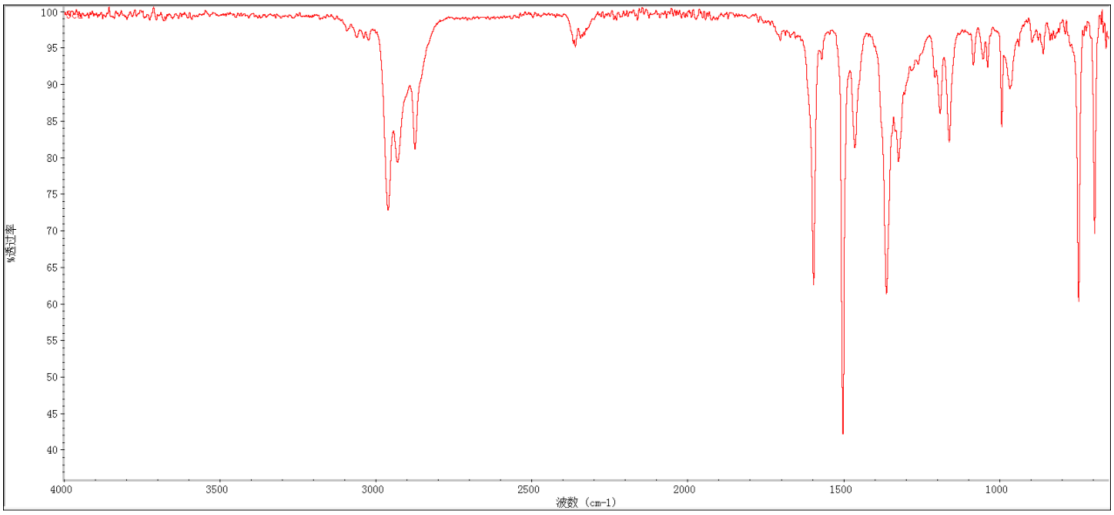

IR Spectra of **3c1**

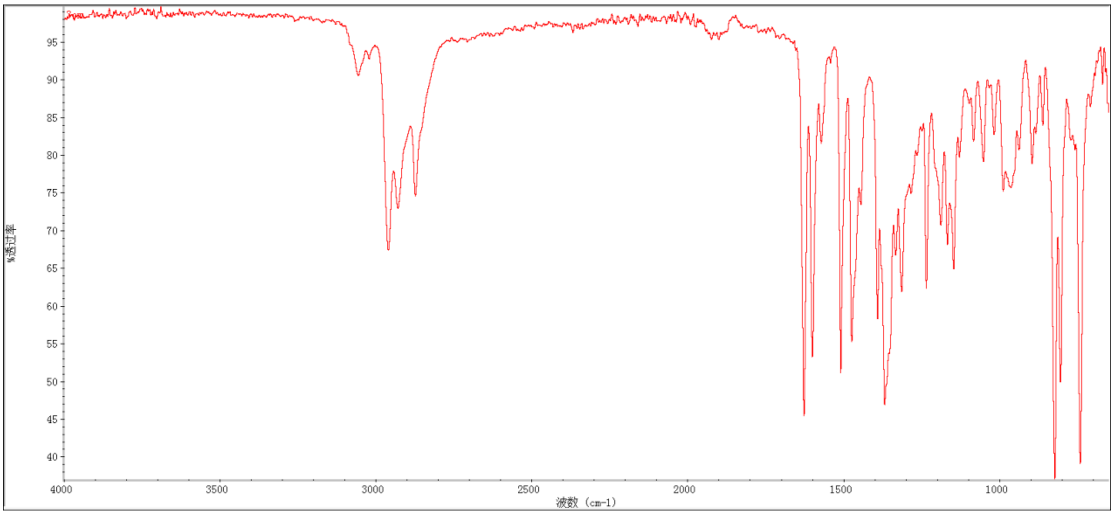

IR Spectra of **3c2**

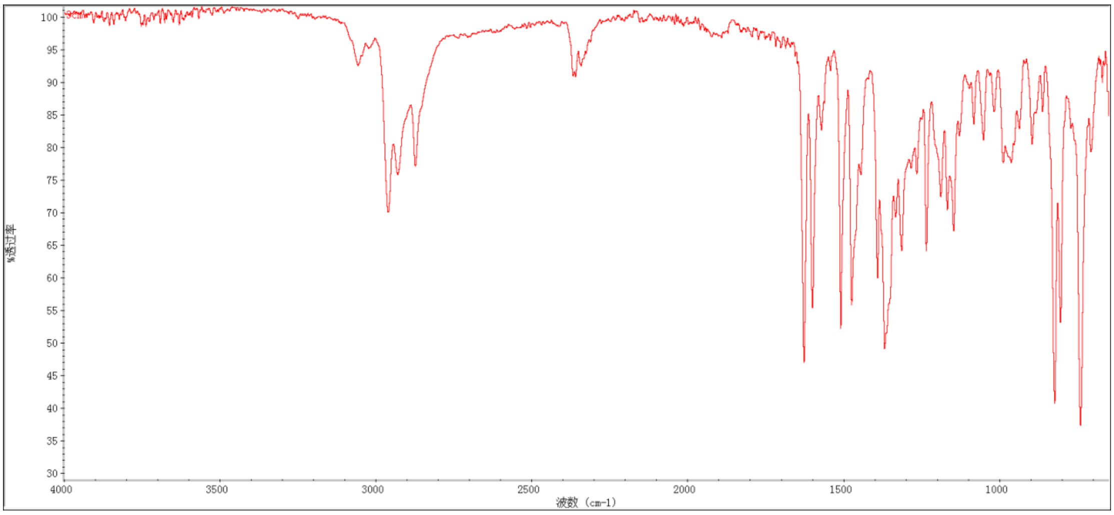

IR Spectra of **3c3**

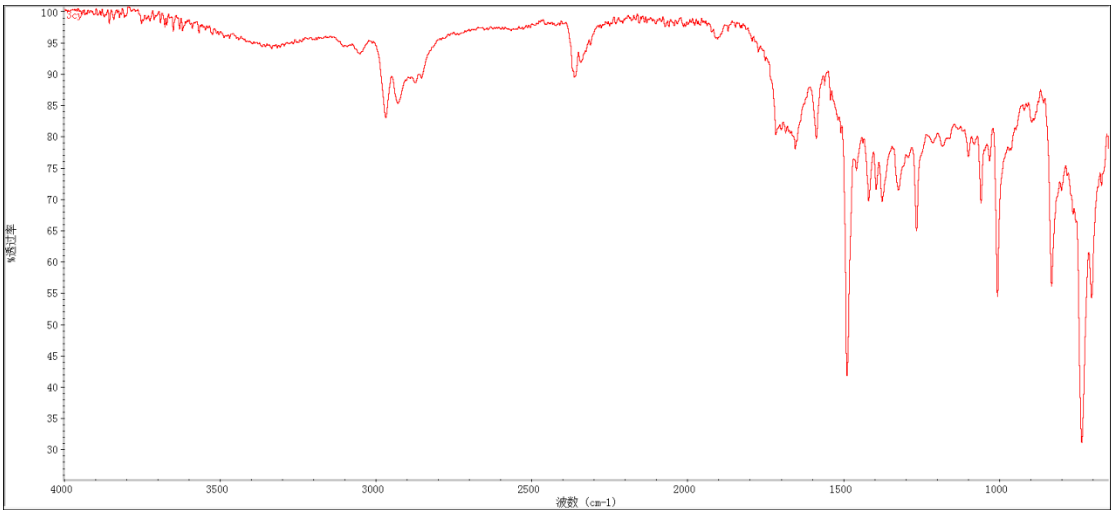

IR Spectra of **3c3’**
